# Remote care through telehealth for people with inflammatory bowel disease

**DOI:** 10.1002/14651858.CD014821.pub2

**Published:** 2023-05-04

**Authors:** Morris Gordon, Vassiliki Sinopoulou, Svetlana Lakunina, Teuta Gjuladin-Hellon, Kelly Bracewell, Anthony K Akobeng

**Affiliations:** School of MedicineUniversity of Central LancashirePrestonUK; Centre for GuidelinesNational Institute for Health and Care Excellence (NICE)ManchesterUK; University of Central LancashirePrestonUK; Pediatric GastroenterologySidra MedicineDohaQatar

**Keywords:** Adult, Child, Humans, Chronic Disease, Colitis, Ulcerative, COVID-19, Crohn Disease, Crohn Disease/therapy, Neoplasm Recurrence, Local, Quality of Life, Telemedicine

## Abstract

**Background:**

People with inflammatory bowel disease (IBD) require intensive follow‐up with frequent consultations after diagnosis. IBD telehealth management includes consulting by phone, instant messenger, video, text message, or web‐based services. Telehealth can be beneficial for people with IBD, but may have its own set of challenges. It is important to systematically review the evidence on the types of remote or telehealth approaches that can be deployed in IBD. This is particularly relevant following the coronavirus disease 2019 (COVID‐19) pandemic, which led to increased self‐ and remote‐management.

**Objectives:**

To identify the communication technologies used to achieve remote healthcare for people with inflammatory bowel disease and to assess their effectiveness.

**Search methods:**

On 13 January 2022, we searched CENTRAL, Embase, MEDLINE, three other databases, and three trials registries with no limitations on language, date, document type, or publication status.

**Selection criteria:**

All published, unpublished, and ongoing randomised controlled trials (RCTs) that evaluated telehealth interventions targeted at people with IBD versus any other type of intervention or no intervention.

We did not include studies based on digital patient information resources or education resources, unless they formed part of a wider package including an element of telehealth. We excluded studies where remote monitoring of blood or faecal tests was the only form of monitoring.

**Data collection and analysis:**

Two review authors independently extracted data from the included studies and assessed their risk of bias. We analysed studies on adult and paediatric populations separately. We expressed the effects of dichotomous outcomes as risk ratios (RRs) and the effects of continuous outcomes as mean differences (MDs) or standardised mean differences (SMDs), each with their 95% confidence intervals (CIs). We assessed the certainty of the evidence using GRADE methodology.

**Main results:**

We included 19 RCTs with a total of 3489 randomised participants, aged eight to 95 years. Three studies examined only people with ulcerative colitis (UC), two studies examined only people with Crohn's disease (CD), and the remaining studies examined a mix of IBD patients. Studies considered a range of disease activity states. The length of the interventions ranged from six months to two years. The telehealth interventions were web‐based and telephone‐based.

**Web‐based monitoring versus usual care**

Twelve studies compared web‐based disease monitoring to usual care.

Three studies, all in adults, provided data on disease activity. Web‐based disease monitoring (n = 254) is probably equivalent to usual care (n = 174) in reducing disease activity in people with IBD (SMD 0.09, 95% CI −0.11 to 0.29). The certainty of the evidence is moderate.

Five studies on adults provided dichotomous data that we could use for a meta‐analysis on flare‐ups. Web‐based disease monitoring (n = 207/496) is probably equivalent to usual care (n = 150/372) for the occurrence of flare‐ups or relapses in adults with IBD (RR 1.09, 95% CI 0.93 to 1.27). The certainty of the evidence is moderate. One study provided continuous data. Web‐based disease monitoring (n = 465) is probably equivalent to usual care (n = 444) for the occurrence of flare‐ups or relapses in adults with CD (MD 0.00 events, 95% CI −0.06 to 0.06). The certainty of the evidence is moderate. One study provided dichotomous data on flare‐ups in a paediatric population. Web‐based disease monitoring (n = 28/84) may be equivalent to usual care (n = 29/86) for the occurrence of flare‐ups or relapses in children with IBD (RR 0.99, 95% CI 0.65 to 1.51). The certainty of the evidence is low.

Four studies, all in adults, provided data on quality of life. Web‐based disease monitoring (n = 594) is probably equivalent to usual care (n = 505) for quality of life in adults with IBD (SMD 0.08, 95% CI −0.04 to 0.20). The certainty of the evidence is moderate.

Based on continuous data from one study in adults, we found that web‐based disease monitoring probably leads to slightly higher medication adherence compared to usual care (MD 0.24 points, 95% CI 0.01 to 0.47). The results are of moderate certainty. Based on continuous data from one paediatric study, we found no difference between web‐based disease monitoring and usual care in terms of their effect on medication adherence (MD 0.00, 95% CI −0.63 to 0.63), although the evidence is very uncertain. When we meta‐analysed dichotomous data from two studies on adults, we found no difference between web‐based disease monitoring and usual care in terms of their effect on medication adherence (RR 0.87, 95% CI 0.62 to 1.21), although the evidence is very uncertain.

We were unable to draw any conclusions on the effects of web‐based disease monitoring compared to usual care on healthcare access, participant engagement, attendance rate, interactions with healthcare professionals, and cost‐ or time‐effectiveness. The certainty of the evidence is very low.

**Authors' conclusions:**

The evidence in this review suggests that web‐based disease monitoring is probably no different to standard care in adults when considering disease activity, occurrence of flare‐ups or relapse, and quality of life. There may be no difference in these outcomes in children, but the evidence is limited. Web‐based monitoring probably increases medication adherence slightly compared to usual care.

We are uncertain about the effects of web‐based monitoring versus usual care on our other secondary outcomes, and about the effects of the other telehealth interventions included in our review, because the evidence is limited.

Further studies comparing web‐based disease monitoring to standard care for the clinical outcomes reported in adults are unlikely to change our conclusions, unless they have longer follow‐up or investigate under‐reported outcomes or populations. Studies with a clearer definition of web‐based monitoring would enhance applicability, enable practical dissemination and replication, and enable alignment with areas identified as important by stakeholders and people affected by IBD.

## Plain language summary

**The use of technology for remote care in inflammatory bowel disease**

**Key messages**

• Remote care is probably the same as usual care (e.g. face‐to‐face care in clinics and hospitals) for improving inflammatory bowel disease symptoms in adults; there is limited evidence for children. • Remote care is probably the same as usual care for avoiding relapses and flare‐ups; the same may be true for children. • Remote care is probably the same as usual care for improving quality of life in adults; there is limited evidence for children.

**What is inflammatory bowel disease?**

Inflammatory bowel disease refers to two main conditions that cause inflammation of the gut. These are ulcerative colitis and Crohn's disease. Ulcerative colitis only affects the large intestine. Crohn's disease can affect any part of the digestive tract, from mouth to bottom.

Inflammatory bowel disease mainly causes stomach pain or discomfort, diarrhoea that can be bloody, weight loss, and tiredness.

**What did we want to find out?**

Providing care from a distance, also called telehealth, is becoming more common, especially since the coronavirus 2019 (COVID‐19) pandemic. Using technology to provide remote care could benefit people with inflammatory bowel disease. Telehealth can take place via telephone, instant messaging, video, text message, web‐based services, or other means.

We wanted to find which communication technologies are used for remote care in inflammatory bowel disease, how they are used, if they are accessible to everyone, and what are their benefits or drawbacks.

**What did we do?**

We searched for randomised controlled trials (RCTs; studies where participants are randomly assigned to one of two or more treatment groups) comparing telehealth with any other treatment for people with inflammatory bowel disease. RCTs give us the highest standard of evidence.

We applied no limitations for age or type of remote care in our search, but we excluded studies that did not focus on providing care, such as studies providing only patient information or education. We also excluded studies that provided remote blood or stool test monitoring with no other type of remote monitoring.

**What did we find?**

We found 19 relevant RCTs, which enroled a combined total of 3489 people aged eight to 95 years. Remote care was delivered online (e.g. smartphone applications, websites) or by telephone.

Twelve studies compared web‐based care to usual care, three compared telephone‐based care to usual care, three compared web‐based care to "sham" care, one compared web‐based care to self‐care, and one compared psychological and telephone support to usual care.

Web‐based remote care is probably no different to usual care in adults for improving symptoms, avoiding relapses or flare‐ups, and enhancing quality of life.

We also found that people who receive web‐based care are probably less likely to skip their medicines compared to those that receive usual care. We are moderately certain about these results based on the current evidence.

The evidence on children is limited.

With the currently available information, we cannot make any judgements on other parameters such as access to care, whether people with inflammatory bowel disease approve of these programmes and are encouraged to attend appointments, to what degree clinical professionals are involved in them, and costs or time.

The evidence on other forms of remote care was also very limited.

**What are the limitations of the evidence?**

One limitation of the evidence was that the RCTs provided unclear descriptions of the remote care programmes, which means that any organisation wishing to copy and adopt these interventions would have difficulty doing so. The descriptions of usual care (the alternative treatment group in many studies) were also unclear. This means that standard care might be different from one study to another, which could make our findings less accurate.

Few studies looked at forms of remote care other than web‐based care.

Another limitation is that the different studies measured different results (outcomes) of treatment.

Finally, some studies used poor quality research methods.

**What next?**

No further studies comparing web‐based care to usual care in adults are necessary, unless they last for longer periods of time or give more details that would help clinicians adopt them anywhere in the world. This includes details on the type and number of staff needed, resources, equipment, costs, accessibility, and data security. More studies on children may be useful, as well as studies that examine differences based on sex and social or financial status. In any case, future studies should concentrate on measuring the results that matter most to people with inflammatory bowel disease and their care providers.

**How up‐to‐date is this review?**

This review is up‐to‐date as of January 2022.

## Summary of findings

**Summary of findings 1:** Web‐based disease monitoring compared to usual care

**Web‐based disease monitoring compared to usual care**						
**Patient or population:** people with inflammatory bowel disease **Setting:** hospitals and tertiary centres, and remotely **Intervention:** web‐based disease monitoring **Comparison:** usual care						
**Outcomes**	Anticipated absolute effects* (95% CI)		**Relative effect (95% CI)**	**№ of participants (studies)**	**Certainty of the evidence (GRADE)**	**Comments**
	**Risk with usual care**	**Risk with web‐based disease monitoring**				
**Disease activity (adults)** Follow‐up: 12 months	*—*	SMD 0.09 higher (0.11 lower to 0.29 higher)	*—*	428 participants (3 studies)	⊕⊕⊕⊝ **Moderate**^a^	Equivalent to a mean 36‐point reduction on the CDAI and a mean 1.7‐point reduction on the SCCAI
**Flare‐ups/relapse (dichotomous; adults)** Follow‐up: 6–12 months	**Study population**		**RR 1.09** (0.93 to 1.27)	868 participants (5 studies)	⊕⊕⊕⊝ **Moderate**^b^	*—*
	403 per 1000	440 per 1000 (375 to 512)				
**Flare‐ups/relapse (continuous; adults)** Follow‐up: 12 months	Mean number of flare‐ups was 0.19 (SD 0.42)	MD 0.00 more flare‐ups (0.06 fewer to 0.06 more)	*—*	909 participants (1 study)	⊕⊕⊕⊝ **Moderate**^a^	*—*
**Flare‐ups/relapse (dichotomous; children)** Follow‐up: 12 months	**Study population**		**RR 0.99** (0.65 to 1.51)	170 participants (1 study)	⊕⊕⊝⊝ **Low**^c^	*—*
	337 per 1000	334 per 1000 (219 to 509)				
**Quality of life (adults)** Follow‐up: 12 months	*—*	SMD 0.08 higher (0.04 lower to 0.20 higher)	*—*	1099 participants (4 studies)	⊕⊕⊕⊝ **Moderate**^d^	Equivalent to a mean 22‐point increase on the IBDQ scale
*The risk in the intervention group (and its 95% CI) is based on the assumed risk in the comparison group and the **relative effect** of the intervention (and its 95% CI). The comparison group risk has been calculated based on the data from the included studies. **CDAI:** Crohn's Disease Activity Index; **CI:** confidence interval; **IBDQ**: Inflammatory Bowel Disease Questionnaire; **MD:** mean difference; **RR:** risk ratio; **SCCAI:** Simple Clinical Colitis Activity Index; **SMD:** standardised mean difference; **SD:** standard deviation.						
**GRADE Working Group grades of evidence** **High certainty:** we are very confident that the true effect lies close to that of the estimate of the effect. **Moderate certainty:** we are moderately confident in the effect estimate; the true effect is likely to be close to the estimate of the effect, but there is a possibility that it is substantially different. **Low certainty:** our confidence in the effect estimate is limited; the true effect may be substantially different from the estimate of the effect. **Very low certainty:** we have very little confidence in the effect estimate; the true effect is likely to be substantially different from the estimate of effect.						

^a^ Downgraded once for risk of bias related to blinding. ^b^ Downgraded once for risk of bias related to blinding, selective reporting, and other sources. ^c^ Downgraded once for risk of bias related to blinding and imbalance in the numbers of participants reaching end of study, and once for imprecision due to low participant numbers. ^d^ Downgraded once for risk of bias related to blinding and attrition.

**Summary of findings 2:** Web‐based disease monitoring compared to sham monitoring

**Web‐based disease monitoring compared to sham monitoring**						
**Patient or population:** people with inflammatory bowel disease **Setting:** hospitals and tertiary centres, and remotely **Intervention:** web‐based disease monitoring **Comparison:** sham monitoring						
**Outcomes**	Anticipated absolute effects* (95% CI)		**Relative effect (95% CI)**	**№ of participants (studies)**	**Certainty of the evidence (GRADE)**	**Comments**
	**Risk with sham monitoring**	**Risk with web‐based disease monitoring**				
**Disease activity**	—	—	—	—	—	No data available
**Flare‐ups/relapse **	—	—	—	—	—	No data available
**Quality of life (adults)** Follow‐up: 6 months–2 years	1 study reported no changes in QoL. Another study reached no conclusion.		—	447 participants (2 studies)	⊕⊝⊝⊝ **Very low**^a^	—
*The risk in the intervention group (and its 95% CI) is based on the assumed risk in the comparison group and the **relative effect** of the intervention (and its 95% CI). The comparison group risk has been calculated based on the data from the included studies. **CI:** confidence interval; **QoL:** quality of life.						
**GRADE Working Group grades of evidence** **High certainty:** we are very confident that the true effect lies close to that of the estimate of the effect. **Moderate certainty:** we are moderately confident in the effect estimate; the true effect is likely to be close to the estimate of the effect, but there is a possibility that it is substantially different. **Low certainty:** our confidence in the effect estimate is limited; the true effect may be substantially different from the estimate of the effect. **Very low certainty:** we have very little confidence in the effect estimate; the true effect is likely to be substantially different from the estimate of effect.						

^a^ Downgraded once for serious risk of bias concerns (all domains) and twice for very serious imprecision due to very low event numbers.

**Summary of findings 3:** Web‐based disease monitoring compared to self‐screening

**Web‐based disease monitoring compared to self‐screening**						
**Patient or population:** people with inflammatory bowel disease **Setting:** hospitals and tertiary centres, and remotely **Intervention:** web‐based disease monitoring **Comparison:** self‐screening						
**Outcomes**	Anticipated absolute effects* (95% CI)		**Relative effect (95% CI)**	**№ of participants (studies)**	**Certainty of the evidence (GRADE)**	**Comments**
	**Risk with self‐screening**	**Risk with web‐based disease monitoring**				
**Disease activity (adults)** Follow‐up: 24 weeks	1 study reported no differences in disease activity.		—	102 participants (1 study)	⊕⊝⊝⊝ **Very low**^a^	—
**Flare‐ups/relapse (dichotomous; adults)** Follow‐up: 24 weeks	1 study reported no differences in relapses.		—	102 participants (1 study)	⊕⊝⊝⊝ **Very low**^a^	—
**Quality of life (adults)** Follow‐up: 24 weeks	1 study reported greater improvement in QoL in the control group.		—	102 participants (1 study)	⊕⊝⊝⊝ **Very low**^a^	—
*The risk in the intervention group (and its 95% CI) is based on the assumed risk in the comparison group and the **relative effect** of the intervention (and its 95% CI). The comparison group risk has been calculated based on the data from the included studies. **CI:** confidence interval; **QoL:** quality of life.						
**GRADE Working Group grades of evidence** **High certainty:** we are very confident that the true effect lies close to that of the estimate of the effect. **Moderate certainty:** we are moderately confident in the effect estimate; the true effect is likely to be close to the estimate of the effect, but there is a possibility that it is substantially different. **Low certainty:** our confidence in the effect estimate is limited; the true effect may be substantially different from the estimate of the effect. **Very low certainty:** we have very little confidence in the effect estimate; the true effect is likely to be substantially different from the estimate of effect.						

^a^ Downgraded once due to serious risk of bias concerns (randomisation, blinding, and selective reporting), and twice for very serious imprecision (very low participant and event numbers).

**Summary of findings 4:** Telephone‐based disease monitoring compared to face‐to‐face monitoring

**Telephone‐based disease monitoring compared to face‐to‐face monitoring **						
**Patient or population:** people with inflammatory bowel disease **Setting:** hospitals and tertiary centres, and remotely **Intervention:** telephone‐based disease monitoring **Comparison:** face‐to‐face monitoring						
**Outcomes**	Anticipated absolute effects* (95% CI)		**Relative effect (95% CI)**	**№ of participants (studies)**	**Certainty of the evidence (GRADE)**	**Comments**
	**Risk with face‐to‐face monitoring**	**Risk with telephone‐based disease monitoring**				
**Disease activity (adults)** Follow‐up: 6 months	1 study, whilst reporting no data on this outcome, mentioned there was no significant change.		—	60 participants (1 study)	⊕⊝⊝⊝ **Very low**^a^	—
**Flare‐ups/relapse (dichotomous; adults)** Follow‐up: 6 months	**Study population**		**RR 1.17** (0.47 to 2.89)	42 participants (1 study)	⊕⊝⊝⊝ **Very low**^b^	—
	286 per 1000	334 per 1000 (134 to 586)				
**Flare‐ups/relapse (dichotomous; children)** Follow‐up: 6 months	**Study population**		**RR 0.24** (0.03 to 2.05)	86 participants (1 study)	⊕⊝⊝⊝ **Very low**^b^	—
	95 per 1000	23 per 1000 (3 to 195)				
**Quality of life (adults)** Follow‐up: 6 months	1 study, whilst reporting no data on QoL, mentioned there was no significant change. Another study reported median QoL scores, which were not very different between groups.		—	123 participants (2 studies)	⊕⊝⊝⊝ **Very low**^a^	—
**Quality of life (children)** Follow‐up: 6 months	Mean of 106 points (SD 15.5) on the IMPACT QoL (35 lowest to 175 highest)	MD 7 points higher (0.29 lower to 14.29 higher)	—	86 (1 study)	⊕⊝⊝⊝ **Very low**^b^	—
*The risk in the intervention group (and its 95% CI) is based on the assumed risk in the comparison group and the **relative effect** of the intervention (and its 95% CI). The comparison group risk has been calculated based on the data from the included studies. **CI:** confidence interval; **MD:** mean difference; **QoL:** quality of life; **RR:** risk ratio.						
**GRADE Working Group grades of evidence** **High certainty:** we are very confident that the true effect lies close to that of the estimate of the effect. **Moderate certainty:** we are moderately confident in the effect estimate; the true effect is likely to be close to the estimate of the effect, but there is a possibility that it is substantially different. **Low certainty:** our confidence in the effect estimate is limited; the true effect may be substantially different from the estimate of the effect. **Very low certainty:** we have very little confidence in the effect estimate; the true effect is likely to be substantially different from the estimate of effect.						

^a^ Downgraded once for serious risk of bias concerns related to blinding and selective reporting, and twice for very serious imprecision due to very low participant numbers and events. ^b^ Downgraded one for serious risk of bias concerns related to blinding, and twice for very serious imprecision due to very low participant numbers.

**Summary of findings 5:** Cognitive behavioural therapy manual and telephone support compared to usual care

**Cognitive behavioural therapy manual and telephone support compared to usual care**						
**Patient or population:** people with inflammatory bowel disease **Setting:** hospitals and tertiary centres, and remotely **Intervention:** CBT manual and telephone support **Comparison:** usual care						
**Outcomes**	Anticipated absolute effects* (95% CI)		**Relative effect (95% CI)**	**№ of participants (studies)**	**Certainty of the evidence (GRADE)**	**Comments**
	**Risk with usual care**	**Risk with CBT manual and telephone support**				
**Disease activity**	—	—	—	—	—	No data available
**Flare‐ups/relapse**	—	—	—	—	—	No data available
**Quality of life**	—	—	—	—	—	No data available
*The risk in the intervention group (and its 95% CI) is based on the assumed risk in the comparison group and the **relative effect** of the intervention (and its 95% CI). The comparison group risk has been calculated based on the data from the included studies. **CBT:** cognitive behavioural therapy; **CI:** confidence interval.						
**GRADE Working Group grades of evidence** **High certainty:** we are very confident that the true effect lies close to that of the estimate of the effect. **Moderate certainty:** we are moderately confident in the effect estimate; the true effect is likely to be close to the estimate of the effect, but there is a possibility that it is substantially different. **Low certainty:** our confidence in the effect estimate is limited; the true effect may be substantially different from the estimate of the effect. **Very low certainty:** we have very little confidence in the effect estimate; the true effect is likely to be substantially different from the estimate of effect.						

## Background

### Description of the condition

Inflammatory bowel disease (IBD) is an umbrella term that encompasses three main disease subtypes that affect the gastrointestinal tract: ulcerative colitis (UC), Crohn's disease (CD), and IBD unclassified. IBS prevalence exceeds 0.3% in Europe, North America, and Oceania; and incidence is rapidly rising in newly industrialised countries (Ng 2017). It has no known cure but can be managed; therefore, it places a huge financial burden on healthcare systems (Ghosh 2015). Approximately 25% of cases are diagnosed before 18 years of age, and the main treatment modalities are pharmacological therapy, dietary therapy, and surgery. Guided management and care can improve disease activity, symptoms, clinical outcomes (e.g. need for surgery), and quality of life (QoL; Elkjaer 2012). After diagnosis, intensive follow‐up and frequent consultations are required to optimise IBD care, at least for some stages of the disease course (Bernstein 2011).

### Description of the intervention

IBD telehealth management refers to the remote delivery of healthcare management from the healthcare professional to the person with IBD (McLean 2011). It includes consulting by phone, instant messenger, video, text message, or web‐based services. Communication can be live, such as by telephone, or delayed, such as by email (McLean 2009). During a telehealth session, the person with IBD provides information about their condition and health status. The information becomes electronically available to the clinician or other healthcare professional, who uses it to provide feedback based on their professional judgement (McLean 2011; Sood 2007). Telehealth can be beneficial for certain subgroups of people with IBD who might face problems accessing traditional healthcare resources that require their physical presence, such as older people, people from socio‐economically disadvantaged backgrounds, and people with physical or learning disabilities. However, these subgroups may face a separate set of barriers to accessing telehealth resources (Choi 2014; Forducey 2012; Rimmer 2013). Telehealth is not synonymous with telemedicine, which "refers to the use of live synchronised videoconferencing, allowing for interactive video communications between a provider and a patient" (Groom 2021).

### How the intervention might work

Telehealth consultations work similarly to face‐to‐face consultations; the only difference is that any procedure that requires the patient's physical presence cannot occur (e.g. blood tests or physical examination; Heida 2018). Therefore, while telehealth consultations might be a useful substitute when face‐to‐face consultations are not possible or recommended, it is unknown how effective they are compared to face‐to‐face consultations. The breadth of available telehealth options also means that each option has its own advantages and disadvantages.

Telehealth consultations may reduce potential barriers to multidisciplinary team communication across team members and organisations and achieve successful communication in real time. This could facilitate more timely data monitoring and sharing of questions and concerns voiced by the person with IBD among the entire multidisciplinary team, including the primary care professionals (Cross 2012).

### Why it is important to do this review

It is important to systematically review the evidence on the effects of remote or telehealth approaches that can be deployed for IBD care. This has become particularly relevant since the coronavirus 19 (COVID‐19) pandemic and resulting need for increased self‐management and remote management, which these interventions can facilitate (Al‐Ani 2020). It is also key to ascertain the effective components of remote or telehealth packages so that they can be replicated and disseminated.

## Objectives

To identify the communication technologies used to achieve remote healthcare for people with inflammatory bowel disease and to assess their effectiveness.

## Methods

### Criteria for considering studies for this review

#### Types of studies

All published, unpublished, and ongoing randomised controlled trials (RCTs) that evaluated telecommunication technologies for the management of IBD versus face‐to‐face interventions or no intervention. Cross‐over studies and cluster‐RCTs were eligible for inclusion, but quasi‐randomised trials (using inappropriate randomisation) were ineligible.

We did not include studies on digital patient information resources (e.g. information on IBD organisation websites, such as Crohn's and Colitis UK), or education resources alone, unless they formed part of a wider package that included an element of telehealth as defined in this review. A separate Cochrane Review is focussing on education resources for people with IBD (Gordon 2021a).

We excluded studies where remote monitoring of blood or faecal tests was the only form of monitoring.

#### Types of participants

People of all ages with a confirmed IBD diagnosis. Subsets such as CD, UC, or intermediate colitis were eligible.

#### Types of interventions

We included studies on IBD management interventions that took place via phone, instant messaging, video, text message, or web‐based services, or any other means of remote communication, whether live (e.g. telephone conversations) or delayed (e.g. email).

We considered any control intervention, such as face‐to‐face interventions, no intervention. Studies that compared different telehealth interventions to each other were also eligible.

We aimed to perform separate analyses for trials that evaluated telehealth plus traditional consultations versus traditional consultations alone and trials that evaluated telehealth versus traditional consultations.

#### Types of outcome measures

Our review included dichotomous and continuous outcome measures. Study outcomes were irrelevant for determining study eligibility.

##### Primary outcomes

- Disease activity at study end, using a recognised disease activity scoring system, measured clinically, endoscopically, or histologically, and as defined by study authors (separate for adults and children, if sufficient data available). We planned to analyse clinical, endoscopic, and histological data separately.
- Flare‐ups or relapses at study end, measured clinically, endoscopically, or histologically, and as defined by study authors (separate for adults and children, if sufficient data available). We planned to analyse clinical, endoscopic, and histological data separately.
- QoL at study end, using validated scales or tools, and as defined by study authors (separate for adults and children, if sufficient data available)

##### Secondary outcomes

- Number of episodes of accessing healthcare (outpatient, remote, or inpatient) at study end, as defined by study authors
- Medication adherence at study end, as defined and measured by study authors
- Participant engagement (adherence/compliance) with the intervention at study end, as defined by study authors
- Rate of attendance or engagement with any or all elements of the intervention (number of planned appointments attended, number of planned interactions attended) at study end, as defined by study authors
- Rate of attendance of interactions with healthcare professionals during the intervention (as part of the intervention or otherwise), as defined by study authors
- Costs or cost/time‐effectiveness during study, as defined by study authors

##### Qualitative outcomes

- Programme attributes (technology type, design, cost, user guidance, live contact, management of delayed contact, contact with other members of the multidisciplinary team, time to response, data security) during study
- Programme requirements (cost, software, infrastructure, training needs, access requirements (for the person with IBD and the healthcare provider)) during study

### Search methods for identification of studies

#### Electronic searches

We searched the following databases from inception, applying no restrictions on the language of publication.

- Cochrane Central Register of Controlled Trials (CENTRAL; 2022, Issue 1) via Ovid Evidence‐Based Medicine Reviews Database (EBMR; searched 13 January 2022; Appendix 1)
- MEDLINE and MEDLINE ALL via Ovid (1946 to 13 January 2022; Appendix 2)
- Embase via Ovid (1974 to 13 January 2022; Appendix 3)
- PsycINFO via Ovid (1806 to 13 January 2022; Appendix 4)
- CINAHL via EBSCO (1937 to 13 January 2022; Appendix 5)
- AMED (Allied and Complementary Medicine database) via Ovid (1985 to 13 January 2022; Appendix 6)

We searched the following trial registries by combining terms related to IBD and telehealth.

- Cochrane Gut Group Specialised Register
- ClinicalTrials.gov (www.clinicaltrials.gov; Appendix 7)
- World Health Organization (WHO) International Clinical Trials Registry Platform (ICTRP;trialsearch.who.int/; Appendix 8)

#### Searching other resources

As complementary search methods, we carefully checked the references of included studies and relevant systematic reviews for other potentially eligible studies. We sought unpublished trials by contacting experts in the field, and we scanned relevant conference abstracts that were identified in the search (Embase and CENTRAL) to capture any studies presented but not yet published in full.

We attempted to obtain translations of papers when necessary.

### Data collection and analysis

We carried out data collection and analysis according to the methods recommended in the *Cochrane Handbook for Systematic Reviews of Interventions* (Higgins 2020).

#### Selection of studies

Two review authors independently screened the titles and abstracts identified from the literature search, discarding studies that were clearly irrelevant. We obtained the full reports of all potentially eligible studies, and two review authors independently assessed them against our inclusion criteria. We resolved disagreements by discussion, or by consulting a third review author where necessary. We presented studies excluded at this or subsequent stages in the Characteristics of excluded studies table and recorded the main reason for exclusion. We outlined the selection process in a PRISMA flowchart (Page 2021).

#### Data extraction and management

Two review authors independently extracted data from the included studies using piloted data extraction forms. We collected the following variables, where available.

- Trial setting: country and number of trial centres
- Trial registration details: registration number, date of registration, registered outcomes
- Methods: study design, total study duration, dates
- Participant characteristics: age, socio‐demographics, ethnicity, disease status, disease type, diagnostic criteria, total number
- Eligibility criteria: inclusion and exclusion criteria
- Intervention and comparator: type of telehealth and control intervention, people delivering the intervention, resources required to deliver the intervention, time to response, people with access to the intervention, data security
- Outcomes: outcome definition, unit of measurement, time of collection
- Results: number of participants allocated to each group, missing participants, sample size
- Funding source and conflicts of interest

For studies requiring translation, we used online translation software or, if necessary, we sought translations by speakers of the relevant languages.

#### Assessment of risk of bias in included studies

During data extraction, two review authors independently assessed all included studies for risk of bias, using the Cochrane risk of bias tool (RoB 1), as outlined in the C*ochrane Handbook for Systematic Reviews of Interventions* (Higgins 2011). RoB 1 includes the following risk of bias domains.

- Sequence generation (selection bias)
- Allocation concealment (selection bias)
- Blinding of participants and personnel (performance bias)
- Blinding of outcome assessment (detection bias)
- Incomplete outcome data (attrition bias)
- Selective reporting (reporting bias)
- Other bias

We judged the studies to be at low, high, or unclear risk of bias for each domain assessed.

After data extraction, two review authors compared the extracted data to discuss and resolve discrepancies before transferring the data to the Characteristics of included studies table in Review Manager Web (RevMan Web 2022).

We judged risk of bias for cluster‐RCTs as prescribed in Section 16.3.2 of the C*ochrane Handbook for Systematic Reviews of Interventions* (Higgins 2011).

#### Measures of treatment effect

For dichotomous outcomes, we expressed the treatment effect as risk ratios (RRs) with corresponding 95% confidence intervals (CIs). For continuous outcomes, we expressed the treatment effect as mean differences (MDs) with 95% CIs. However, if studies assessed the same continuous outcome on a different scale, we estimated the treatment effect using the standardised mean difference (SMD). We presented SMDs as standard deviation (SD) units and interpreted them as follows: 0.2 represents a small effect, 0.5 a moderate effect, and 0.8 a large effect.

#### Unit of analysis issues

The participant was the unit of analysis. For studies comparing more than two intervention groups, we made multiple pair‐wise comparisons between all possible pairs of intervention groups. To avoid double counting, we divided shared intervention groups evenly among the comparisons. For dichotomous outcomes, we divided both the number of events and the total number of participants. For continuous outcomes, we only divided the total number of participants, and left the means and SDs unchanged.

We pooled data from cross‐over studies if they were reported separately before and after cross‐over (we only used data from before cross‐over). For cluster‐RCTs, we only used study data if the study authors had used appropriate statistical methods for taking the clustering effect into account.

If studies reported dichotomous event data per episode instead of per participant, we contacted the study authors for further data to avoid unit of analysis issues. If studies reported outcomes at several time points, we used the longest follow‐up.

#### Dealing with missing data

We contacted study authors to request missing data where necessary.

For analyses of dichotomous outcomes, we used the numbers randomised as denominators and numbers of events as numerators. For analyses of continuous outcomes, we used the sample numbers as reported by the study authors for each particular continuous outcome. If the sample numbers were not reported, we estimated them based on reported attrition percentages. We attempted to estimate missing SDs using relevant statistical tools and calculators if studies reported other variance measures.

Studies that did not report measures of variance were judged at high risk of selective reporting.

We used the same methods in our sensitivity analyses.

#### Assessment of heterogeneity

We scrutinised studies to ensure they were clinically homogenous in terms of participants, interventions, comparators, and outcomes. To test for statistical heterogeneity, we used a Chi² test, considering a P value below 0.1 indicative of heterogeneity. To quantify statistical heterogeneity, we used the I² statistic, interpreting the values according to the following thresholds (Higgins 2020).

- 0% to 40%: might not be important
- 30% to 60%: may represent moderate heterogeneity
- 50% to 90%: may represent substantial heterogeneity
- 75% to 100%: considerable heterogeneity

We examined possible explanations for heterogeneity when sufficient data were available, including factors such as participant characteristics (e.g. age, sex), condition severity, healthcare system, and country.

Where we detected a considerable degree of statistical heterogeneity (I² value above 75%), we did not pool the data in a meta‐analysis. We also investigated possible sources of considerable statistical heterogeneity (e.g. clinical differences, risk of bias) and conducted sensitivity analyses where relevant. If we were unable to explain considerable statistical heterogeneity, we presented the results narratively.

#### Assessment of reporting biases

We used an inclusive search strategy in an attempt to minimise reporting biases. Had we included 10 or more studies in a meta‐analysis, we would have investigated publication bias by creating a funnel plot and visually inspecting funnel plot asymmetry, or by following other methods described in the *Cochrane Handbook of Systematic Reviews* (Higgins 2020). We would also have tested funnel plot asymmetry by performing a linear regression of the intervention effect estimate against its standard error, weighted by the inverse of the variance of the intervention effect estimate (Egger 1997).

#### Data synthesis

We summarised the study characteristics narratively, then performed meta‐analyses where two or more studies assessed similar populations, interventions, and outcomes. We planned to perform separate analyses of studies on paediatric populations, adult populations, and different sub‐intervention types, using Review Manager Web (RevMan Web 2022). We synthesised data using the random‐effects model. We pooled RRs for dichotomous outcomes and MDs or SMDs for continuous outcomes, alongside 95% CIs. When we were unable to carry out a meta‐analysis (e.g. due to lack of uniformity in data reporting), we presented a narrative summary of the included studies.

We grouped qualitative outcomes by the key attributes defined in Secondary outcomes, and presented them in additional tables. We also presented summary descriptive statistics (number of specific remote telehealth solutions used, mean costs, resources, etc.) to help readers ascertain the core attributes across studies. We presented these data narratively and in additional tables.

#### Subgroup analysis and investigation of heterogeneity

Where we detected heterogeneity, we investigated possible causes and addressed them using methods described in Higgins 2020.

For our primary outcomes, we presented our analyses separately based on age (adult/paediatric), and we undertook subgroup analyses based on disease type, which we considered the variable most likely to impact outcomes differently.

The statistical methods described in Data synthesis applied to the subgroup analyses.

#### Sensitivity analysis

Where possible, we planned to undertake sensitivity analyses on the primary outcomes to assess whether the findings of the review were robust to the decisions made during the review process. In particular, we intended to exclude studies at high or unclear risk of selection and performance bias. Where analyses included studies with reported and estimated SDs, we planned to exclude those with estimated SDs, to assess whether this exclusion would affect the findings of the review. We investigated whether the choice of model (fixed‐effect versus random‐effects) impacted the results, and we explored heterogeneity in case of major inconsistencies between the results of the two models.

#### Summary of findings and assessment of the certainty of the evidence

We presented the main results for all comparisons in summary of findings tables. We exported data for each comparison and primary outcome to GRADEpro software to assess the certainty of the evidence (GRADEpro GDT). We included all three primary outcomes in the summary of findings tables. We considered that the most important outcomes for decision‐makers were those from the comparison 'web‐based disease monitoring versus usual care'.

Based on risk of bias, inconsistency, imprecision, indirectness, and publication bias, we rated the certainty of the evidence for each outcome as high, moderate, low, or very low. The GRADE Working Group has defined these ratings as follows.

- High certainty: we are very confident that the true effect lies close to that of the estimate of the effect.
- Moderate certainty: we are moderately confident in the effect estimate; the true effect is likely to be close to the estimate of the effect, but there is a possibility that it is substantially different.
- Low certainty: our confidence in the effect estimate is limited; the true effect may be substantially different from the estimate of the effect.
- Very low certainty: we have very little confidence in the effect estimate; the true effect is likely to be substantially different from the estimate of effect.

We justified all decisions to downgrade the certainty of the evidence using footnotes, and made comments to aid the reader's understanding of the review where necessary.

## Results

### Description of studies

The Characteristics of included studies table, Characteristics of excluded studies table, Characteristics of studies awaiting classification table, and Characteristics of ongoing studies table provide detailed information.

#### Results of the search

We completed our literature search on 13 January 2022, identifying 3946 records through database searching and three additional records from alternative sources. After removal of duplicates, 2622 unique records remained. After title and abstract screening, we retrieved 132 full‐text articles; of these, 70 reports of 19 RCTs met our eligibility criteria. Figure 1 presents the study selection process in a PRISMA flow diagram.

**1:**
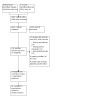
Flow chart of study retrieval and selection.

#### Included studies

For details of study and participant characteristics, see Table 6.

**1:** Study and participant details

**Study ID**	**Trial registration**	**Disease type^a^**	**Disease state (relapse/remission)**	**Numbers randomised **	**Concurrent therapies^a^**	**Ethnicity^a^**	**Socio‐economic status^a^**	**Conflicts of interest**	**Funding**
**Akobeng 2015**	NCT02319798	Mixed IBD CD: IG: 36; CG: 35 UC/IC: IG: 8; CG: 7	Remission	IG: 44 CG: 42	NR	NR	NR	"The authors report grants from Research for Patient Benefit Programme, UK National Institute for Health Research, during the conduct of the study"	"The project was funded by Research for Patient Benefit Programme, UK National Institute for Health Research (grant number PB‐PG‐0408‐16218)."
**Ankersen 2019**	NCT02492555	Mixed IBD CD: IG: 13 (26%); CG: 10 (19.2%) UC: IG: 35 (70%); CG: 39 (75%)	Remission or mild‐moderate disease activity	IG: 50 CG: 52	None: IG: 9 (18.0%); CG: 10 (19.2%) 5‐ASA: IG: 27 (54.0%); CG: 24 (46.2%) Corticosteroids: IG: 4 (8.0%); CG: 4 (7.7%) Immunomodulators: IG: 3 (6.0%); CG: 9 (17.3%) Biological therapy: IG: 7 (14.0%); CG: 5 (9.6%)	NR	Length of education after high school: Short: IG: 2; CG: 4 Medium: IG: 40; CG: 31 Higher/academic: IG: 6; CG: 13 Occupation: Yes: IG: 38; CG: 42 No: IG: 12; CG: 10	"Ankersen DV has received grants from Ferring Pharmaceuticals, Crohn Colitis patient society Denmark, North Zealand University Hospital and nonfinancial support from Calpro AS; Weimers P has received grants from Ferring lægemidler and Tillotts Pharma AG as well as nonfinancial support from Janssen‐ Cilag A/S, Calpro AS, and Vifor Pharma Nordiska AB; Marker D has received non‐financial support from Calpro AS and Pharmacosmos; Bennedsen M has received other financial support from AbbVie, Tillotts, Takeda, MSD and Pfizer; Saboori S has received non‐financial support from Janssen‐Cilag and Salofalk; Paridaens K is an employee of Ferring Pharmaceuticals; Burisch J has received grants from AbbVie, Takeda, Tillotts Pharma and personal fees from AbbVie, Janssen‐Cilag, Celgene, Samsung Bioepis, MSD, Pfizer and Takeda; Munkholm P has none to declare."	"Calpro AS; CrohnColitis patient society Denmark; and North Zealand UniversityHospital and FerringPharmaceuticals."
**Atreja 2018**	NCT02322307	Mixed IBD	Unclear	IG: 162 CG: 158	NR	White: 82.2% Black: 5.3% Hispanic: 9.1%	College education	NR	"The study is supported by the Crohn's & Colitis Foundation of America (grant #253624) and the National Institutes of Health (5K23 DK97451‐02)."
**Carlsen 2017**	NCT01860651	Mixed IBD CD: IG: 8; CG 13 UC: IG: 19; CG: 13	CD (remission): IG: 2; CG: 5 CD (mild): IG: 5; CG: 6 CD (moderate): IG: 0; CG: 2 CD (severe): IG: 1; CG: 0 UC (remission): IG: 14; CG: 9 UC (mild): IG: 5; CG: 4	IG: 27 CG: 26	NR	Ethnicity is reported in the trial registration, but not in the paper.	NR	None	"European Crohn’s and Colitis Organization, Queen Louise’s Hospital Foundation, TrygFoundation, CALPRO A/S, Tillotts Pharma, Capital Region Denmark, Alice and Frimodts Foundation, Ulcerative colitis and Crohn’s Danish Patient Society, and Merck Sharp and Dome."
**Chauhan 2016**	NA	Mixed IBD	NR	IG+CG: 60	NR	NR	NR	NR	NR
**Cross 2012**	NCT00620126	UC	Mixed: remission and active disease	IG: 25 CG: 22	Steroids: Total: 5; IG: 3; CG: 2 Immune suppressants: Total: 20; IG: 14; CG: 6 Infliximab: Total: 14; IG: 7; CG: 7	White: Total: 31; IG: 16; CG: 15 Other: Total: 16; IG: 9; CG: 7	Disease knowledge: Limited: Total: 7; IG: 4; CG: 3 Good: Total: 30; IG: 15; CG: 15 Excellent: Total: 10; IG: 4; CG: 6	NR	"Broad Medical Research Program (BRMP‐0190), University of Maryland General Clinical Research Center Grant (M01 RR 16500), General Clinical Research Centers Program, National Center for Research Resources (NCRR), NIH, and the Baltimore Education and Research Foundation."
**Cross 2019**	NCT01692743	CD: IG1: 79; IG2: 78; CG: 79 UC/IC: IG1: 36; IG2: 38; CG: 38	Mixed, remission (148) and active disease (200)	IG1: 115 IG2: 116 CG: 117	Aminosalicylates: Total: 108; IG1: 29; IG2: 39; CG: 40 Corticosteroids: Total: 64; IG1: 17; IG2: 27; CG: 20 Mercaptopurine/azathioprine: Total: 111; IG1: 33; IG2: 42; CG: 36 Anti‐TNF: Total: 206; IG1: 66; IG2: 68; CG: 72	White: Total: 319; IG1: 108; IG2: 111; CG: 100 African American: Total: 24; IG1: 5; IG2: 5; CG: 14 Asian: Total: 1; IG1: 1; IG2: 0; CG: 0 Other: Total: 3; IG1: 1; IG2: 0; CG: 0	Insurance status: None: Total: 14; IG1: 0; IG2: 1; CG: 13 Medical assistance: Total: 6; IG1: 1; IG2: 2; CG: 3 Medicare: Total: 15; IG1: 6; IG2: 1; CG: 8 Commercial: Total: 198; IG1: 67; IG2: 70; CG: 61 Other: Total: 64; IG1: 24; IG2: 27; CG: 13	"None"	"Agency for Healthcare Research and Quality (1R01HS018975‐01A1) and the University of Maryland general clinical research centers program."
**De Jong 2017**	NCT02173002	Mixed IBD	Mixed Remission: IG: 394; CG: 380 Active: IG: 71; CG: 64	IG: 465 CG: 444	No medication/mesalazine: IG: 147; CG: 173 Immunosuppresants: IG: 131; CG: 122 Biologics: IG: 166; CG: 170	NR	Education: University: IG: 54; CG: 49 Higher vocational education: IG: 103; CG: 98 Intermediate vocational education: IG: 160; CG: 157 Secondary edication: IG: 56; CG: 55 Primary education: IG: 6; CG: 8 Missing data: IG: 86; CG: 77	"MJdJ reports non‐financial support from Merck Sharpe & Dohme, outside the submitted work. AEvdM‐dJ reports grants and non‐financial support from Takeda, personal fees from AbbVie, and non‐financial support from Tramedico, all outside the submitted work. AAvB reports personal fees from AbbVie, MSD, Ferring, Tramedico, Takeda, Pfizer, and Janssen, all outside the submitted work. GD reports speaker’s fees from Shire, AbbVie, and Takeda, and a grant for investigator‐initiated research from Takeda, all outside the submitted work. AAM reports grants from Grünenthal, Zon MW GGG (government), Will Pharma, BioActor, Pentax Europe, Falk Pharma, and Almiral Pharma, all outside the submitted work. AB received research grants to her department from AbbVie, Amgen, and Merck, and advisory board honoraria from Janssen and Sandoz, all unrelated to the current work. MJP reports personal fees from AbbVie, Ferring, Janssen, and Takeda, and grants from Falk, all outside the submitted work. All other authors declare no competing interests."	"Academic incentive fund of the Maastricht University Medical Centre (31962340B)."
**Del Hoyo 2018**	NCT02943538	CD: IG1: 13/21; IG2: 13/21; CG: 14/21 UC: IG1: 8/21; IG2: 8/21; CG: 7/21	Remission and active Remission: CD: IG1: 6; IG2: 9; CG: 10 UC: IG1: 2; IG2: 1; CG: 2	IG1: 21 IG2: 21 CG: 21	Immunomodulators: IG1: 10; IG2: 9; CG: 10 Biologics: IG1: 4; IG2: 4; CG: 4 Combination therapy: IG1: 5; IG2: 6; CG: 6 Corticosteroids: IG1: 2; IG2: 2; CG: 1	NR	Education: Primary education: 9/30; secondary education: 21/30; university: 29/30 Work Productivity and Activity Impairment: Not working: IG1: 7/21; IG2: 5/21; CG: 8/21 Percentage of work hours missed: IG1: median 40% (IQR 15%–62.5%); IG2: median 32.5% (IQR 7.5%–57.5%); CG: median 27.5% (IQR 0%–52%) Work impairment score: IG1: median 7 (IQR 3–10); IG2: median 10 (IQR: 2.25–10); CG: median 7 (IQR 2.75–10) Social impairment score: IG1: median 3.5 (IQR 2–7); IG2: median 6 (IQR 2.75–8); CG: median 3.5 (IQR 1–5.75) Satisfaction score: CG: median 49.5 (IQR 42.5–53.75); IG1: median 53 (IQR 50–59); IG2: median 52 (IQR 47.5–55)	"DD is the general manager of Connected Health Services."	"Grants from the Instituto de Salud Carlos III‐Fondo de Investigaciones Sanitarias (FIS PI12/00277) and cofunded by FEDER (Fondo Europeo de Desarrollo Regional)."
**Elkjaer 2010**	NR	UC	Mild/moderate disease	IG: 117 CG: 116	5‐ASA systemic: Asacol: IG: 78; CG: 68 Pentasa: IG: 8; CG: 7 Dipentum: IG: 2; CG: 4 Premid: IG: 2; CG: 2 Salazopyrin: IG: 3; CG: 6 Mezavant: IG: 0; CG: 0 None: IG: 12; CG: 19 Suppositories: Asacol: IG: 3; CG: 2 Pentasa: IG: 12; CG: 9 Mesasal: IG: 3; CG: 1 Prednisolon: IG: 1; CG: 0 None: IG: 88; CG: 94 Enema /Foam: Asacol: IG: 4; CG: 4 Pentasa: IG: 7; CG: 6 Colifoam: IG: 4; CG: 4 Pred‐clysma: IG: 0; CG: 0 None: IG: 90; CG: 92	MR	Marital status: Married: IG: 69/105: CG: 82/106 Single: IG: 36/105; CG: 24/106 Education: Academic: IG: 33/105; CG: 29/106 in CG Other education: IG: 55/105; CG: 64/106 During education: IG: 16/105; CG: 5/106 No education: IG: 1/105; CG: 8/106 Occupation: Paid: IG: 82/105; CG: 86/106 Unpaid: IG: 1/105; CG: 4/106 Support: IG: 15/105; CG: 6/106 Pensioner: IG: 7/105; CG: 10/106	"PM is member of the advisory boards in Ferring, Tillots, MSD and Swedish Orphan. ME is member of the advisory board in Swedish Orphan. HS is member of the advisory board in Swedish Orphan. CO’M is on the International Advisory Board of Abbott, MSD, and Shire Pharmaceutical Company. He has unrestricted educational grants from Abbott and MSD"	"Colitis Crohn Patient Organisation, Moran’s Foundation, Vibeke Binder & Povl Riis’ Foundation, Bayer Health Care Funding, Augustinus Foundation, Munkholms Foundation, Tillotts Funding, Scientific Council at Herlev Hospital, Prof. Fagerhol Research Foundation, Aase & Einar Danielsen Foundation, Ole Trock‐Jansen & Hustrus Foundation, and European Crohn Colitis Organisation."
**Heida 2018**	NTR3759	Mixed IBD CD: IG: 39; CG: 42 UC: IG: 45; CG: 44	Remission	IG: 84 CG: 86	Immunomodulators: IG: 69; CG: 65 Aminosalicylates: IG: 57; CG: 52	NR	Emotional quotient: Low (≤ 89): IG: 5; CG: 5 Average (90–109): IG: 37; CG: 30 High (≥ 110): IG: 46; CG: 51 Missing: IG: 21; CG: 14	"PFvR, AH and AMK received funding for joint research projects from BÜHLMANN Laboratories and CisBio Bioassays. All other authors had no support from any organization for the submitted work, no financial relationships with any organizations that might have an interest in the submitted work in the previous 2 years, and no other relationships or activities that could appear to have influenced the submitted work."	"This work was supported by ZonMw Health Care Efficiency Research [grant number 837001001], Innovation Fund Dutch Insurance Companies [grant number B12‐204–2509], and NutsOhra Fund [grant number 1301‐002]. RKW is supported by the Netherlands Organization for Scientific Research [NWO] [grant number 016.136.308]. Reagents for the Quantum Blue® calprotectin point‐of‐care tests were an unrestricted donation by Bühlmann Laboratories AG. An unrestricted start‐up grant for the development of the web‐based programme IBD‐live was awarded by Ferring Pharmaceuticals BV."
**Hughes 2017**	NCT02707068	IBD	NR	IG: 32 CG: 31	NR	NR	NR	"None"	NR
**Ley 2020**	NR	UC	Remission	IG: 21 CG: 18	Lialda: IG: 7; CG: 11 Apriso: IG: 1; CG: 0 Balsalazide: IG: 5; CG: 4 Sulfasalazine: IG: 1; CG: 0 Asacol/delzicol: IG: 0; CG: 2 Asacol HD: IG: 7; CG: 1	NR	Employment: Student: IG: 3; CG: 5 Part‐time: IG: 1; CG: 1; Full‐time: IG: 16; CG: 11 Unemployed: IG: 1; CG: 1 Education: High school: IG: 4; CG: 0 College: IG: 3; CG: 5 Bachelors and above: IG: 14; CG: 13 Marital status: Single: IG: 9; CG: 7 Significant other/married: IG: 10; CG: 11 Divorced/widowed: IG: 2; CG: 0	"Freddy Caldera has received research support from Takeda Pharmaceuticals and Sanofi. He has been a consultant for Takeda and Celgene. All remaining authors report no proprietary interest in the products named in this article."	"This study was supported by research support from Takeda Pharmaceuticals."
**Malickova 2020**	NR	CD: IG: 44/94; CG: 19/37 UC: IG: 46/94; CG: 18/37	Remission	IG: 94 CG: 37	Corticosteroids: IG: 6; CG: 3 Azathioprine/6 ‐ mercaptopurine: IG: 30; CG: 17 Methotrexate: IG: 0; CG: 1 Mesalazine: IG: 49; CG: 20 Antibiotics: IG: 0; CG: 1	NR	Marital status: Single: IG: 29; CG: 14 Married/partner: IG: 55; CG: 20 Divorced/separated: IG: 6; CG: 3	NR	NR
**McCombie 2020**	ACTRN12615000342516	Mixed IBD CD: IG: 37; CG: 36 UC: IG: 13; CG: 14	Mean: remission	IG: 53 CG: 54	5‐ASA: IG: 20; CG: 20 Biologics: IG: 15; CG: 18 Thiopurine/methotrexate: IG: 37; CG: 27 None: IG: 2; CG: 3	NR	NR	"None"	"This work was supported by the Healthcare Otago Charitable Trust (no grant number) and The New Zealand Society of Gastroenterology Janssen Research Fellowship (no grant number) in 2015 and the gut health network, a research theme located at the Department of Medicine, University of Otago."
**Reich 2019**	NCT03241992	Mixed IBD CD: IG: 36; CG: 36 UC: IG: 28; CG: 27	Mean: remission	IG: 64 CG: 63	Mesalamine: IG:19; CG: 18 Immunomodulators: IG: 17; CG: 25 Biologics: IG 39; CG 40 Steroids: IG: 6; CG: 9	White: IG: 48; CG: 49 Black: IG: 8; CG: 7 Other: IG: 6; CG: 7	NR	"None"	"This project was funded by a generous gift from Aimee & Kleanthis Dendrinos and Robin & Andrew Davis."
**Siegel 2018**	NR	CD	NR	IG: 133 CG: 69	NR	NR	NR	NR	NR
**Stunkel 2012**	NR	IBD	Mild to moderate disease	Total: 90	NR	NR	NR	NR	NR
**Wang 2020**	NR	CD	Post‐operative CD Relapse: IG: 33; CG: 39 Remission: IG: 87; CG: 80 CG: Relapse 39, Remission 80.	IG: 120 CG: 119	NR	NR	NR	NR	"The project was funded by Nursing Project of Military Medical Science and Technology Youth Cultivation Plan, No. 19QNP077."

^a^ Numbers refer to number of participants unless otherwise specified. 5‐ASA: 5‐aminosalicylic acid; CD: Crohn's disease; CG: control group; IBD: inflammatory bowel disease; IC: indeterminate colitis IG: intervention group; IQR: interquartile range; n: number of participants; NR: not reported; UC: ulcerative colitis.

##### Setting

Six studies were conducted in the USA (Atreja 2018; Cross 2012; Cross 2019; Reich 2019; Siegel 2018; Stunkel 2012), one in Canada (Chauhan 2016), two in the UK (Akobeng 2015; Hughes 2017), three in Denmark (Ankersen 2019; Carlsen 2017a; Elkjaer 2010a), one in China (Wang 2020), one in Spain (Del Hoyo 2018), two in the Netherlands (de Jong 2017; Heida 2018), one in New Zealand (McCombie 2020), and one in Czechia (Malickova 2020). One study did not report the location (Ley 2020).

All studies were conducted in hospitals and tertiary centres. Nine studies were single‐centre RCTs (Akobeng 2015; Ankersen 2019; Atreja 2018; Carlsen 2017a; Chauhan 2016; Del Hoyo 2018; Malickova 2020; Reich 2019; Wang 2020), and nine were multicentre RCTs (Cross 2012; Cross 2019; de Jong 2017; Elkjaer 2010a; Heida 2018; Hughes 2017; McCombie 2020; Siegel 2018; Stunkel 2012). One study provided no information in this regard (Ley 2020).

One study was a cluster‐RCT (Siegel 2018).

##### Participants

Participant age ranged from eight years (Akobeng 2015) to 95 years (Elkjaer 2010a). Three studies examined paediatric populations (Akobeng 2015; Carlsen 2017a; Heida 2018). All other studies were in adults (aged 16 years and older).

Three studies examined exclusively UC populations (Cross 2012; Elkjaer 2010a; Ley 2020), two studies examined exclusively CD populations (Siegel 2018; Wang 2020), and the remaining studies examined a mix of IBD types.

Six studies included people with both active and inactive states of the disease (Carlsen 2017a; Cross 2012; Cross 2019; de Jong 2017; Del Hoyo 2018; Wang 2020), six studies included people with an inactive state of the disease (Akobeng 2015; Heida 2018; Ley 2020; Malickova 2020; McCombie 2020; Reich 2019), two studies included people with mild to moderate disease (Elkjaer 2010a; Stunkel 2012), one study included people in remission or with low disease activity (Ankersen 2019), and four studies did not report on the activity of the disease (Atreja 2018; Chauhan 2016; Hughes 2017; Siegel 2018).

Twelve studies reported trial registrations (Akobeng 2015; Ankersen 2019; Atreja 2018; Carlsen 2017a; Cross 2012; Cross 2019; de Jong 2017; Del Hoyo 2018; Heida 2018; Hughes 2017; McCombie 2020; Reich 2019).

##### Interventions

The studies evaluated the following interventions.

- Telephone consultations versus face‐to‐face consultations (Akobeng 2015)
- Mobile phone application disease monitoring versus self‐screening (Ankersen 2019)
- Mobile phone application disease monitoring versus sham education application (Atreja 2018, abstract only)
- Web‐based disease monitoring versus usual care (Carlsen 2017a)
- Telephone follow‐up visits versus clinic follow‐up visits (Chauhan 2016, abstract only)
- Web‐based care management portal versus usual care (Cross 2012)
- Web‐based care management portal weekly versus every other week versus usual care (Cross 2019)
- Web‐based care management portal versus usual care (de Jong 2017)
- Remote web‐based monitoring versus telephone‐based monitoring versus usual care (Del Hoyo 2018)
- Web‐based education and self‐treatment versus usual care (Elkjaer 2010a)
- Automated email alerts and web‐based telemonitoring versus usual care (Heida 2018)
- Cognitive behavioural therapy (CBT) self‐complete manual and telephone support versus usual care in waitlist (Hughes 2017, abstract only)
- Web‐based phone application for medication adherence versus sham application (Ley 2020)
- Web‐based application telemonitoring versus usual care (Malickova 2020; McCombie 2020)
- Web‐based IBD‐specific information and electronic reminders for medication adherence versus sham web‐based information unrelated to IBD (Reich 2019)
- Decision‐aid online programme for choice of combination therapy versus usual care (Siegel 2018, abstract only)
- Web‐based application disease monitoring versus usual care (Stunkel 2012, abstract only)
- Web‐based disease monitoring and medication adherence versus usual care (Wang 2020)

Cross 2019 and Del Hoyo 2018 were three‐arm studies. All other studies had two arms.

##### Outcomes

The length of the interventions ranged from eight weeks (Hughes 2017) to three years (Siegel 2018).

###### Primary outcomes

####### Disease activity

Eight studies reported disease activity as an outcome. Ankersen 2019 measured IBD activity using a colour‐coded system based on the Harvey Bradshaw Index (HBI) for CD participants, the Simple Clinical Colitis Activity Index (SCCAI) for participants with UC/indeterminate colitis, and Total Inflammatory Burden Score (TIBS) for both populations. Cross 2012 used the Seo Index to measure disease activity. Cross 2019 and McCombie 2020 used the HBI for CD participants and the SCCAI for UC participants. Malickova 2020 used the HBI for CD participants and the partial Mayo score for UC participants. Del Hoyo 2018 measured disease activity using faecal calprotectin (FC) levels, but provided no details in the report. Chauhan 2016 and Carlsen 2017a stated that disease activity was an outcome but provided no data.

####### Flare‐ups or relapse

Ten studies measured flare‐ups or relapses. Seven studies reported the number of relapses in each intervention group over the study period (Akobeng 2015; Ankersen 2019; Cross 2012; Cross 2019; Del Hoyo 2018; Heida 2018; McCombie 2020). de Jong 2017 and Elkjaer 2010a reported mean number of flare‐ups during the study as continuous data. Malickova 2020 reported relapses that needed hospitalisation.

####### Quality of life

Thirteen studies reported QoL (Akobeng 2015; Ankersen 2019; Atreja 2018; Chauhan 2016; Cross 2012; Cross 2019; de Jong 2017; Del Hoyo 2018; Elkjaer 2010a; Heida 2018; McCombie 2020; Reich 2019; Stunkel 2012). Four studies used the Inflammatory Bowel Disease Questionnaire (IBDQ; Cross 2012; Cross 2019; McCombie 2020; Stunkel 2012). Five studies used the Short Inflammatory Bowel Disease Questionnaire (SIBDQ; Ankersen 2019; Atreja 2018; de Jong 2017; Elkjaer 2010a; Reich 2019). Akobeng 2015 and Heida 2018 used the IMPACT questionnaire. Del Hoyo 2018 used the IBDQ‐9, the EuroQol five‐dimension questionnaire (EQ‐5D), and Visual Analogue Scales (VAS). Carlsen 2017a and Chauhan 2016) did not report the method used to measure QoL.

###### Secondary outcomes

####### Number of episodes of accessing healthcare

Nine studies reported the number of episodes of accessing healthcare (Akobeng 2015; Carlsen 2017a; Cross 2019; de Jong 2017; Del Hoyo 2018; Elkjaer 2010a; Heida 2018; Malickova 2020; McCombie 2020). Akobeng 2015 reported the number of participants in each group that had one or more hospital admissions. Carlsen 2017a reported total numbers of outpatient visits, on‐demand outpatient visits, acute hospitalisations, planned outpatient visits, and contacts in total. Cross 2019 reported total encounters, IBD‐related hospitalisations, non‐IBD‐related hospitalisations, non‐invasive diagnostic tests, electronic encounters, and telephone encounters (per 100 participants per year). de Jong 2017 reported the mean number of hospital admissions and outpatient visits. Del Hoyo 2018 reported the number of outpatient visits. Elkjaer 2010a reported the number of acute and routine hospital visits per group. Heida 2018 reported face‐to‐face encounters with healthcare providers. Malickova 2020 reported the mean number of visits to doctors and IBD nurses and the mean number of hospitalisations per participant. McCombie 2020 reported the mean number of gastroenterologist appointments, surgical appointments, IBD hospitalisations, and nights in hospital.

####### Medication adherence

Seven studies measured medication adherence (Ankersen 2019; Carlsen 2017a; Cross 2012; de Jong 2017; Del Hoyo 2018; Ley 2020; Wang 2020). Ankersen 2019 and Carlsen 2017a used self‐assessment questionnaires with the Medication Adherence Report Scale (MARS). Cross 2012, de Jong 2017, and Wang 2020 used the Morisky Medication Adherence Scale (MMAS). Del Hoyo 2018 used the Morisky‐Green Index. Ley 2020 used the Medication Possession Ratio (MPR).

####### Participant engagement

Eleven studies studied participant engagement (Ankersen 2019; Carlsen 2017a; Cross 2019; Del Hoyo 2018; Elkjaer 2010a; Heida 2018; Hughes 2017; Malickova 2020; McCombie 2020; Reich 2019; Stunkel 2012). Ankersen 2019 reported participant satisfaction. Carlsen 2017a reported adherence as the number of entries in their web programme by participants. Cross 2019 defined adherence as 80% or more completion of self‐assessments. Del Hoyo 2018 measured adherence as compliance with more than 80% of checkups. Elkjaer 2010a assessed compliance via a compliance questionnaire. Heida 2018 reported compliance as more than 80% response to alerts. Hughes 2017 reported the percentage of participants completing at least one telephone session. McCombie 2020 reported the results of two system usability scales (SUS). Malickova 2020 reported non‐compliance numbers without any further details. Reich 2019 reported the percentage of participants logging into their web application. Stunkel 2012 reported feedback from participants without providing further details.

####### Rate of attendance or engagement with any or all elements of the intervention

Only three studies reported attendance/engagement as number of planned appointments/interactions attended (Akobeng 2015; Carlsen 2017a; McCombie 2020). Akobeng 2015 reported the median number of consultations scheduled by the hospital and the median number of consultations attended per person. Carlsen 2017a reported the number of planned outpatient visits. McCombie 2020 reported the number of people completing FC readings.

####### Rate of attendance of interactions with healthcare professionals

Only Akobeng 2015 and Del Hoyo 2018) reported rate of interactions attended. Akobeng 2015 reported the percentage of participants who had at least one consultation allocated. Del Hoyo 2018 reported percentage of outpatient visits.

####### Costs or cost/time‐effectiveness

Eight studies reported costs or cost/time‐effectiveness (Akobeng 2015; Carlsen 2017a; Chauhan 2016; de Jong 2017; Del Hoyo 2018; Elkjaer 2010a; Heida 2018; Malickova 2020). Akobeng 2015 estimated costs to the UK National Health Service (NHS). Carlsen 2017a estimated economic gains. Chauhan 2016 reported the average parking and travel costs with an average loss of income. de Jong 2017 stated mean annual direct costs and mean annual savings. Del Hoyo 2018 used cost and effect data to obtain cost‐effectiveness and cost‐utility, but provided no specific details. Elkjaer 2010a converted the number of medications plus professional visits into financial savings for the department. Heida 2018 reported mean annual cost‐saving. Malickova 2020 estimated the reduction on average annual costs between the groups.

###### Qualitative synthesis

####### Type of Telehealth

Table 7 and Table 8 provide details of the contents of each intervention.

**2:** Intervention details

**Study ID**	**Intervention description**	**Type of telehealth**	**Control intervention description**	**Type of control intervention**	**Intervention length**	**Is the education part of a package of measures (e.g. diagnostic tools, etc.)?**	**Outcome measurement points**	**Follow‐up measurement points**
**Akobeng 2015**	"A call from the gastroenterology doctor at the time of their appointment. The consulting doctor contacted the patient and parents via a telephone number (home or mobile) that the parents and patient had previously supplied as the number they would like to be contacted on."	Telephone consultations	Routine appointments in hospital as usual	Usual care	24 weeks	No	6, 12, 18, 24 months	None after end of study
**Ankersen 2019**	"If patients experienced a recurrence of disease visualized on constant care web application (web‐app), they were instructed to contact the electronic care (eCare) personnel by phone or via the patient's personal web‐wall, for an early consultation to assess the need of individualized treatment adjustment or diagnostic investigation. Daily web ward rounds were performed by the eCare nurses in close collaboration with a medical doctor."	Mobile phone application disease monitoring	Patients allocated to the CG were instructed in how to screen themselves every 3 months.	Self‐screening	12 months	No	12 months	None after end of study
**Atreja 2018**	"HealthPROMISE app: Patients track their Quality Of Life and symptoms every 2 weeks, providers can use the visual data to provide better care."	Mobile phone application disease monitoring	Patient education application, no further details provided	Patient education application	104 weeks	NR	Day 495, day 575	None after end of study
**Carlsen 2017**	"Electronic traffic light system, which guides the scheduling of infliximab treatment at intervals of 4 to 12 weeks. The traffic light system is based on patient‐registered symptom scores and measures of fecal calprotectin (FC), combined into a total inflammation burden score (TIBS). The repeatedly measured TIBS form a curve on a traffic light graph system consisting of the colors green, yellow, and red. Depending on the color, patients are advised regarding the timing of their next IFX treatment."	Web‐based disease monitoring	Hospital's IBD care guidelines (national pediatric IBD standard care in Denmark), with outpatient visits every 3rd month, including blood samples and FC.	Usual care	2 years	NR	End of study	None after end of study
**Chauhan 2016**	Telephone follow‐up visits by an IBD nurse practitioner	Telephone follow‐ups	Clinic follow‐up visit by an IBD nurse practitioner	Usual care	6 months	NR	6 months	None after end of study
**Cross 2012**	"Mobile phone for participants and a decision support server and website for staff and providers. The web system send texts to participants grading their IBD symptoms and collected data from each testing session. Educational tips were also sent via text. The provider could individualise alerts and action plans for each participant. If pre‐determined criteria were met the nurse reviewed and if necessary management changes were made. Medication changes were also updated and communicated to the patient."	Web‐based care management portal	"Comprehensive assessment, a guideline‐concordant therapy plan, scheduled and as‐needed clinic visits, scheduled and as‐needed telephone calls, administration of educational fact sheets about disease‐specific topics. Administration of educational materials was not standardised and was at the discretion of the provider."	Usual care	12 months	Disease‐specific education provided by C&C Foundation of America	6 months, 12 months	None after end of study
**Cross 2019**	"Mobile phone for participants and website for providers. The web system sends texts to participants to grade their IBD symptoms. The website provides an interface for staff and providers for participants profiles and collected data from each testing session. The provider can individualize alerts and action plans for each participant. If pre‐determined criteria were met after testing, simultaneous action plans and email alerts were sent to the participant and nurse respectively. The nurse reviewed the information and if necessary consulted the provider for management changes. Medication changes were updated in the participant profile and communicated to the participant."	Web‐based care management portal	"The standard of care for participants in this study is modeled after the standard of care at all three study sites. Comprehensive assessment, a guideline concordant therapy plan scheduled and as needed clinic visits, scheduled and as needed telephone calls, and administration of educational fact sheets about disease‐specific topics when appropriate."	Usual care	12 months	"Educational curriculum: education tips either twice weekly (IG1) or every week (IG2). Educational materials for CG administration was not standardized and was at the discretion of the treating provider."	6 and 12 months	None after end of study
**De Jong 2017**	"MyIBDcoach is a secured webpage with an HTML application for tablet or smartphone. The system includes monthly monitoring modules, as well as intensified monitoring modules, outpatient visit modules, e‐learning modules, a personal care plan, and an administrator page used by the health‐care provider. When parameters recorded by the monitoring modules exceeded predefined thresholds, the safety and continuity of care were ensured by the creation of alerts (red flags) on the administrator page of each local hospital. If an alert was received, a health‐care provider on the local team contacted the patient for further assessment within two working days. Visits to the outpatient clinic were based on the nature and severity of the clinical complaints. At any time, patients were able to communicate easily with their health‐care provider by sending a message to the health‐care providers’ administration office."	Web‐based care management portal	"Patients in the standard care group continued their routine follow‐up visits following the local protocol, with an opportunity to schedule an extra visit if symptoms relapsed."	Usual care	12 months	NR	12 months	None after end of study
**Del Hoyo 2018**	IG1: "Follow‐up and monitoring were performed telematically using the integrated platform for management of chronically ill patients (NOMHADCHRONIC app). Patients connected to the platform via the Internet using a computer or an app on a mobile phone or tablet had to self‐complete questionnaires. In addition, they received advice, reminders, educational material about their disease, and information on prevention. This information was received by the case managers and filtered using an intelligent prioritization system with generation of alerts and push notifications according to an integrated intervention protocol" IG2: "The G_NT patients were asked about their health through telephone calls by the nursing staff in the IBD Unit. Authors performed telephone assessment periodically by using structured interviews to evaluate health status, and clinical activity was self‐recorded at home. The interventions depended on the results of the interview and changes in the medication or follow‐up schedule established by nurses with the support of medical staff, according to the alerts and action plans designed in the intervention protocol. Furthermore, they provided these patients with all educational elements made available to the other 2 groups"	IG1: remote web‐based monitoring IG2: nurse‐assisted telephone care	"The CG patients received the normal care provided in the IBD Unit (Outpatient Clinic) for patients with moderately to highly complex IBD, based on national and European clinical guidelines. Treatment was adjusted according to the evolution of disease activity and medication adherence, which was measured using specific indexes and biological markers used to report the study outcomes during office visits or telephone calls. This care was complemented by ad hoc hospital care in case of flareups or if the patient’s health deteriorated for any reason. Ad hoc intensive care was maintained until the patient’s condition stabilized, at which point he or she returned to follow‐up based on standard care in the Unit."	Usual care	24 weeks	NR	12 and 24 weeks	None after end of study
**Elkjaer 2010**	"Patients received a remote education session on IBD and training on the web‐based programme on how to recognise relapses and start treatment guided by the programme. In case of relapse, patients were requested to log on daily and complete the disease activity score (SCCAI) until they entered the green zone. Patients should then log on once a week for a total of 4 weeks after the initiation of relapse. Once remission was achieved patients had to use the program once a month until the next relapse occurred."	Web‐based education and self‐treatment	"Patients in the control group continued the conventional treatment and follow‐up in the IBD out‐patient clinic."	Usual care	12 months	IG: web platform, education from staff members	End of study	None after end of study
**Heida 2018**	"Participants received automated email alerts to fill in a symptom score and to send in a stool sample. The results of both the symptom score and the calprotectin stool test were uploaded on the IBD‐live website and cumulated in a colour‐coded disease flare risk stratification that was visible to the individual participant and the local IBD team. This resulted in an individual prediction for flare with associated treatment advice and test interval."	Automated email alerts, and web‐based telemonitoring	Regular checks in the consultation room as before the trial	Usual care	52 weeks	Yes, FC samples – diagnostic measure	End of study	None after end of study
**Hughes 2017**	"Quality Of LIfe Tool for IBD (QOLITI). The cognitive‐behavioural therapy (CBT)‐inspired manual contains several chapters each of which addresses a different topic with information, guidance in setting goals for behaviour change and accompanying tasks to aid implementation which is completed at home in the participant's own time. Key themes are likely to include symptom management, dealing with social implications of the disease and interacting effectively with healthcare professionals among others. 3 x 30 minutes of telephone support by a trained healthcare professional along with the manual were included. Telephone calls occurred at two, four and six weeks post‐randomisation."	CBT self‐complete manual and telephone consultations	Waitlist control group waits until after the study finishes to receive the same manual, but without telephone support sessions	Usual care (waitlist)	8 weeks	Yes, educational manual	End of study	None after end of study
**Ley 2020**	Adherence iPhone application that included medication reminders	Web‐based phone application for medication adherence	Sham application installed that included educational materials and the capability of recording medication intake, without medication reminders	Sham application	NR	No	End of study	NR
**Malickova 2020**	"Patients were telemonitored and connected with their doctors and IBD nurses through an IBD Assistant application. They received email reminders at regular intervals to fill in standard electronic assessments. In case of deterioration, they had an emergency questionnaire that advised on contacting a doctor. All communication with the doctor was made primarily through the IBD Assistant web application, personal visits were carried out only after a previous recommendation via the IBD Assistant application. FC was measured at least 4 times/12months with at home CalpoSmart system."	Web‐based application telemonitoring	"There were usual check‐ups every 3 months in outpatient clinics with their gastroenterologists, during which the patients were examined clinically and laboratory. In case of any difficulties, patients had an unscheduled acute consultation, or were visited by a doctor on the basis of unfavorable examination results."	Usual care	12 months	Yes, FC samples – diagnostic measure	End of study	None after end of study
**McCombie 2020**	"IBDsmart is an app that allows inflammatory bowel disease (IBD) patients to regularly fill in symptom scores and get them sent to their doctor. It is used by the patients by logging in and filling out a questionnaire. When they fill out the questionnaire, a score is produced which indicates the severity of the disease. This way long term trends of symptom scores are kept on the smartphone and the healthcare team can be contacted immediately via the app in cases where disease severity is high. IBDoc is an app that allows IBD patients to measure their faecal calprotectin levels and get their results sent to their doctor. The way the app works is the participant provides a stool sample which is analysed using a medical device which produces an output that can be read via the camera by an app. The calprotectin app communicates with the IBD app which produces a faecal calprotectin score which is high, medium, or low; the level indicates how much physical disease activity is occurring in the patient. These results can also be sent to the healthcare professional team."	Web‐based telemonitoring	"Usual outpatient treatment. The usual treatment group will not have access to the smartphone apps. Usual outpatient treatment, for the purposes of this study, entails the patient seeing their treating gastroenterologist as they usually would."	Usual care	12 months	Yes, FC samples – diagnostic measure	3, 6, 9, 12 months	None
**Reich 2019**	"Patients received information via an application about IBD every 2 weeks along with reminders to take their medications. They also received a reminder about getting vaccinated for influenza and pneumococcal pneumonia at 2 weeks, and 3 months after enrollment."	Web‐based IBD‐specific information and electronic reminders for medication adherence	Participants were sent generic messages unrelated to IBD.	Sham web‐based information unrelated to IBD	6 months	Yes, educational information about IBD sent via messages	End of study	None
**Siegel 2018**	"A decision aid including an online program reviewing benefits and risks of treatment options combined with a personalised risk prediction tool for Crohn’s disease."	Decision‐aid online programme for choice of combination therapy	Standard of care	Usual care	3 years	Yes, benefits and risks of treatment review	End of study	NR
**Stunkel 2012**	"Subjects downloaded and used an application daily to record symptoms, track pain, stress levels, frequency and quality of bowel movements."	Web‐based application disease monitoring	The control group was educated about websites providing information on IBD.	Usual care	38 weeks	No	End of intervention (varied 8–38 weeks)	IG: 104 days CG: 87 days
**Wang 2020**	"Nurse‐led web‐based follow‐up program for disease monitoring, patient medication reminders, medication education and nurse‐caregiver‐patient communication"	Web‐based disease monitoring and medication adherence	"The patients in the control group received regular health education and guidance on drugs by designated nurses during their in‐patient stay. They were handed a brochure with drug guidance upon discharge. The content of the brochure included basic knowledge of drugs, drug usage and effects, how to deal with common problems, and how to attend follow‐ups in outpatient clinic. Every two months, doctors followed‐up guidance by telephone."	Usual care	6 months	Yes. Disease monitoring, patient reminders, patient education, nursing‐patient group chat for questions.	End of months 1, 2, 4, 6	NR

CG: control group; FC: faecal calprotectin; IBD: inflammatory bowel disease; IG: intervention group; NR: not reported.

**3:** Telehealth details

**Study ID**	**Time to response**	**Staff and programmes delivering the intervention**	**Resources required for the intervention and who provided them**	**Access issues as reported in studies (e.g. disabilities, financial issues)**	**Data security**
**Akobeng 2015**	NR	IG: gastroenterologist CG: gastroenterologist	Gastroenterologist provided by the hospital; telephone access	None apart from lack of access to a telephone	NR
**Ankersen 2019**	NR	IG: eCare Nurse CG: eCare Nurse	Smartphone (participants' own); eCare nurses + doctors for the web rounds	NR	NR
**Atreja 2018**	NR	NR	Smartphone, access to the Internet (participants' own)	NR	NR
**Carlsen 2017**	NR	IG: programme CG: hospital staff	Smartphone, access to internet (participants' own). Training by principal investigator	NR	NR
**Chauhan 2016**	NR	IBD nurse practitioner	Telephone (participants' own)	NR	NR
**Cross 2012**	NR	Home telemanagement/ standard care staff	"[...] for participants without an active telephone line, a cell phone is provided to transmit self‐testing results over a secure wireless network."	NR	Data transmitted from the participant's home were deidentified and encrypted.
**Cross 2019**	IG: "Results are available immediately after self‐test completion. Clinical care issues that require immediate attention are directed to the provider's office or on call service at each site. Providers are available to study nurse coordinators daily to provide guidance for management changes. CG: face‐to‐face appointments"	IG: web portal, nursing staff, doctors CG: doctors, nursing staff	IG: mobile phone (participants' own), electronic weight scale	NR	NR
**De Jong 2017**	IG: "If an alert was received, a health‐care provider on the local team contacted the patient for further assessment within two working days."	IG: website CG: standard hospital care	IG: computer/tablet/smartphone and internet access (participants' own), administration office	NR	NR
**Del Hoyo 2018**	NR	IG: the platform, specialised medical staff and nurses Telephone IG: nursing staff CG: hospital staff	Telephone, mobile phone, internet access (participants' own)	NR	"TECCU Web platform protects the confidentiality of health data. The access to patient station and to work station requires a personal password only known by the patient and healthcare providers, respectively. Moreover, healthcare providers register patients in the platform with a generic name and a code only identifiable by investigators. Finally, to avoid data correlation by a nonauthorized person, data included in the Web platform are not connected to other hospital information systems. Thus, only case managers and health professionals can see all the clinical history separately."
**Elkjaer 2010**	NR	IG: web platform, education from staff members CG: staff members (regular care)	Computer (participants' own)	NR	NR
**Heida 2018**	NR	IG: programme CG: specialists not defined	Access to telephone, internet, and email (participants' own)	Participants required to have access to telephone, internet, and email, and good knowledge of Dutch	NR
**Hughes 2017**	NR	IG: telephone calls + self‐management CG: self‐management	Manuals, task books, telephones and personnel, provider not mentioned	"Suicidal patients will be directly referred to liaison psychiatry or their GP and will not be able to access the study as the intensity of the manual intervention is within the low‐moderate range."	NR
**Ley 2020**	NR	IG/CG: iPhone app	iPhone, provider not mentioned	NR	NR
**Malickova 2020**	NR	IG: Web IBD Assistant App CG: gastroenterologist	PC, tablet, or smartphone, and working email address (participants' own)	Excluded: no smartphone/PC, language barrier, no email, no wifi	NR
**McCombie 2020**	NR	IG: smartphone app + gastroenterologist CG: gastroenterologist	IG: smartphone (can be borrowed). 17/50 participants used a borrowed smartphone.	Excluded: people unable to provide written consent	NR
**Reich 2019**	NR	IG/CG: Electronic Health Record (EHR) patient portal (EPIC's Mychart)	Computer with internet (participants' own)	Excluded: non‐English speaking, cognitive impairment that would impair participation, no computer with internet.	NR
**Siegel 2018**	NR	IG: online programme CG: NR	NR	NR	NR
**Stunkel 2012**	NR	IG: smartphone app CG: self‐education using websites	Smartphones (participants' own)	"Patients with Blackberry® smart phones were excluded as the app was not fully optimized for this device."	NR
**Wang 2020**	NR	IG: mobile app CG: nurses	Mobile phones, provider not mentioned	People "not able to use the app" were excluded from the study	NR

CG: control group; IBD: inflammatory bowel disease; IG: intervention group; NR: not reported.

Three studies compared telephone consultations to usual care (Akobeng 2015; Chauhan 2016; Hughes 2017). Two studies compared web‐based disease monitoring programmes to usual care (Carlsen 2017a; McCombie 2020). Four studies evaluated web‐based care management programmes versus usual care (Cross 2012; Cross 2019; de Jong 2017; Siegel 2018). Two studies evaluated web‐based monitoring together with automated email alerts versus usual care (Heida 2018; Malickova 2020). Ankersen 2019 investigated a mobile phone application for disease monitoring versus self‐screening. Atreja 2018 compared a mobile phone application for disease monitoring to a patient education application. Elkjaer 2010a compared web‐based online education and self‐treatment to usual care. Ley 2020 compared a web‐based phone application for medication adherence to a sham application (containing educational materials and capability to record medication intake). Reich 2019 evaluated a web‐based application with IBD‐specific information and reminders for medication adherence versus a sham application. Stunkel 2012 evaluated a web‐based application for disease monitoring versus websites with information regarding IBD. Wang 2020 evaluated nurse‐led web‐based disease monitoring and medication adherence application versus usual care. Del Hoyo 2018 evaluated remote web‐based monitoring versus nurse‐assisted telephone care versus usual care.

####### Other components of the intervention

Seven studies reported educational components as part of the telehealth intervention (Cross 2012; Cross 2019; Elkjaer 2010a; Hughes 2017; Reich 2019; Siegel 2018; Wang 2020). Table 7 provides further details. Three studies measured FC as part of the diagnostic assessment (Heida 2018; Malickova 2020; McCombie 2020).

####### Length of intervention, resources, access issues, data security

Length of the intervention varied between eight weeks (Heida 2018) and three years (Siegel 2018). For details, see Table 7.

Necessary resources were a mobile phone in 16 studies (Akobeng 2015; Ankersen 2019; Atreja 2018; Carlsen 2017a; Chauhan 2016; Cross 2012; Cross 2019; de Jong 2017; Del Hoyo 2018; Heida 2018; Hughes 2017; Ley 2020; Malickova 2020; McCombie 2020; Stunkel 2012; Wang 2020), a computer in four studies (de Jong 2017; Elkjaer 2010a; Malickova 2020; Reich 2019), and internet connection in seven studies (Atreja 2018; Carlsen 2017a; de Jong 2017; Del Hoyo 2018; Heida 2018; Malickova 2020; Reich 2019). Cross 2019 and McCombie 2020 stated that they provided devices to their participants. Cross 2019 required participants to have an electronic weight scale. Table 8 provides further details.

Not having access to a smartphone, computer, or internet was explicitly reported as an access issue in four studies (Akobeng 2015; Heida 2018; Malickova 2020; Reich 2019). Three studies reported language barrier as an access issue (Heida 2018; Malickova 2020; Reich 2019). Wang 2020 excluded people who were unable to use the web application. Stunkel 2012 excluded people with Blackberry phones. Reich 2019 excluded those with a degree of cognitive impairment that would impair participation. McCombie 2020 excluded people who were unable to provide written consent. Hughes 2017 excluded people with suicidal ideations. Table 8 provides further details.

Two studies commented on data security: Cross 2012 mentioned that the data transmitted from participants' homes was de‐identified and encrypted, and Del Hoyo 2018 mentioned confidentiality measures to secure the data provided. Table 8 provides further details.

##### Funding sources and conflicts of interest

Fourteen studies reported their sources of funding (Akobeng 2015; Ankersen 2019; Atreja 2018; Carlsen 2017a; Cross 2012; Cross 2019; de Jong 2017; Del Hoyo 2018; Elkjaer 2010a; Heida 2018; Ley 2020; McCombie 2020; Reich 2019; Wang 2020). Four studies were funded via government grants (Akobeng 2015; Atreja 2018; Cross 2012; Cross 2019), nine studies by private sources (Ankersen 2019; Carlsen 2017a; de Jong 2017; Del Hoyo 2018; Heida 2018; Elkjaer 2010a; Ley 2020; Reich 2019; Wang 2020), and one study by a charity and non‐profit research association (McCombie 2020).

Five studies provided no information regarding their source of funding (Chauhan 2016; Hughes 2017; Malickova 2020; Siegel 2018; Stunkel 2012).

Twelve studies made conflicts of interest declarations (Akobeng 2015; Ankersen 2019; Carlsen 2017a; Cross 2019; de Jong 2017; Del Hoyo 2018; Elkjaer 2010a; Heida 2018; Hughes 2017; Ley 2020; McCombie 2020; Reich 2019). Five studies declared no conflicts of interest (Carlsen 2017a; Cross 2019; Hughes 2017; McCombie 2020; Reich 2019), four studies declared that several authors received grants or non‐financial support from private providers (Ankersen 2019; de Jong 2017; Heida 2018; Ley 2020), one study reported receiving research grants during the conduct of the study (Akobeng 2015), and two studies declared that several authors had connections to healthcare companies unrelated to the study (Del Hoyo 2018; Elkjaer 2010a)

Seven studies provided no conflicts of interest declarations (Atreja 2018; Chauhan 2016; Cross 2012; Malickova 2020; Siegel 2018; Stunkel 2012; Wang 2020).

#### Excluded studies

We excluded 27 studies (42 records; see Characteristics of excluded studies). The main reason for exclusion was wrong intervention in 14 studies (Ankersen 2017; Carlsen 2017b; Elkjaer 2010b; Jambaulikar 2015; NCT01852097; NCT02265588; NCT02707068; NCT03486158; NCT03695783; Oser 2018; RBR‐79dn4k; Sutton 2019; Tripp 2017; Zhang 2020), wrong population in one study (NCT00310362), and wrong study design in 12 studies (Camba 2013; Creed 2019; Del Hoyo 2021; Gray 2020; Greenley 2015; Krier 2011; Mastronardi 2020; Miloh 2017; Moss 2010; NCT04151420; NCT04165265; Snoei 2009).

#### Studies awaiting classification

There are nine studies (10 records) awaiting classification (Bonnaud 2021; Hommel 2015; NCT02085083; NCT02694042; NCT03059186; NCT03186872; NCT04754620; NTR2892; NTR4648).

#### Ongoing studies

We identified nine ongoing studies (10 records; ACTRN12617000389303; IRCT2020061304775; NCT03985800; NCT04207008; NCT04388865; NCT04653259; NCT04861597; Norton 2021; RBR‐7t8fv7).

### Risk of bias in included studies

For a graphical presentation of the results of our risk of bias assessment, see Figure 2 and Figure 3. Further details can be found in the risk of bias tables (in the Characteristics of included studies table).

**2:**
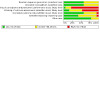
Risk of bias graph: review authors' judgements about each risk of bias item presented as percentages across all included studies.

**3:**
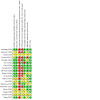
Risk of bias summary: review authors' judgements about each risk of bias item for each included study.

#### Allocation

Ten studies clearly described random sequence generation and allocation concealment, so we judged them at low risk of selection bias in both domains (Akobeng 2015; Chauhan 2016; Cross 2012; Cross 2019; de Jong 2017Del Hoyo 2018; Elkjaer 2010a; Heida 2018; McCombie 2020; Wang 2020). Seven studies provided insufficient information on random sequence generation and allocation concealment, so we judged them at unclear risk of selection bias (Ankersen 2019; Atreja 2018; Hughes 2017; Ley 2020; Reich 2019; Siegel 2018; Stunkel 2012). We considered Carlsen 2017a at unclear risk in relation to random sequence generation and low risk for allocation concealment (overall unclear risk of selection bias), and we judged Malickova 2020 at low risk regarding random sequence generation and unclear risk for allocation concealment (overall low risk of selection bias).

#### Blinding

Due to the nature of the interventions, 15 studies could not blind participants and personnel and so were at high risk of performance bias (Akobeng 2015; Ankersen 2019; Carlsen 2017a; Chauhan 2016; Cross 2012; Cross 2019; de Jong 2017; Del Hoyo 2018; Elkjaer 2010a; Heida 2018; Hughes 2017; Malickova 2020; McCombie 2020; Reich 2019; Wang 2020). Only Ley 2020 was at low risk of performance bias, and we judged three studies at unclear risk (Atreja 2018; Siegel 2018; Stunkel 2012).

We considered three studies at low risk of detection bias as they mentioned or confirmed blinding of outcomes assessors (Cross 2012; Cross 2019; Malickova 2020). Seven studies provided insufficient information for judgement (Atreja 2018; Hughes 2017; Ley 2020; Reich 2019; Siegel 2018; Stunkel 2012; Wang 2020), and nine studies were at high risk because they confirmed or stated that assessors were unblinded (Akobeng 2015; Ankersen 2019; Carlsen 2017a; Chauhan 2016; de Jong 2017; Del Hoyo 2018; Elkjaer 2010a; Heida 2018; McCombie 2020).

#### Incomplete outcome data

We considered eleven studies at low risk of attrition bias because they provided sufficient information to make a judgement (Akobeng 2015; Ankersen 2019; Carlsen 2017a; Chauhan 2016; Cross 2019; de Jong 2017; Del Hoyo 2018; Elkjaer 2010a; Malickova 2020; McCombie 2020; Wang 2020). The remaining seven studies were at unclear risk as they provided insufficient information to make a clear judgement (Atreja 2018; Heida 2018; Hughes 2017; Ley 2020; Reich 2019; Siegel 2018; Stunkel 2012). We rated one study at high risk of attrition bias (Cross 2012).

#### Selective reporting

We judged eight studies at low risk of reporting bias, as they reported all outcomes set out in their trial registrations (Akobeng 2015; Cross 2012; Cross 2019; de Jong 2017; Del Hoyo 2018; Heida 2018; McCombie 2020; Reich 2019). We considered one study at high risk, as the prioritisation of outcomes differed between the protocol and the published manuscript (Carlsen 2017a). The remaining studies provided insufficient information for judgement (Ankersen 2019; Atreja 2018; Chauhan 2016; Elkjaer 2010a; Hughes 2017; Ley 2020; Malickova 2020; Siegel 2018; Stunkel 2012; Wang 2020).

#### Other potential sources of bias

We rated fifteen studies at low risk of other potential sources of bias (Akobeng 2015; Ankersen 2019; Carlsen 2017a; Chauhan 2016; Cross 2012; Cross 2019; de Jong 2017; Del Hoyo 2018; Heida 2018; Hughes 2017; Ley 2020; McCombie 2020; Reich 2019; Siegel 2018; Wang 2020). Four studies provided insufficient information for judgement (Atreja 2018; Elkjaer 2010a; Malickova 2020; Stunkel 2012).

### Effects of interventions

See: Table 1; Table 2; Table 3; Table 4; Table 5

#### 1. Web‐based disease monitoring versus usual care

Twelve studies evaluated web‐based disease monitoring versus usual care (Carlsen 2017a; Cross 2012; Cross 2019; de Jong 2017; Del Hoyo 2018; Elkjaer 2010a; Heida 2018; Malickova 2020; McCombie 2020; Siegel 2018; Stunkel 2012; Wang 2020). Two of these studies were in paediatric populations (Carlsen 2017a; Heida 2018).

##### Primary outcomes

Table 1 presents the effect measures (where calculated) and GRADE judgements for the primary outcomes.

###### Disease activity

Five studies reported disease activity (Cross 2012; Cross 2019; Del Hoyo 2018; Malickova 2020; McCombie 2020). 

Three studies provided data that we could use for meta‐analysis (Cross 2012; Cross 2019; McCombie 2020). All three studies enrolled only adults. Web‐based disease monitoring (n = 254) is probably equivalent to usual care (n = 174) in reducing disease activity in adults with IBD (SMD 0.09, 95% CI −0.11 to 0.29; Analysis 1.1). The certainty of the evidence is moderate, downgraded for risk of bias mainly due to lack of blinding. Subgroup comparison showed similar disease activity in the UC and CD groups. A fixed‐effect sensitivity analysis showed no difference in the results (Analysis 1.2).

Del Hoyo 2018 and Malickova 2020 did not provide suitable data for meta‐analysis. Del Hoyo 2018 measured disease activity only by proxy (FC levels) and reported no variance measure. At 24 weeks, the median FC level for clinical activity was 137 μg/g in the web‐based group and 230 μg/g in the control. Malickova 2020 reported HBI mean scores of 3.48 in the web‐based group and 2.71 in the control, and Partial Mayo mean scores of 2.71 in the web‐based group and 2.57 in the control. We were unable to draw any conclusions from these results. We downgraded the certainty of the evidence to very low for imprecision (very low participant numbers) and for risk of bias concerns (lack of blinding, selective reporting, and other bias).

###### Flare‐ups or relapse

Seven studies reported flare‐ups or relapse with suitable data for meta‐analysis (Cross 2012; Cross 2019; de Jong 2017; Del Hoyo 2018; Elkjaer 2010a; Heida 2018; McCombie 2020). Six studies enrolled adults (Cross 2012; Cross 2019; Del Hoyo 2018; de Jong 2017; Elkjaer 2010a; McCombie 2020), and one study enrolled children (Heida 2018).

Web‐based disease monitoring (n = 207/496) is probably equivalent to usual care (n = 150/372) for the occurrence of flare‐ups or relapses in adults with IBD (RR 1.09, 95% CI 0.93 to 1.27; 5 studies; Analysis 1.3). We downgraded the certainty of the evidence to moderate for risk of bias (lack of blinding, reporting bias, and other bias). Subgroup comparison showed no major differences between the mixed IBD, UC, and CD groups. A fixed‐effect sensitivity analysis showed no difference in the results (Analysis 1.4).

de Jong 2017 provided continuous data for flare‐ups or relapses. Web‐based disease monitoring (n = 465) is probably equivalent to usual care (n = 444) for the occurrence of flare‐ups or relapses in adults with CD (MD 0.00 events, 95% CI −0.06 to 0.06; Analysis 1.5). We downgraded the certainty of the evidence to moderate for lack of blinding. 

Heida 2018 evaluated a paediatric population of mixed CD and UC patients. Web‐based disease monitoring (n = 28/84) may be equivalent to usual care (n = 29/86) for the occurrence of flare‐ups or relapses in children with IBD (RR 0.99, 95% CI 0.65 to 1.51; Analysis 1.6). We downgraded the certainty of the evidence to low for imprecision (low participant numbers) and risk of bias concerns (lack of blinding and imbalance in number of participants reaching end of study between the two groups). 

Table 9 provides further details.

**4:** Primary outcome data

**Study ID**	**1a. Disease activity at study end**	**1b. Flare‐ups/relapses measured clinically/endoscopically/histologically (n, unless otherwise specified)**	**1c. Quality of life**
**Akobeng 2015**	NR	Disease relapses over 24 months IG: 1/44 CG: 4/42	Median IMPACT QoL at 12 months: IG (n = 36): median 113 points (IQR 105–125); calculated SD 14.8 CG (n = 31): median 106 points (IQR 95–116); calculated SD 15.5 Mean IMPACT QoL: IG: mean 108.2 points (95% CI 101.6–114.7) CG: mean 102.5 points (95% CI 96.5–108.4)
**Ankersen 2019**	"Two assessors classified disease activity as (1) Chronic continuous course, red throughout 1 year; (2) Chronic continuous course, yellow throughout 1 year; (3) Chronic continuous course, red and yellow throughout1 year; (4) Continuous remission course, green throughout 1 year; (5) Intermittent course; green, yellow and red throughout 1 year; and (6) Intermittent course; green with a single relapse (yellow or red) throughout 1 year." Mean % over 1 year: SCCAI scores: IG (n = 37) green/yellow/red: 82%/15%/3% CG (n = 35) green/yellow/red: 87%/10%/3% HBI scores: IG (n = 6) green/yellow/red: 72%/28%/0% CG (n = 9) green/yellow/red: 66%/34%/0% TIBS scores: IG (n = 43) green/yellow/red: 60%/26%/14% CG (n = 39) green/yellow/red: 61%/22%/16%	Study authors stated they "analysed the number of relapses (FC and SCCAI) in each intervention group based on 83 (99%) and 70 (97%) patients respectively"; however, the numbers randomised were 50 and 52. "Moderate" and "Severe" relapses combined: IG (FC): 22 CG (FC): 17 IG (SCCAI): 14 CG (SCCAI): 9	Short IBDQ change in QoL: IG: mean 0.56 points (SD 6.78) CG: mean 4.04 points (SD 9.24)
**Atreja 2018**	NR	NR	IG: SIBDQ QoL at 575 days mean 25.2 points (SD 11.3) CG: not reported
**Carlsen 2017**	Stated as an outcome but no data	NR	Stated as an outcome but no data
**Chauhan 2016**	Study authors did not provide data, but commented there was no significant change.	NR	Study authors did not provide data, but commented there was no significant change.
**Cross 2012**	Seo index scores: IG: mean 122 points (SD 39.3) CG: mean 113.6 points (SD 28) Remission rates at 12 months: IG: n = 19/25 (77%) CG: n = 16/22 (76%)	Relapses at 12 months: IG: 6 CG: 6	IBDQ: IG: mean 178.1 points (unspecified variance measure 32.1) CG: mean 187.3 points (unspecified variance measure 32.2)
**Cross 2019**	HBI: CG: mean 3.7 points (SD 3.6) IG1: mean 4.2 points (SD 3.9) IG2: mean 3.2 points (SD 3.4) SCCAI: CG: mean 1.4 points (SD 1.4) IG1: mean 1.7 points (SD 1.9) IG2: mean 2.0 points (SD 1.8)	CD: CG: 29/79 (36.5%) IG1: 31/79 (39.1%) IG2: 23/78 (29.6%) UC/IC: CG: 7/36 (18.5%) IG1: 8/36 (21.7%) IG2: 13/38 (33.3%)	IBDQ at study end: CG: mean 179.3 points (unspecified variance measure 28.2) IG1: mean 181.5 points (unspecified variance measure 28.2) IG2: mean 179.2 points (unspecified variance measure 32.8)
**De Jong 2017**	NR	Number of flares during the 12 months of follow‐up: "Flares were defined as clinical symptoms indicative of disease activity with, as a rule, concomitant calprotectin of more than 250 μg/g in the stool or active disease determined by endoscopy, MRI, or CT. In daily practice, in case of clinically severe symptoms suggestive for disease activity, the treating physician occasionally judged these symptoms to be evident enough to adjust therapy. Therefore, to capture all clinical flares, clinical episodes were defined as flares if symptoms suggestive of disease activity resulted in a dose escalation or initiation of a new drug to induce remission." IG: mean 0.19 events (unspecified variance measure 0.42) CG: mean 0.19 events (unspecified variance measure 0.44)	SIBDQ at study end: IG mean 54.44 points (unspecified variance measure 9.05) CG: mean 53.71 points (unspecified variance measure 9.87)
**Del Hoyo 2018**	Measured only by proxy (FC levels) and no variance given: "At 24 weeks, the median FC level for clinical activity improved progressively from a baseline value of 490 μg/g to 137 μg/g in IG2(teccu) and from 526 μg/g to 115.5 μg/g in IG1(tele); however, this reduction was smaller in CG, from 330 μg/g to 230 μg/g."	Inactive disease after 24 weeks IG1: 14/21 (66.7%) → 7 relapses IG2: 17/21 (81%) → 4 relapses CG: 15/21 (71.4%) → 6 relapses "Remission was evaluated using the modified HBI for patients with CD. For patients with UC, we used the SCCAI (also known as the Walmsley index) for remote checkups together with the partial Mayo score for face‐to‐face visits. For remote checkups in patients with UC, clinical remission was defined as a Walmsley score ≤ 2,whereas mild‐to‐moderate and severe activities were defined as scores of 3‐5 and >5, respectively. Patients with CD and an HBI < 5 were considered to be in clinical remission, whereas patients with scores of 5‐7, 8‐16, or >16 were considered to have mild, moderate, or severe activity, respectively. In the face‐to‐face visits, clinical remission was defined as a partial Mayo score ≤2 and no individual Mayo sub‐score > 1; scores of 2‐5, 6‐8, and were defined as mild, moderate, and severe disease activity, respectively"	Measured with the IBDQ‐9 and the EQ‐5D. VAS were also used. Median IBDQ‐9 at end: IG1: 53 points IG2: 52.5 points CG: 53 points Median EQ‐5D at end: IG1: 1 point IG2: 1 point CG: 1 point Median VAS values at study end: NR Figure 6 possibly presents variance but unclear if SDs or something else.
**Elkjaer 2010**	NR	SCCAI score > 5 used to define a relapse. Total relapses: IG: 93/169 CG: 87/164 Denmark: IG: 60/105 (51%) + 12 (randomised but did not participate) = 72/117 CG: 60/106 (52%) + 10 (randomised but did not participate) = 70/116 Mean relapses: IG: mean 1.1 events (range 0–6) CG: mean 0.8 events (range 0–4) Ireland: IG: 20/51 (39%) + 1 (randomised but did not participate) = 21/52 CG: 10/41 (24%) + 7 (randomised but did not participate) = 17/48 Mean relapses: IG: mean 0.6 events (range 0–4) CG: 0.2 events (range 0–1)	"Disease specific QoL was improved in the web‐group, as well as general health, vitality, role emotional, and social functioning, compared to control group"
**Heida 2018**	NR	"Disease flares – disease activity requiring therapy intensification (steroid therapy, exclusive enteral nutrition, aminosalicylate dose escalation, or introduction of anti‐TNF antibodies)" Flare‐ups during 52 weeks: IG: 28/84 CG: 29/86	IBD‐specific IMPACT‐III scores Mean change in QoL: IG: 1.32 points CG: −0.32 points No variance provided. IG: 54% reported a positive change. CG: 44% reported a positive change.
**Hughes 2017**	NR	NR	NR
**Ley 2020**	NR	NR	NR
**Malickova 2020**	HBI mean score at end of study (no variance provided): IG: 3.48 points CG: 2.71 points Partial Mayo mean scores at end of study (no variance provided): IG: 2.71 points CG: 2.57 points	NR, study only reported the relapses that required hospitalisation.	NR
**McCombie 2020**	SCCAI: 3 months IG: mean 1.6 points (SD 1.7) CG: mean 0.5 points (SD 0.7) 6 months IG: mean 2.5 points (SD 2.2) CG: mean 1.9 points (SD 2.0) 9 months IG: mean 3.4 points (SD 2.7) CG: mean 2.6 points (SD 4.8) 12 months IG: mean 1.5 points (SD 1.1) CG: mean 1.7 points (SD 1.9) HBI: 3 months IG: mean 4.3 points (SD 3.5) CG: mean 3.6 points (SD 2.3) 6 months: IG: mean 4.2 points (SD 3.8) CG: mean 2.5 points (SD 3.1) 9 months: IG: mean 3.9 points (SD 4.0) CG: mean 1.8 points (SD 1.9) 12 months: IG: mean 2.4 points (SD 3.4) CG: mean 2.0 points (SD 2.5)	UC flare‐ups (months 3–12) IG: 9/13 (70%) CG: 6.14 (42.7%) CD flare‐ups (months 3–12) IG: 17/37 (47.2%) CG: 9/36 (25.7%)	IBDQ (CD) 3 months IG: mean 173.9 points (SD 30.0) CG: mean 160.1 points (SD 35.1) 6 months IG: mean 177.5 points (SD 27.9) CG: mean 163.1 points (SD 36.7) 9 months IG: mean 178.9 points (SD 27.8) CG: mean 159.0 points (SD 31.4) 12 months IG: mean 178.0 points (SD 20.6) CG: mean 167.3 points (SD 32.6) IBDQ (UC) 3 months IG: mean 184.6 points (SD 21.7) CG: mean 186.6 points (SD 21.0) 6 months IG: mean 188.0 points (SD 28.6) CG: mean 175.5 points (SD 31.8) 9 months IG: mean 181.6 points (SD 30.4) CG: mean 181.9 points (SD 27.7) 12 months IG: mean 189.5 points (SD 24.5) CG: mean 179.6 points (SD 24.3)
**Reich 2019**	NR	NR	Median SIBDQ at 6 months (no variance provided): IG: 58 points CG: 57.5 points
**Siegel 2018**	NR	NR	NR
**Stunkel 2012**	NR	NR	IBDQ at study end: IG: mean 172.9 points (unspecified variance measure 26.8) CG: mean 169.3 points (unspecified variance measure 29.3)
**Wang 2020**	NR	NR	NR

CD: Crohn's disease; CG: control group; CT: computerised tomography; EQ‐5D: EuroQol five‐dimension questionnaire; FC: faecal calprotectin; HBI: Harvey‐Bradshaw Index; IBD: inflammatory bowel disease; IBDQ: Inflammatory Bowel Disease Questionnaire; IG: intervention group; IQR: interquartile range; MRI: magnetic resonance imaging; n: number of participants; NR: not reported; QoL: quality of life; SCCAI: Simple Colitis Clinical Activity Index; SD: standard deviation; SIBDQ: Short Inflammatory Bowel Disease Questionnaire TIBS: total inflammation burden scoring; TNF: tumour necrosis factor; UC: ulcerative colitis; VAS: visual analogue scale.

###### Quality of life

Eight studies measured QoL (Cross 2012; Cross 2019; de Jong 2017; Del Hoyo 2018; Elkjaer 2010a; Heida 2018; McCombie 2020; Stunkel 2012).

Four studies on adults provided data that we could use for a meta‐analysis (Cross 2012; Cross 2019; de Jong 2017; McCombie 2020). Web‐based disease monitoring (n = 594) is probably equivalent to usual care (n = 505) for QoL in adults with IBD (SMD 0.08, 95% CI −0.04 to 0.20; Analysis 1.7). We downgraded the certainty of the evidence by one level to moderate for risk of bias concerns (lack of blinding and attrition). Subgroup comparison showed no major differences between mixed IBD, UC, and CD.

A fixed‐effect sensitivity analysis showed no difference in the results (Analysis 1.8).

Stunkel 2012 reported an IBDQ mean of 172.9 (undefined measure of variance 26.8) for the web‐based group and 165.9 (undefined measure of variance 24.7) for the control group. Del Hoyo 2018 reported an IBDQ‐9 mean of 53 and EQ‐5D mean of 1 for the web‐based group, and an IBDQ‐9 mean of 53 and EQ‐5D mean of 1 for the control group, without measures of variance. Elkjaer 2010a provided only commentary on the results of the outcome ("Disease specific QoL was improved in the web‐group, as well as general health, vitality, role emotional, and social functioning, compared to control group"). Heida 2018 provided mean IMPACT changes of 1.32 for the web‐based group and −0.32 for the control group, without a measure of variance. The study authors also commented that 54% of participants in the web‐based group and 44% in the control group reported positive changes. We were unable to reach any conclusions based on these data. We downgraded the certainty of the evidence for all of the above findings to very low for imprecision (very low participant numbers) and risk of bias concerns (all domains).

Table 9 provides more details.

##### Secondary outcomes

###### Number of episodes of accessing healthcare

Eight studies reported number of episodes of accessing healthcare (Carlsen 2017a; Cross 2019; de Jong 2017; Del Hoyo 2018; Elkjaer 2010a; Heida 2018; Malickova 2020; McCombie 2020); however, no meta‐analysis was possible owing to substantial differences between studies in the types of healthcare access reported, methodology, and reporting of the data. We were unable to draw any conclusions on the effects of web‐based disease monitoring compared to usual care on healthcare access. We downgraded the certainty of the evidence to very low for imprecision (very low participant numbers) and risk of bias concerns (all domains). Table 10 provides further details.

**5:** Secondary outcome data

**Study ID**	**2a. Number of episodes of accessing healthcare (outpatient/remote/inpatient)**	**2b. Medication adherence**	**2c. Participant engagement**	**2d. Rate of attendance/engagement (number of planned appointments/interactions attended)**	**2e. Rate of attendance of interactions with professionals**	**2f. Costs or cost/time‐effectiveness (as judged by study authors)**
**Akobeng 2015**	Number of participants with ≥ 1 hospital admissions due to IBD: IG: 1/44 CG: 1/42	NR	NR	Number of consultations scheduled by the hospital for each participant that were not then cancelled by the hospital: IG: median 4.5 (IQR 3–5.3); imputed SD 1.7 CG: median 5 (IQR 3–6); imputed SD 2.2 Number of consultations attended per participant: IG: median 4 (IQR 3–4); imputed SD 0.74 CG: median 3 (IQR 2–4); imputed SD 1.48	Number of participants with ≥ 1 consultation, as allocated before the 12‐ month follow‐up: IG: 36 (82%) CG: 40 (95%)	Costs to the NHS: "Estimates of NHS costs for the intervention (including staff costs and telephone costs) showed that telephone consultation had a mean cost of UK £35.41 per patient consultation compared with £51.12 for face–face consultation, difference £15.71"
**Ankersen 2019**	NR	Adherence to medication was measured by a self‐assessment questionnaire (MARS) MARS score: IG: median 23.57 points (IQR 21.50–24.25); calculated SD 2.03 CG: median 24.17 points (IQR 23.50–24.80); calculated SD 0.96	"The 88 patients that completed the study were asked seven questions at follow‐up. There was no statistical difference between the two intervention groups on any of the seven yes/no questions assessing patient satisfaction."	NR	NR	NR
**Atreja 2018**	NR	NR	NR	NR	NR	NR
**Carlsen 2017**	Outpatient visits: IG: total 85; median 2 (IQR 2–3) CG: total 185; median 8 (IQR 4‐9) On‐demand outpatient visits: IG: total 47; median (IQR 0–3); CG: total 39; median 1 (IQR 0–2) Acute/hospitalisations: IG: total 3; median 0 (IQR 0–0); CG: total 10; median 0 (IQR 0–1) Planned outpatient visits: IG: total 38; median 2 (IQR 1–2); CG: total 146; median 7 (IQR 3–7) Contacts in total: IG: total 88; median 2 (IQR 2–4); CG: total 195; median 8.5 (IQR 4–10)	Mean MARS scores (from trial registration): IG: mean 23.3 points (95% CI 22.9–23.6); calculated SD 0.88 CG: mean 23.3 points (95% CI 22.9–23.7); calculated SD 0.97	"The adherence to the web program was 81% (384/475 expected entries)."	Planned outpatient visits: IG: total 38; median 2 (IQR 1–2) CG: total 146; median 7 (IQR 3–7)	NR	"From a socioeconomic perspective, the reduced school absence and fewer outpatient visits in the web group represent an economic gain, as parents do not require leave from work, and it saves the time and expense of travel to/from our hospital."
**Chauhan 2016**	NR	NR	NR	NR	NR	"The average parking and travel costs for patients randomised to intervention were CAN $25.83, and their average loss of income was CAN $17.00. The median duration of healthcare contact was longer in the intervention group (52 minutes [IQR 38–81] vs 17 minutes [IQR 15.0–21.2]), with wait time was longer in intervention (median 31.6 minutes [IQR 8–56] vs 0 minutes"
**Cross 2012**	NR	Based on the MMAS. For the purpose of evaluating percent of participants adherent to therapy, the variable was dichotomised to "adherent" or "non‐adherent." Any response of yes to one of the 4 items was scored as "non‐adherent." IG: 14/25 (57%) CG: 14/22 (67%)	NR	NR	NR	NR
**Cross 2019**	Extracted from the electronic medical records during 1 year before and after randomization. Post‐randomisation numbers reported as rates adjusted for 100 participants per year (hospitalisations, surgery, emergency department and office visits, procedures, intravenous therapeutics, and telephone and electronic encounters). Unclear if these are only for the randomised participants. CG: 2099 IG1: 2235 IG2: 1935	NR	"Adherence was defined as the completion of 80% (278/348) or more of the weekly or every other week self‐assessments." No data presented.	NR	NR	NR
**De Jong 2017**	Number of hospital admissions, unique participants: IG: 16 CG: 29 Mean outpatient visits: IG: gastroenterologist: mean 1.26 (SD 1.18); nurse: mean 0.29 (0.68); total: mean 1.55 (SD 1.50) CG: gastroenterologist: mean 1.98 (SD 1.19); nurse: mean 0.36 (0.84); total: mean 2.34 (SD 1.64) Mean telephone consultations: IG: gastroenterologist: mean 0.58 (SD 0.98); nurse: mean 0.7 (SD 1.59); total: mean 1.28 (SD 2.06) CG: gastroenterologist: mean 0.84 (SD 1.11): nurse: mean 0.74 (SD 1.9); total: mean 1.57 (SD 2.44) The number of outpatient visits and telephone consultations with gastroenterologists and nurses during the 12‐month period were retrieved from participants’ electronic medical records.	Mean MMAS score: IG: mean 7.01 points (SD 1.40) CG: mean 6.77 points (SD 1.61)	NR	NR	NR	Calculated mean annual direct costs, per participant: IG: EUR 7048 CG EUR 7423 Calculated mean indirect costs, per participant: IG: EUR 1886 CG: EUR 2058
**Del Hoyo 2018**	Outpatient visits: IG1 85 (29.5%) IG2 72 (25%) CG 131 (45.5%) Telephone calls: IG1 118 (66.7%) IG2 12 (6.8%) CG 47 (26.5%) Study authors recorded the number of outpatient visits and telephone consultations for all 3 groups during the study. As these numbers were per participant, we could not use them for meta‐analysis.	Medication adherence according to Morisky‐Green index: IG1 33.3% (7/21) IG2 57.1% (12/21) CG 66.7% (14/21) CG	Participants who adhered to > 80% of checkups (considered compliant): IG1 20 (95.2%) IG2 18 (85.7%) CG 19 (90.5%)	NR	NR	"There is a high probability that the use of the TECCU Web‐platform produces a greater improvement in disease activity at a lower societal cost."
**Elkjaer 2010**	Acute visits: IG: 21 CG: 107 Routine visits: IG: 35 CG: 92 Emails/phone calls: IG: 86/21 CG 7/17	NR	Compliance: IG: 73% CG: 42%	NR	NR	The study authors converted the numbers of medications and professional visits into financial savings for department and found it cost‐effective.
**Heida 2018**	Mean face‐to‐face encounters with health providers: IG: 3.6 CG: 4.3	NR	Compliance with study protocol (> 80% response to alerts): IG: 48 CG: 72 Did not respond to any emails: IG: 10 CG: NR Insufficient compliance (< 80% response to alerts): IG: 26 CG: 14	NR	NR	"Home tele‐monitoring led to a mean annual cost‐saving of €89 per participant in the intention‐to‐treat analysis. The intervention was most cost‐saving in participants who were compliant (mean annual saving 360 euros)."
**Hughes 2017**	NR	NR	Completed at least 1 telephone session: IG: 80% CG: NR	NR	NR	NR
**Ley 2020**	NR	Mean adherence at study end (measured by MPR): IG: 0.539 CG: 0.462	NR	NR	NR	NR
**Malickova 2020**	Median number of visits to doctor per participant IG: 0 CG: 4 Median number of visits to IBD nurse per participant IG: 0.3 CG: 0.9 Median number of hospitalisations IG: 1 CG: 0	NR	IG: 4 non‐compliant CG: NR	NR	NR	"Annual average costs remotely / tele‐medically monitored patient (CZK 2,060 / patient / year) were 25% lower than the cost of the same standardly outpatient patient (CZK 2,580 / patient / year)"
**McCombie 2020**	Gastroenterologist appointments: IG: mean 0.6 (SD 0.9) CG: mean 1.7 (SD 0.8) Surgical appointments: IG: mean 0.1: (SD 0.4) CG: mean 0.1: (SD 0.4) IBD hospitalisations: IG: mean 0.1 (SD 0.3) CG: mean 0.1 (SD 0.4) Nights in hospital: IG: mean 0.1 (SD 0.4) CG: mean 0.8 (SD 3.9)	NR	"At the end of 12 months, patients in the smartphone app group completed 2 system usability scales. The questionnaires asked about the instructions provided for the apps, what issues with the apps they experienced during the study, and whether they would keep using the apps in the future and recommend them to other people with IBD." No data presented.	For IBDoc, 15 (30%) completed all readings. 14 (28%) completed 4. 6 (12%) completed 3. 2 (4%) completed 2. 6 (12%) completed 1. 7 (14%) completed 0. For IBDsmart, 25 (50%) completed all readings. 9 (18%) completed 4. 7 (14%) completed 3. 1 (2%) completed 2. 7 (14%) completed 1. 1 (2%) completed 0.	NR	NR
**Reich 2019**	NR	NR	33% reported logging onto MyChart monthly, whereas 32% logged on weekly, and 13% logged on every other week.	NR	NR	NR
**Siegel 2018**	NR	NR	NR	NR	NR	NR
**Stunkel 2012**	NR	NR	"The experimental group did feel that the mobile app was easy to use and subjectively improved their ability to track and correlate symptoms"	NR	NR	NR
**Wang 2020**	NR	Month 1 MMAS < 6: IG: 27 CG: 34 MMAS ≥ 6: IG: 93 CG: 85 Month 2 MMAS < 6: IG: 30 CG: 35 MMAS ≥ 6: IG: 90 CG: 84 Month 4 MMAS < 6: IG: 23 CG: 37 MMAS ≥ 6: IG: 97 CG: 82 Month 6 MMAS < 6: IG: 22 CG: 42 MMAS ≥ 6: IG: 98 CG: 77	NR	NR	NR	NR

IBD: inflammatory bowel disease; IQR: interquartile range; MARS: Medication Adherence Rating Scale; MMAS: Morisky Medication Adherence Scale; MPR: Medication Possession Ratio; NHS: UK National Health Service; SD: standard deviation.

###### Medication adherence

Five studies reported medication adherence (Carlsen 2017a; Cross 2012; de Jong 2017; Del Hoyo 2018; Wang 2020). Four studies provided data suitable for meta‐analysis: continuous data in de Jong 2017 and Carlsen 2017a, and dichotomous data in Cross 2012 and Del Hoyo 2018.

The analysis of continuous data from de Jong 2017 showed that web‐based disease monitoring (n = 340) compared to usual care (n = 331) probably leads to slightly higher medication adherence in adults (MD 0.24 points, 95% CI 0.01 to 0.47; Analysis 1.9). We downgraded the certainty of the evidence by one level to moderate for risk of bias due to lack of blinding.

The analysis of continuous data from Carlsen 2017a showed no difference between web‐based disease monitoring (n = 15) and usual care (n = 18) in terms of their effect on medication adherence in children, although the results are very uncertain (MD 0.00, 95% CI −0.63 to 0.63; Analysis 1.10). We downgraded the certainty of the evidence to very low for imprecision (very low participant numbers) and risk of bias (lack of blinding).

Meta‐analysis of the dichotomous data showed no difference between web‐based disease monitoring (n = 26/46) and usual care (n = 28/43) in terms of their effect on medication adherence in adults, although the results are very uncertain (RR 0.87, 95% CI 0.62 to 1.21; 2 studies; Analysis 1.11). We downgraded the certainty of the evidence to very low for imprecision (very low numbers of events) and risk of bias concerns (lack of blinding and attrition). Subgroup comparison showed no major differences between mixed IBD and UC.

Wang 2020 reported MMAS scores of less than six points for 22 participants in the web‐based group and 42 in the control group, and scores of more than or equal to six points for 98 participants in the web‐based group and 77 in the control group at six months. We were unable to draw any conclusions from these data. We downgraded the certainty of the evidence to very low for imprecision (low event numbers) and risk of bias concerns (blinding and selective reporting).

Table 10 provides further details.

###### Participant engagement

Eleven studies reported or commented on participant engagement (Ankersen 2019; Carlsen 2017a; Cross 2019; Del Hoyo 2018; Elkjaer 2010a; Heida 2018; Hughes 2017; Malickova 2020; McCombie 2020; Reich 2019; Stunkel 2012); however, no meta‐analysis was possible owing to substantial differences between studies in the types of participant engagement reported, methodology, and reporting of the data. We were unable to draw any conclusions on the effects of web‐based disease monitoring compared to usual care on participant engagement. We downgraded the certainty of the evidence to very low for imprecision (very low participant numbers) and risk of bias concerns (all domains). Table 10 provides further details.

###### Rate of attendance or engagement with any or all elements of the intervention

Three studies reported attendance or engagement with the intervention (Akobeng 2015; Carlsen 2017a; McCombie 2020); however, meta‐analysis was not possible owing to differences in how studies reported this outcome. We were unable to draw any conclusions on the effects of web‐based disease monitoring compared to usual care on attendance or engagement rate. We downgraded the certainty of the evidence to very low for imprecision (very low participant numbers) and risk of bias concerns (lack of blinding). Table 10 provides further details.

###### Rate of attendance of interactions with healthcare professionals

Akobeng 2015 and Del Hoyo 2018 reported attendance of interactions with healthcare professionals; however, meta‐analysis was not possible owing to differences in how the two studies reported this outcome. We were unable to draw any conclusions on the effects of web‐based disease monitoring compared to usual care on rate of attendance of interactions with healthcare professionals. We downgraded the certainty of the evidence to very low for imprecision (very low participant numbers) and risk of bias concerns (lack of blinding). Table 10 provides further details.

###### Costs or cost/time‐effectiveness

Eight studies provided estimations of costs or cost/time‐effectiveness (Akobeng 2015; Carlsen 2017a; Chauhan 2016; de Jong 2017; Del Hoyo 2018; Elkjaer 2010a; Heida 2018; Malickova 2020); however, meta‐analysis was not possible owing to differences in how studies reported this outcome. We were unable to draw any conclusions on the effects of web‐based disease monitoring compared to usual care on costs or cost/time‐effectiveness. We downgraded the certainty of the evidence to very low for imprecision (very low participant numbers) and risk of bias concerns (all domains). Table 10 provides further details.

Owing to lack of data, we were unable to perform subgroup and sensitivity analyses prespecified in our protocol.

#### 2. Web‐based disease monitoring versus sham monitoring

Three studies evaluated web‐based disease monitoring versus sham monitoring (Atreja 2018; Ley 2020; Reich 2019). We were unable to perform meta‐analyses for any primary or secondary outcomes (Table 2). 

##### Primary outcomes

###### Disease activity

No studies reported disease activity.

###### Flare‐ups or relapse

No studies reported flare‐ups or relapse.

###### Quality of life

Atreja 2018 provided QoL results only for the web‐based group and not the sham group, while Reich 2019 provided QoL means at six months but without variance measures. We were unable to draw any conclusions on the effects of web‐based disease monitoring compared to sham monitoring on QoL. We downgraded the certainty of the evidence to very low for imprecision (very low participant numbers) and risk of bias concerns (all domains). Table 9 provides further details.

##### Secondary outcomes

###### Number of episodes of accessing healthcare

No studies reported healthcare access.

###### Medication adherence

Ley 2020 provided medication adherence means at study end but without any variance measures. We were unable to draw any conclusions on the effects of web‐based disease monitoring compared to sham monitoring on medication adherence. We downgraded the certainty of the evidence to very low for imprecision (very low participant numbers) and risk of bias concerns (selection bias, blinding, attrition bias, and reporting bias). Table 10 provides further details.

###### Participant engagement

Reich 2019 reported rates of participants logging onto their web application (monthly, weekly, and every other week). We were unable to draw any conclusions on the effects of web‐based disease monitoring compared to sham monitoring on participant engagement. We downgraded the certainty of the evidence to very low for imprecision (very low participant numbers) and risk of bias concerns (selection bias, blinding, and attrition bias). Table 10 provides further details.

###### Rate of attendance or engagement with any or all elements of the intervention

No studies reported attendance or engagement rate.

###### Rate of attendance of interactions with healthcare professionals

No studies reported interactions with professionals.

###### Costs or cost/time‐effectiveness

No studies reported costs or cost/time‐effectiveness.

#### 3. Web‐based disease monitoring versus self‐screening

One study evaluated web‐based disease monitoring versus self‐screening (Ankersen 2019). We were unable to perform meta‐analyses for any primary or secondary outcomes (Table 3). 

##### Primary outcomes

###### Disease activity

The authors of Ankersen 2019 devised their own classification system for disease activity, presenting SCCAI, HBI, and TIBS mean scores without variance on their "traffic light" classification over one year. We were unable to draw any conclusions on the effects of web‐based disease monitoring compared to self‐screening on disease activity. We downgraded the certainty of the evidence to very low for imprecision (very low participant numbers) and risk of bias concerns (selection bias, blinding, and reporting bias). Table 9 provides further details.

###### Flare‐ups or relapse

Ankersen 2019 reported combined moderate and severe relapse numbers based on SCCAI and FC levels; however, the denominator in this calculation (total number of patients) far exceeded the number of people randomised, so it was unclear if these relapses were based on randomised data. We were unable to draw any conclusions on the effects of web‐based disease monitoring compared to self‐screening on relapses or flare‐ups. We downgraded the certainty of the evidence to very low for imprecision (very low participant numbers) and risk of bias concerns (selection bias, blinding, and reporting bias). Table 9 provides further details.

###### Quality of life

Ankersen 2019 reported mean changes in QoL in the two groups, but it was unclear if these groups comprised the randomised participants. We were unable to draw any conclusions on the effects of web‐based disease monitoring compared to self‐screening on QoL. We downgraded the certainty of the evidence to very low for imprecision (very low participant numbers) and risk of bias concerns (selection bias, blinding, and reporting bias). Table 9 provides further details.

##### Secondary outcomes

###### Number of episodes of accessing healthcare

 Ankersen 2019 did not report healthcare access.

###### Medication adherence

Ankersen 2019 reported median (interquartile range (IQR)) adherence values for the two groups, but it was unclear if these groups comprised the randomised participants. We were unable to draw any conclusions on the effects of web‐based disease monitoring compared to self‐screening on medication adherence. We downgraded the certainty of the evidence to very low for imprecision (very low participant numbers) and risk of bias concerns (selection bias, blinding, and reporting bias). Table 10 provides further details.

###### Participant engagement

Ankersen 2019 reported no "statistical difference between the two intervention groups on any of the seven yes/no questions assessing patient satisfaction". We were unable to draw any conclusions on the effects of web‐based disease monitoring compared to self‐screening on participant engagement. We downgraded the certainty of the evidence to very low for imprecision (very low participant numbers) and risk of bias concerns (selection bias, blinding, and reporting bias). Table 10 provides further details.

###### Rate of attendance or engagement with any or all elements of the intervention

 Ankersen 2019 did not report attendance or engagement rate.

###### Rate of attendance of interactions with healthcare professionals

 Ankersen 2019 did not report interactions with professionals.

###### Costs or cost/time‐effectiveness

 Ankersen 2019 did not report costs or cost/time‐effectiveness.

#### 4. Telephone‐based disease monitoring versus face‐to‐face monitoring

Three studies evaluated telephone‐based disease monitoring versus face‐to‐face monitoring: two enrolled adults (Chauhan 2016; Del Hoyo 2018), and one enrolled children (Akobeng 2015).

##### Primary outcomes

Table 4 presents the effect measures (where calculated) and GRADE judgements for the primary outcomes.

###### Disease activity

Two studies reported disease activity, but neither provided data suitable for meta‐analysis (Chauhan 2016; Del Hoyo 2018; Table 9). 

Chauhan 2016 reported no significant change. Del Hoyo 2018 measured disease activity only by proxy (FC levels) and provided no variance measure. We were unable to draw any conclusions on the effects of telephone‐based disease monitoring compared to face‐to‐face monitoring on disease activity. We downgraded the certainty of the evidence to very low for imprecision (very low participant numbers) and risk of bias concerns (selective reporting). Table 9 provides further details.

###### Flare‐ups or relapse

All three studies reported flare‐ups or relapse (Akobeng 2015; Chauhan 2016; Del Hoyo 2018).

Del Hoyo 2018 provided data suitable for meta‐analysis from an adult population. We found no difference between telephone‐based disease monitoring (n = 7/21) and face‐to‐face monitoring (n = 6/21) in terms of their effect on the occurrence of flare‐ups or relapses in adults with IBD, but the results are very uncertain (RR 1.17, 95% CI 0.47 to 2.89; Analysis 2.1). We downgraded the certainty of the evidence to very low for imprecision (very low participant numbers) and risk of bias concerns (lack of blinding). 

Akobeng 2015 provided data suitable for meta‐analysis from a paediatric population. We found no difference between telephone‐based disease monitoring (n = 1/44) and face‐to‐face monitoring (n = 4/42) in terms of their effect on the occurrence of flare‐ups or relapses in children with IBD, but the results are very uncertain (RR 0.24, 95% CI 0.03 to 2.05; Analysis 2.2). We downgraded the certainty of the evidence to very low for imprecision (very low participant numbers) and risk of bias concerns (lack of blinding).

Chauhan 2016 reported "no significant change" but provided no data. We were unable to draw any conclusions from this information. We downgraded the certainty of the evidence to very low for imprecision (very low participant numbers) and risk of bias concerns (lack of blinding and selective reporting).

Table 9 provides further details.

###### Quality of life

All three studies reported QoL (Akobeng 2015; Chauhan 2016; Del Hoyo 2018).

Akobeng 2015 provided data suitable for meta‐analysis from a paediatric population. It is unclear whether telephone‐based disease monitoring (n = 44) compared to face‐to‐face monitoring (n = 42) affects QoL in children with IBD (MD 7.00 points, 95% CI −0.29 to 14.29; Analysis 2.3). We downgraded the certainty of the evidence to very low for imprecision (very low participant numbers) and risk of bias concerns (lack of blinding).

Del Hoyo 2018 reported QoL means without measures of variance. We were unable to draw any conclusions based on these data. We downgraded the certainty of the evidence to very low for imprecision (very low participant numbers) and risk of bias concerns (lack of blinding and selective reporting).

Chauhan 2016 reported "no significant change" but provided no data. We were unable to draw any conclusions from this information. We downgraded the certainty of the evidence to very low for imprecision (very low participant numbers) and risk of bias concerns (lack of blinding and selective reporting).

Table 9 provides further details.

##### Secondary outcomes 

###### Number of episodes of accessing healthcare

Akobeng 2015 and Del Hoyo 2018 reported number of episodes of accessing healthcare.

Akobeng 2015 reported numbers of participants in each consultation group that had one or more hospital admissions due to IBD. It is unclear whether telephone‐based disease monitoring (n = 1/44) compared to face‐to‐face monitoring (n = 1/42) affects the number of episodes of accessing healthcare in children with IBD (RR 0.95, 95% CI 0.06 to 14.77; Analysis 2.4). We downgraded the certainty of the evidence to very low for imprecision (very low participant numbers) and risk of bias concerns (lack of blinding).

Del Hoyo 2018 reported the number of outpatient visits and telephone consultations. We were unable to draw any conclusions from these data. We downgraded the certainty of the evidence to very low for imprecision (very low participant numbers) and risk of bias concerns (lack of blinding). Table 10 provides further details.

###### Medication adherence

Only Del Hoyo 2018 reported numbers of participants adhering to their medication. It is unclear whether telephone‐based disease monitoring (n = 7/21) compared to face‐to‐face monitoring (n = 14/21) affects medication adherence in adults with IBD (RR 0.50, 95% CI 0.25 to 0.98; Analysis 2.5). We downgraded the certainty of the evidence to very low for imprecision (very low participant numbers) and risk of bias concerns (lack of blinding). Table 10 provides further details.

###### Participant engagement

Only Del Hoyo 2018 reported participant engagement, specifically the number of participants who adhered to more than 80% of checkups planned in the study protocol. It is unclear whether telephone‐based disease monitoring (n = 20/21) compared to face‐to‐face monitoring (n = 19/21) affects participant engagement in adults with IBD (RR 1.05, 95% CI 0.89 to 1.25; Analysis 2.6). We downgraded the certainty of the evidence to very low for imprecision (very low participant numbers) and risk of bias concerns (lack of blinding). Table 10 provides further details.

###### Rate of attendance or engagement with any or all elements of the intervention

Only Akobeng 2015 reported attendance or engagement rate, specifically the number of scheduled consultations that each participant missed. It is unclear whether telephone‐based disease monitoring (n = 36) compared to face‐to‐face monitoring (n = 40) affects attendance or engagement rate in children with IBD (MD 1.00, 95% CI 0.48 to 1.52; Analysis 2.8). We downgraded the certainty of the evidence to very low for imprecision (very low participant numbers) and risk of bias concerns (lack of blinding). Table 10 provides further details.

###### Rate of attendance of interactions with healthcare professionals

Only Akobeng 2015 reported attendance of interactions with healthcare professions, specifically the number of participants who attended at least one scheduled consultation before the 12‐month follow‐up. It is unclear whether telephone‐based disease monitoring (n = 36/44) compared to face‐to‐face monitoring (n = 40/42) affects the rate of attendance of interactions with healthcare professionals in children with IBD (RR 0.86, 95% CI 0.74 to 1.00; Analysis 2.9). We downgraded the certainty of the evidence to very low for imprecision (very low participant numbers) and risk of bias concerns (lack of blinding). Table 10 provides further details.

###### Costs or cost/time‐effectiveness

All three studies provided narrative estimates on costs or time‐effectiveness (Akobeng 2015; Chauhan 2016; Del Hoyo 2018). We were unable to draw any conclusions on the effects of telephone‐based disease monitoring compared to face‐to‐face monitoring on cost or cost/time‐effectiveness. We downgraded the certainty of the evidence to very low for imprecision (very low participant numbers) and risk of bias concerns (lack of blinding). Table 10 provides further details.

#### 5. Cognitive behavioural therapy manual and telephone support versus usual care

One study evaluated CBT manual and telephone support versus usual care (Hughes 2017).

We were unable to perform meta‐analyses for any primary or secondary outcomes (Table 5). 

##### Primary outcomes

###### Disease activity

Hughes 2017 did not report disease activity.

###### Flare‐ups of relapse

Hughes 2017 did not report flare‐ups or relapse.

###### Quality of life

Hughes 2017 did not report QoL.

##### Secondary outcomes

###### Number of episodes of accessing healthcare

Hughes 2017 did not report healthcare access.

###### Medication adherence

Hughes 2017 did not report medication adherence.

###### Participant engagement

Hughes 2017 reported rates of participants completing at least one telephone session only for the intervention group. We were unable to draw any conclusions on the effects of CBT manuals and telephone support compared to usual care on participant engagement. We downgraded the certainty of the evidence to very low for imprecision (very low participant numbers) and risk of bias concerns (randomisation, blinding, attrition, and selective reporting).

###### Rate of attendance or engagement with any or all elements of the intervention

Hughes 2017 did not report attendance or engagement rate.

###### Rate of attendance of interactions with healthcare professionals

Hughes 2017 did not report interactions with professionals.

###### Costs or cost/time‐effectiveness

Hughes 2017 did not report costs or cost/time‐effectiveness.

## Discussion

### Summary of main results

This review included a wide range of interventions in a very contemporaneous area of interest. Since 2020, almost all people with IBD have had some elements of their care delivered by telehealth, but this approach had already formed a part of IBD healthcare provision for some time.

The studies included in this review demonstrate the different means employed to deliver remote healthcare to people with IBD. Web‐based disease monitoring was the most commonly studied intervention and was compared to standard or usual care in 12 studies, with just three adding a sham or control web application to the control group. A single study compared web‐based disease monitoring with self‐screening, three studies compared telephone‐based disease monitoring with face‐to‐face monitoring, and one study evaluated a CBT manual combined with telephone support versus usual care.

Most studies compared a form of remote telehealth to normal or usual care, but descriptions of normal care were limited, and no studies specified whether standard care groups were offered remote care, formally or informally.

The analysis for the most common comparison (web‐based monitoring versus usual care) produced the following results.

- There is probably no difference between the interventions in IBD disease activity in adults.
- There is probably no difference between the interventions in IBD flare‐ups or relapse in adults.
- There may be no difference between the interventions in IBD flare‐ups or relapse in children.
- There is probably no difference between the interventions in QoL in adults.
- Web‐based monitoring compared to usual care probably improves medication adherence slightly in adults.

The poor reporting of other outcomes measures severely limited the scope for meta‐analysis, and the certainty of evidence was very low.

### Overall completeness and applicability of evidence

Further clarification on the specifics of the web‐based monitoring would support better replication and dissemination (Table 7; Table 8). Unlike pharmacological intervention reviews, reviews of this type should establish not only whether an intervention is effective or safe, but also what specific components of the intervention are effective. Most studies included in this review do not provide this information. Lack of detail is a recognised problem in non‐pharmacological trial reporting. An analysis of non‐pharmacological intervention trials found that 61% of reports did not provide details of the primary intervention, although trial authors forwarded this information on request in 72% of cases (Hoffman 2013). In this review, we received only minimal information from study authors when we contacted them. It is important that future studies rectify this gap in the evidence base.

The choice of outcomes in the included studies was another concern. The primary outcomes appeared somewhat arbitrary and involved many clinical measures. For pharmacological studies, national governing bodies often mandate the primary outcomes, but as this is not the case for studies of non‐pharmacological interventions, the analysis in this review is limited. In addition, follow‐up duration was generally short.

Most studies used web‐based disease monitoring as the focus for remote care. Few studies evaluated other remote approaches. It appears that many ongoing studies are focusing on other forms of remote care (possibly as a result of the COVID‐19 pandemic), and future updates of this review will likely include these interventions.

We excluded studies where remote monitoring of blood or faecal tests was the only form of monitoring, as this was a proxy for direct patient outcomes. This could be considered an incomplete aspect of our review and a potential focus of a new review.

Finally, standard care was a frequent comparator in the included studies, but no studies provided clear descriptions of standard care in terms of the content, form, frequency, and professionals involved. Without this information, it is unclear to what extent each intervention differed from its respective control. As a result, the completeness and utility of the evidence is limited.

### Quality of the evidence

There were significant issues related to risk of bias in the studies included in this review. Despite our requests to authors of included studies, we received few data to change our judgements in these key areas.

Most studies did not blind participants, personnel, or outcome assessors, but this can be considered acceptable given the context of the review. As we explained in a previous review (Gordon 2022), research has demonstrated that even in double‐blind trials, participant expectancies can limit the validity of the design; assessing participants' beliefs about their treatment could help to overcome this issue (Colagiuri 2010). Nevertheless, blinding remains a concern and a potential limitation of the included studies in this review, and we have downgraded the certainty of the evidence for all our outcomes accordingly.

Reporting of the interventions themselves is another source of potential bias, as it is difficult to determine what specific interventions each study delivered. As discussed in Overall completeness and applicability of evidence, unclear reporting is a recognised problem within non‐pharmacological intervention studies (Hoffman 2013), and within health education systematic reviews (Gordon 2016), although the GRADE approach does not explicitly identify this issue (Gordon 2020). Lack of detail in the reporting of interventions constitutes the most serious problem with the evidence base, limiting the utility of our outcomes, because these interventions cannot be replicated or disseminated.

The outcome of paediatric flare‐ups or relapses for web‐based disease monitoring compared to usual care was downgraded twice for imprecision (low participant numbers) and risk of bias concerns (blinding and attrition).

All reported primary outcomes for telephone‐based disease monitoring compared to face‐to‐face monitoring were downgraded three times for serous imprecision (very low participant numbers) and risk of bias concerns.

The only secondary outcome we were able to meta‐analyse was medication adherence for web‐based disease monitoring compared to usual care. We considered the evidence for this outcome based on continuous data in adults to be of moderate certainty, downgrading once for risk of bias; and we considered the evidence based on continuous data in children and the evidence based on dichotomous data in adults to be of very low certainty, downgrading for very serious imprecision and risk of bias concerns.

### Potential biases in the review process

Clinical heterogeneity is a major concern in this review. Most studies included people with both CD and UC at different disease states. Had we excluded studies that did not differentiate between CD and UC (most studies), we would have lost a key source of evidence in this area. Nevertheless, this clearly introduces a source of bias.

Although some studies analysed IBD populations as one cohort while others analysed UC and CD populations separately, and despite the mix of disease states in the included studies, we do not consider indirectness to be an issue. The constituents of the interventions were homogenous in their scope for web‐based monitoring, and varied only in the type of telehealth method adopted. There is no clinical evidence to suggest indirectness between subgroups of IBD and disease state. However, we recognise the variation in the methods used by the included studies may be a limitation of this review. Our outcomes are direct measures for efficacy and safety in IBD treatment.

We decided to only include studies where the remote component was the primary focus and not part of a larger package, and we may have missed studies with relevant evidence as a result.

### Agreements and disagreements with other studies or reviews

This is the first Cochrane Review on remote care for people with IBS.

One systematic review from 2014 concluded that distance management of IBD significantly decreased clinic visit utilisation but did not significantly affect relapse rates or hospital admission rates (Huang 2014). Another systematic review, published in 2022, concluded that digital health technologies may be effective in decreasing healthcare utilisation and costs, though may not improve risk of relapse, QoL, or treatment adherence in people with IBD (Nguyen 2022). Similarly, we found no effect on relapse rates and QoL in comparison to usual care, but we had insufficient evidence to judge clinic visits, hospital admissions, and costs. The evidence we found on medication adherence was heterogeneous, with one meta‐analysis suggesting telehealth may be non‐inferior to usual care (though the evidence is very uncertain), and another suggesting telehealth is probably slightly better than usual care.

The international guidelines for IBD provide no evidence base to support the use of remote telehealth as a standalone or replacement intervention, only as an addendum to normal care (Feuerstein 2020; Feuerstein 2021; Forducey 2012; Ko 2019; Lamb 2019).

## Authors' conclusions

**Implications for practice**

The evidence in this review demonstrates that web‐based disease monitoring is probably no different to standard care when considering disease activity, occurrence of flare‐ups or relapse, and quality of life in adults with inflammatory bowel disease (IBD), and it probably improves medication adherence slightly. Evidence in children is limited.

The effects of web‐based disease monitoring versus usual care on the remaining secondary outcomes are unclear, as are the effects of the other telehealth interventions included in our review, as there are insufficient high‐quality data.

**Implications for research**

For the comparison web‐based monitoring versus standard care, we consider that further studies are unlikely to change the findings of this review. Several outcomes demonstrate that the intervention is no more effective than standard care.

Longer‐term studies with outcome measures after some years could provide more relevant findings for a chronic disease such as IBD. Additionally, future studies should provide more detailed reports of the interventions to allow practical dissemination and replication. This includes details on the type and number of staff needed, resources, equipment, costs, accessibility, and data security. Further studies on children could be useful, as well as studies that examine differences in efficacy between subgroups (e.g. sex or socio‐economic status).

There is also a need to investigate the impact of other forms of remote telehealth, including those reported in this review in small numbers. Nine ongoing studies are currently examining other remote care strategies.

## History

Protocol first published: Issue 4, 2021

## Appendices

### Appendix 1. CENTRAL Search strategy (via Ovid Evidence‐Based Medicine Reviews Database)

1. exp Inflammatory bowel diseases/
2. (inflammatory bowel disease* or IBD or UC or CD).tw,kw.
3. crohn*.tw,kw.
4. (colitis or regional enteritis or proctocolitis or colorectitis).tw,kw.
5. or/1‐4
6. (phone* or phoning or telephone* or telecom or telecommunicat* or tele‐communicat* or teleconferenc* or tele‐conferenc* or telegraph* or tele‐graph*).tw,kw.
7. exp Telecommunications/
8. (Electronic Mail* or email* or e‐mail* or Telefacsimile or fax or telehealth or tele‐health or telemed* or tele‐med* or ehealth or e‐health or mhealth or m‐health).tw,kw.
9. (instant messag* or SMS or text or texting).tw,kw.
10. (webcast* or webina* or virtual conferenc*).tw,kw.
11. ((web or internet or online or video or virtual or mobile or digital*) adj5 (care or communicat* or health* or medicine* or medical or clinic* or physician* or treat* or therap* or intervention* or conferenc* or connect*)).tw,kw.
12. (mobile or hotline or videoconferenc* or wireless).tw,kw.
13. mobile applications/ or web browser/
14. (remote* adj5 (care or communicat* or health* or medicine* or medical or clinic* or physician* or treat* or therap* or intervention* or conferenc* or connect*)).tw,kw.
15. (GoToMeeting or GoToWebinar or zoom meeting or spotMe or TurboMeeting or Livestorm).tw,kw.
16. (Google Meet* or Cisco Webex or Microsoft Teams or join*me).tw,kw.
17. or/6‐16
18. 5 and 17

### Appendix 2. MEDLINE Search strategy (via Ovid)

1. exp Inflammatory bowel diseases/
2. (inflammatory bowel disease* or IBD or UC or CD).tw,kw.
3. crohn*.tw,kw.
4. (colitis or regional enteritis or proctocolitis or colorectitis).tw,kw.
5. or/1‐4
6. (phone* or phoning or telephone* or telecom or telecommunicat* or tele‐communicat* or teleconferenc* or tele‐conferenc* or telegraph* or tele‐graph*).tw,kw.
7. exp Telecommunications/
8. (Electronic Mail* or email* or e‐mail* or Telefacsimile or fax or telehealth or tele‐health or telemed* or tele‐med* or ehealth or e‐health or mhealth or m‐health).tw,kw.
9. (instant messag* or SMS or text or texting).tw,kw.
10. (webcast* or webina* or virtual conferenc*).tw,kw.
11. ((web or internet or online or video or virtual or mobile or digital*) adj5 (care or communicat* or health* or medicine* or medical or clinic* or physician* or treat* or therap* or intervention* or conferenc* or connect*)).tw,kw.
12. (mobile or hotline or videoconferenc* or wireless).tw,kw.
13. mobile applications/ or web browser/
14. (remote* adj5 (care or communicat* or health* or medicine* or medical or clinic* or physician* or treat* or therap* or intervention* or conferenc* or connect*)).tw,kw.
15. (GoToMeeting or GoToWebinar or zoom meeting or spotMe or TurboMeeting or Livestorm).tw,kw.
16. (Google Meet* or Cisco Webex or Microsoft Teams or join*me).tw,kw.
17. or/6‐16
18. 5 and 17
19. randomized controlled trial.pt.
20. controlled clinical trial.pt.
21. random*.ab.
22. placebo.ab.
23. trial.ab.
24. groups.ab.
25. or/19‐24
26. exp animals/ not humans.sh.
27. 25 not 26
28. 18 and 27

Note: Lines 19‐27. RCT filter: Cochrane Highly Sensitive Search Strategy for identifying randomized trials in MEDLINE: sensitivity‐maximizing version (2008 revision); Ovid format. (Lefebvre 2022). We made the following minor revisions: we used “random*” instead of “randomized.ab” or “randomly.ab.” to capture word variations such as “randomised, randomization, random”; we removed “drug therapy.fs.” from the above filter as this review is not related to drug therapy.“

### Appendix 3. Embase Search strategy (via Ovid)

1. exp inflammatory bowel disease/
2. (inflammatory bowel disease* or IBD or UC or CD).tw,kw.
3. crohn*.tw,kw.
4. (colitis or regional enteritis or proctocolitis or colorectitis).tw,kw.
5. or/1‐4
6. (phone* or phoning or telephone* or telecom or telecommunicat* or tele‐communicat* or teleconferenc* or tele‐conferenc* or telegraph* or tele‐graph*).tw,kw.
7. telecommunication/ or telephone/ or text messaging/ or fax/
8. (Electronic Mail* or email* or e‐mail* or Telefacsimile or fax or telehealth or tele‐health or telemed* or tele‐med* or ehealth or e‐health or mhealth or m‐health).tw,kw.
9. (instant messag* or SMS or text or texting).tw,kw.
10. (webcast* or webina* or virtual conferenc*).tw,kw.
11. ((web or internet or online or video or virtual or mobile or digital*) adj5 (care or communicat* or health* or medicine* or medical or clinic* or physician* or treat* or therap* or intervention* or conferenc* or connect*)).tw,kw.
12. (mobile or hotline or videoconferenc* or wireless).tw,kw.
13. e‐mail/ or hotline/ or mobile phone/ or videoconferencing/ or webcast/ or wireless communication/ or exp web browser/
14. (remote* adj5 (care or communicat* or health* or medicine* or medical or clinic* or physician* or treat* or therap* or intervention* or conferenc* or connect*)).tw,kw.
15. (GoToMeeting or GoToWebinar or zoom meeting or spotMe or TurboMeeting or Livestorm).tw,kw.
16. (Google Meet* or Cisco Webex or Microsoft Teams or join*me).tw,kw.
17. or/6‐16
18. 5 and 17
19. random:.tw.
20. placebo:.mp.
21. double‐blind:.tw.
22. or/19‐21
23. exp animal/ not human/
24. 22 not 23
25. 18 and 24

Note: Line 19‐22. Hedges Best balance of sensitivity and specificity filter for identifying "therapy studies" in Embase.

### Appendix 4. PsycInfo Search strategy (via Ovid)

1. ulcerative colitis/
2. (inflammatory bowel disease* or IBD or UC or CD).tw.
3. crohn*.tw.
4. (colitis or regional enteritis or proctocolitis or colorectitis).tw.
5. or/1‐4
6. (phone* or phoning or telephone* or telecom or telecommunicat* or tele‐communicat* or teleconferenc* or tele‐conferenc* or telegraph* or tele‐graph*).tw.
7. exp communications media/
8. (Electronic Mail* or email* or e‐mail* or Telefacsimile or fax or telehealth or tele‐health or telemed* or tele‐med* or ehealth or e‐health or mhealth or m‐health).tw.
9. (instant messag* or SMS or text or texting).tw.
10. (webcast* or webina* or virtual conferenc*).tw.
11. ((web or internet or online or video or virtual or mobile or digital*) adj5 (care or communicat* or health* or medicine* or medical or clinic* or physician* or treat* or therap* or intervention* or conferenc* or connect*)).tw.
12. (mobile or hotline or videoconferenc* or wireless).tw.
13. exp mobile applications/
14. (remote* adj5 (care or communicat* or health* or medicine* or medical or clinic* or physician* or treat* or therap* or intervention* or conferenc* or connect*)).tw.
15. (GoToMeeting or GoToWebinar or zoom meeting or spotMe or TurboMeeting or Livestorm).tw.
16. (Google Meet* or Cisco Webex or Microsoft Teams or join*me).tw.
17. or/6‐16
18. 5 and 17
19. random:.tw.
20. 18 and 19

Note: line 19. PsycINFO RCT filter: Eady 2008; [Ovid]‐ Single term Best sensitivity &Best specificity.

### Appendix 5. CINAHL (via EBSCO)

S18 S17 (Limiters ‐ Exclude MEDLINE records)

S17 S15 AND S16

S16 MH "treatment outcomes+" OR MH "experimental studies+" or random*

S15 S3 AND S14

S14 S4 OR S5 OR S6 OR S7 OR S8 OR S9 OR S10 OR S11 OR S12 OR S13

S13 TX Google Meet* or Cisco Webex or Microsoft Teams or join*me

S12 TX GoToMeeting or GoToWebinar or zoom meeting or spotMe or TurboMeeting or Livestorm

S11 TX remote* AND TX ( care or communicat* or health* or medicine* or medical or clinic* or physician* or treat* or therap* or intervention* or conferenc* or connect* )

S10 TX mobile or hotline or videoconferenc* or wireless

S9 TX ( web or internet or online or video or virtual or mobile or digital* ) AND TX ( care or communicat* or health* or medicine* or medical or clinic* or physician* or treat* or therap* or intervention* or conferenc* or connect* )

S8 TX webcast* or webina* or virtual conferenc*

S7 TX instant messag* or SMS or text or texting

S6 TX Electronic Mail* or email* or e‐mail* or Telefacsimile or fax or telehealth or tele‐health or telemed* or tele‐med* or ehealth or e‐health or mhealth or m‐health

S5 (MH "Telecommunications")

S4 TX phone* or phoning or telephone* or telecom or telecommunicat* or tele‐communicat* or teleconferenc* or tele‐conferenc* or telegraph* or tele‐graph*

S3 S1 OR S2

S2 TX inflammatory bowel disease* or IBD or crohn* or colitis or regional enteritis or proctocolitis or colorectitis

S1 (MH "Inflammatory Bowel Diseases+")

Note: line S16: CINAHL filter for treatment studies Wong 2006, [Table 3] Best sensitivity, Ovid format.

### Appendix 6. AMED Search strategy (via Ovid)

1. exp inflammatory bowel disease/
2. (inflammatory bowel disease* or IBD or UC or CD).tw.
3. crohn*.tw.
4. (colitis or regional enteritis or proctocolitis or colorectitis).tw.
5. or/1‐4
6. (phone* or phoning or telephone* or telecom or telecommunicat* or tele‐communicat* or teleconferenc* or tele‐conferenc* or telegraph* or tele‐graph*).tw.
7. exp Telecommunications/
8. (Electronic Mail* or email* or e‐mail* or Telefacsimile or fax or telehealth or tele‐health or telemed* or tele‐med* or ehealth or e‐health or mhealth or m‐health).tw.
9. (instant messag* or SMS or text or texting).tw.
10. (webcast* or webina* or virtual conferenc*).tw.
11. ((web or internet or online or video or virtual or mobile or digital*) adj5 (care or communicat* or health* or medicine* or medical or clinic* or physician* or treat* or therap* or intervention* or conferenc* or connect*)).tw.
12. (mobile or hotline or videoconferenc* or wireless).tw.
13. (remote* adj5 (care or communicat* or health* or medicine* or medical or clinic* or physician* or treat* or therap* or intervention* or conferenc* or connect*)).tw.
14. (GoToMeeting or GoToWebinar or zoom meeting or spotMe or TurboMeeting or Livestorm).tw.
15. (Google Meet* or Cisco Webex or Microsoft Teams or join*me).tw.
16. or/6‐15
17. 5 and 16

### Appendix 7. Clinicaltrials.gov Search strategy

Advanced search:

Condition or disease: inflammatory bowel disease OR IBD OR ulcerative colitis OR Crohn OR Crohn's or Crohns

Intervention/treatment: remote care OR telemed OR tele‐med OR tele‐medicine OR tele‐medical OR telehealth OR tele‐health OR telecom OR telecommunication OR tele‐communication OR ehealth OR e‐health

Study type: Interventional studies (Clinical trials)

### Appendix 8. WHO ICTRP Search strategy

Advanced search:

(inflammatory bowel disease* or IBD or ulcerative colitis or crohn*) AND (remote* or tele* or phone* or ehealth* or e‐health*)

Recruitment status: All

## Data and analyses

**Comparison 1:** Web‐based disease monitoring versus usual care

Outcome or subgroup title	No. of studies	No. of participants	Statistical method	Effect size
1.1 Disease activity (adults)	3	428	Std. Mean Difference (IV, Random, 95% CI)	0.09 [‐0.11, 0.29]
1.1.1 Crohn's disease	2	273	Std. Mean Difference (IV, Random, 95% CI)	0.03 [‐0.21, 0.28]
1.1.2 Ulcerative colitis	3	155	Std. Mean Difference (IV, Random, 95% CI)	0.19 [‐0.13, 0.52]
1.2 Disease activity (adults; fixed‐effect sensitivity analysis)	3	428	Std. Mean Difference (IV, Fixed, 95% CI)	0.09 [‐0.11, 0.29]
1.2.1 Crohn's disease	2	273	Std. Mean Difference (IV, Fixed, 95% CI)	0.03 [‐0.21, 0.28]
1.2.2 Ulcerative colitis	3	155	Std. Mean Difference (IV, Fixed, 95% CI)	0.19 [‐0.13, 0.52]
1.3 Flare‐ups/relapse (dichotomous; adults)	5	868	Risk Ratio (M‐H, Random, 95% CI)	1.09 [0.93, 1.27]
1.3.1 Mixed inflammatory bowel disease	1	42	Risk Ratio (M‐H, Random, 95% CI)	1.17 [0.47, 2.89]
1.3.2 Crohn's disease	2	309	Risk Ratio (M‐H, Random, 95% CI)	1.12 [0.73, 1.71]
1.3.3 Ulcerative colitis	4	517	Risk Ratio (M‐H, Random, 95% CI)	1.08 [0.90, 1.30]
1.4 Flare‐ups/relapse (dichotomous; adults; fixed‐effect sensitivity analysis)	5	868	Risk Ratio (M‐H, Fixed, 95% CI)	1.10 [0.94, 1.29]
1.4.1 Mixed inflammatory bowel disease	1	42	Risk Ratio (M‐H, Fixed, 95% CI)	1.17 [0.47, 2.89]
1.4.2 Crohn's disease	2	309	Risk Ratio (M‐H, Fixed, 95% CI)	1.11 [0.81, 1.52]
1.4.3 Ulcerative colitis	4	517	Risk Ratio (M‐H, Fixed, 95% CI)	1.09 [0.91, 1.32]
1.5 Flare‐ups (continuous; adults)	1	909	Mean Difference (IV, Random, 95% CI)	0.00 [‐0.06, 0.06]
1.6 Flare‐ups/relapse (dichotomous; children)	1	170	Risk Ratio (M‐H, Random, 95% CI)	0.99 [0.65, 1.51]
1.6.1 Mixed inflammatory bowel disease	1	170	Risk Ratio (M‐H, Random, 95% CI)	0.99 [0.65, 1.51]
1.7 Quality of life (adults)	4	1099	Std. Mean Difference (IV, Random, 95% CI)	0.08 [‐0.04, 0.20]
1.7.1 Mixed inflammatory bowel disease	2	971	Std. Mean Difference (IV, Random, 95% CI)	0.07 [‐0.06, 0.19]
1.7.2 Crohn's disease	1	70	Std. Mean Difference (IV, Random, 95% CI)	0.39 [‐0.09, 0.86]
1.7.3 Ulcerative colitis	2	58	Std. Mean Difference (IV, Random, 95% CI)	0.03 [‐0.62, 0.69]
1.8 Quality of life (adults; fixed‐effect sensitivity analysis)	4	1099	Std. Mean Difference (IV, Fixed, 95% CI)	0.08 [‐0.04, 0.20]
1.8.1 Mixed inflammatory bowel disease	2	971	Std. Mean Difference (IV, Fixed, 95% CI)	0.07 [‐0.06, 0.19]
1.8.2 Crohn's disease	1	70	Std. Mean Difference (IV, Fixed, 95% CI)	0.39 [‐0.09, 0.86]
1.8.3 Ulcerative colitis	2	58	Std. Mean Difference (IV, Fixed, 95% CI)	0.02 [‐0.50, 0.54]
1.9 Medication adherence (continuous; adults)	1	671	Mean Difference (IV, Random, 95% CI)	0.24 [0.01, 0.47]
1.10 Medication adherence (continuous; children)	1	33	Mean Difference (IV, Random, 95% CI)	0.00 [‐0.63, 0.63]
1.11 Medication adherence (dichotomous; adults)	2	89	Risk Ratio (M‐H, Random, 95% CI)	0.87 [0.62, 1.21]
1.11.1 Mixed inflammatory bowel syndrome	1	42	Risk Ratio (M‐H, Random, 95% CI)	0.86 [0.53, 1.38]
1.11.2 Ulcerative colitis	1	47	Risk Ratio (M‐H, Random, 95% CI)	0.88 [0.55, 1.41]

**Comparison 2:** Telephone‐based disease monitoring versus face‐to‐face monitoring

Outcome or subgroup title	No. of studies	No. of participants	Statistical method	Effect size
2.1 Flare‐ups/relapse (dichotomous; adults)	1	42	Risk Ratio (M‐H, Random, 95% CI)	1.17 [0.47, 2.89]
2.2 Flare‐ups/relapse (dichotomous; children)	1	86	Risk Ratio (M‐H, Random, 95% CI)	0.24 [0.03, 2.05]
2.3 Quality of life (children)	1	67	Mean Difference (IV, Random, 95% CI)	7.00 [‐0.29, 14.29]
2.4 Number of episodes accessing healthcare (one or more hospital admissions; children)	1	86	Risk Ratio (M‐H, Random, 95% CI)	0.95 [0.06, 14.77]
2.5 Medication adherence (adults)	1	42	Risk Ratio (M‐H, Random, 95% CI)	0.50 [0.25, 0.98]
2.6 Participant engagement (adults)	1	42	Risk Ratio (M‐H, Random, 95% CI)	1.05 [0.89, 1.25]
2.7 Rate of attendance/engagement with the intervention (scheduled consultations not cancelled; children)	1	76	Mean Difference (IV, Random, 95% CI)	‐0.50 [‐1.38, 0.38]
2.8 Rate of attendance/engagement with the intervention (missed consultations; children)	1	76	Mean Difference (IV, Random, 95% CI)	1.00 [0.48, 1.52]
2.9 Rate of attendance of interactions with health professionals (children)	1	86	Risk Ratio (M‐H, Random, 95% CI)	0.86 [0.74, 1.00]

## Characteristics of studies

### Characteristics of included studies [ordered by study ID]

**Table:** Akobeng 2015

***Study characteristics***		
Methods	**Study design:** prospective RCT **Study duration:** July 2010–June 2013 **Setting:** Royal Manchester Children's Hospital, Manchester, UK, a regional Paediatric Gastroenterology referral centre	
Participants	**State of disease at beginning of study:** all in remission **Disease type:** IG: UC or indeterminate colitis (n = 8), CD (n = 36) CG: UC or indeterminate colitis (n = 7), CD (n = 35) **Inclusion criteria:** Diagnosis of IBD by established clinical, endoscopic, histological, and radiological criteria Clinical remission, defined as an aPCDAI score ≤ 10 for people with CD, or PUCAI score ≤ 10 for those with UC and indeterminate colitis **Exclusion criteria:** Active disease (aPCDAI ≥ 15 or PUCAI ≥ 15) Unwillingness to provide informed consent **Age at beginning of study:** All participants: 8–16 years IG: median 13.9 years (IQR 12.1–15.9) CG: median 13.8 years (IQR 11.2–15.3) **Sex:** IG: 30 boys, 14 girls CG: 24 boys, 18 girls **Number randomised:** IG: 44 CG: 42 **Number reaching end of study:** IG: 27 CG: 28	
Interventions	**IG:** telephone consultations with gastroenterology doctor; parents and participants advised to be together for the appointment (as in face‐to‐face consultations) **CG:** routine appointments in hospital as usual	
Outcomes	**Duration of follow‐up**: 24 months **Primary outcomes as defined by study authors:** QoL at 12 months (measured by the IMPACT questionnaire) **Secondary outcomes as defined by study authors:** Participant and parent satisfaction with consultations (assessed with the Consultation Satisfaction Questionnaire (CSQ)) Number of disease relapses (defined by the aPCDAI or PUCAI) Anthropometric measures (BMI, height, and weight z‐scores) Number of hospital admissions Proportion of consultations attended Duration of consultations Costs to the UK National Health Service (NHS)	
Notes	**Funding source:** "The project was funded by Research for Patient Benefit Programme, UK National Institute for Health Research (grant number PB‐PG‐0408‐16218)." **Conflicts of interest:** "The authors report grants from Research for Patient Benefit Programme, UK National Institute for Health Research, during the conduct of the study" **Contact with study authors:** no emails sent	
***Risk of bias***		
**Bias**	**Authors' judgement**	**Support for judgement**
Random sequence generation (selection bias)	Low risk	Computer‐generated randomisation scheme.
Allocation concealment (selection bias)	Low risk	Quote: "The assignment schedule was held centrally and allocation was performed by staff of the hospital's pharmacy department independent from the trial team."
Blinding of participants and personnel (performance bias) Study level	High risk	Masking not possible because of the nature of the interventions.
Blinding of outcome assessment (detection bias) Study level	High risk	Masking not possible because of the nature of the interventions.
Incomplete outcome data (attrition bias) Study level	Low risk	Attrition and reasons balanced between the groups.
Selective reporting (reporting bias)	Low risk	The trial was registered. Reported outcomes match the protocol and methods section.
Other bias	Low risk	No baseline imbalance.

**Table:** Ankersen 2019

***Study characteristics***		
Methods	**Study design:** prospective, open‐label, 1:1 RCT **Study duration:** July 2015–August 2016 **Setting:** outpatient clinic at the Department of Gastroenterology, North Zealand University Hospital, Denmark	
Participants	**State of disease at beginning of study:** remission (SCCAI ≤ 2 or HBI < 5) or with mild‐to‐moderate disease activity (SCCAI 3–4 or HBI 5–16) **Disease type per IG/CG:** IG: UC or indeterminate colitis (n = 8), CD (n = 36) CG: UC or indeterminate colitis (n = 7), CD (n = 35) **Inclusion criteria:** Age ≥ 18 years or older IBD according to Copenhagen diagnostic criteria Use of any medical IBD therapy Remission (SCCAI ≤ 2 or HBI < 5) or mild‐to‐moderate disease activity (SCCAI 3–4 or HBI 5–16) Ability to speak Danish Having a smartphone **Exclusion criteria:** Unwillingness to provide informed consent **Age at beginning of study:** IG: mean 48.4 years (SD 16.0); mean age at diagnosis was 37.3 years (SD 14.9) CG: mean 44.9 years (SD 15.2); mean age at diagnosis was 32.0 years (SD 13.1) **Sex:** IG: 24 men (48%), 26 women (52%) CG: 26 men (50%), 26 women (50%) **Number randomised:** IG: 50 CG: 52 **Number reaching end of study:** IG 45 CG: 43	
Interventions	**IG:** mobile phone application Constant Care. If participants experienced a recurrence of disease visualised on constant care web application (web‐app), they should contact the electronic care (eCare) personnel by phone or via the personal web‐wall, for an early consultation to assess the need for treatment adjustment or diagnostic investigation. The eCare nurses performed daily web ward rounds in close collaboration with a medical doctor. **CG:** self‐screening every 3 months	
Outcomes	**Duration of follow‐up**: 1 year **Primary outcomes as defined by study authors:** 1‐year disease course (traffic light system based on HBI, SCCAI, FC and TIBS). 2 internal assessors characterised the individual disease courses as follows. Chronic continuous course: red throughout 1 year Chronic continuous course: yellow throughout 1 year Chronic continuous course: red and yellow throughout 1 year Continuous remission course: green throughout 1 year Intermittent course: green, yellow, and red throughout 1 year Intermittent course: green with a single relapse (yellow or red) throughout 1 year Relapse Disease‐related quality of life measured with the SIBDQ **Secondary outcomes as defined by study authors:** Medical adherence measured by a self‐assessment questionnaire (MARS)	
Notes	**Funding source:** not reported **Conflicts of interest:** "Ankersen DV has received grants from Ferring Pharmaceuticals, Crohn Colitis patient society Denmark, North Zealand University Hospital and nonfinancial support from Calpro AS; Weimers P has received grants from Ferring lægemidler and Tillotts Pharma AG as well as nonfinancial support from Janssen‐ Cilag A/S, Calpro AS, and Vifor Pharma Nordiska AB; Marker D has received non‐financial support from Calpro AS and Pharmacosmos; Bennedsen M has received other financial support from AbbVie, Tillotts, Takeda, MSD and Pfizer; Saboori S has received non‐financial support from Janssen‐Cilag and Salofalk; Paridaens K is an employee of Ferring Pharmaceuticals; Burisch J has received grants from AbbVie, Takeda, Tillotts Pharma and personal fees from AbbVie, Janssen‐Cilag, Celgene, Samsung Bioepis, MSD, Pfizer and Takeda; Munkholm P has none to declare." **Contact with study authors:** we emailed the study authors on 17 October 2021 but received no response.	
***Risk of bias***		
**Bias**	**Authors' judgement**	**Support for judgement**
Random sequence generation (selection bias)	Unclear risk	Quote: "Patients were randomized to either be screened for disease activity whenever they felt necessary (OD group) or scheduled to be screened every 3M". Comment: insufficient information to make a judgement and no response to email request for clarification.
Allocation concealment (selection bias)	Unclear risk	Insufficient information to make a judgement and no response to email request for clarification.
Blinding of participants and personnel (performance bias) Study level	High risk	Quote: "This study was a 1‐year open‐label randomized trial (1:1) of adult IBD patients using the constant care platform for self‐monitoring of disease activity."
Blinding of outcome assessment (detection bias) Study level	High risk	Quote: "This study was a 1‐year open‐label randomized trial (1:1) of adult IBD patients using the constant care platform for self‐monitoring of disease activity."
Incomplete outcome data (attrition bias) Study level	Low risk	Reasons for dropouts are stated and are balanced. Stated drop‐out number in intervention and control groups.
Selective reporting (reporting bias)	Unclear risk	Trial registration offers limited information on outcomes, though outcomes are reported with appropriate data and are as expected.
Other bias	Low risk	No baseline imbalance or other sources apparent.

**Table:** Atreja 2018

***Study characteristics***		
Methods	**Study design:** prospective phase III, single‐centre, pragmatic RCT **Study duration:** 2 years (104 weeks), protocol registration date 18 February 2015 **Setting:** recruitment in outpatient and inpatient facilities in Mount Sinai Health System, NY, USA	
Participants	**State of disease at beginning of study:** insufficient information in abstract and protocol **Disease type:** mixed **Inclusion criteria:** Age ≥ 18 years Having a mobile phone or access to the internet at home Ability to complete a web‐based questionnaire in English **Exclusion criteria:** Inability to communicate with the investigators and comply with the study requirements Short bowel syndrome or stoma A condition or disease that, in the opinion of the investigators, may make it difficult for the person to use the HealthPROMISE app (e.g. advanced dementia) **Age at beginning of study:** adults **Sex**: 163 men (50.9%), 157 women (49.1%) **Number randomised:** IG: 162 CG: 158 **Number reaching end of study:** 315 (total)	
Interventions	**IG**: HealthPROMISE app: participants track QoL and symptoms every 2 weeks, providers use the visual data to improve care **CG:** usual care + IBD education app	
Outcomes	**Duration of follow‐up**: 104 weeks **Primary outcomes as defined by study authors:** Improvement in quality indicators from AGA outpatient quality metrics **Secondary outcomes as defined by study authors:** SIBDQ Emergency visits and hospitalisations Change in generic QoL score (EQ‐5D) Predictors of HEALTHPROMISE app utilisation	
Notes	**Funding source:** "The app was developed in‐house at Sinai AppLab. The study is supported by the Crohn's & Colitis Foundation of America (grant #253624) and the National Institutes of Health (5K23 DK97451‐02) with Ashish Atreja as the principal investigator." **Conflicts of interest:** not reported **Contact with study authors:** we sent emails for further clarification on 20 January 2021 and on 6 July 2021. The authors responded that the manuscript was under preparation for publication, providing no further clarification.	
***Risk of bias***		
**Bias**	**Authors' judgement**	**Support for judgement**
Random sequence generation (selection bias)	Unclear risk	Insufficient information in abstract and protocol to make a judgement.
Allocation concealment (selection bias)	Unclear risk	Insufficient information in abstract and protocol to make a judgement.
Blinding of participants and personnel (performance bias) Study level	Unclear risk	Insufficient information in abstract and protocol to make a judgement.
Blinding of outcome assessment (detection bias) Study level	Unclear risk	Insufficient information in abstract and protocol to make a judgement.
Incomplete outcome data (attrition bias) Study level	Unclear risk	Insufficient information in abstract and protocol to make a judgement.
Selective reporting (reporting bias)	Unclear risk	The trial was registered. Insufficient information to make a judgement as not all outcomes had been published at the time of the review.
Other bias	Unclear risk	Insufficient information in abstract and protocol to make a judgement.

**Table:** Carlsen 2017a

***Study characteristics***		
Methods	**Study design:** prospective, open‐label, 1:1 RCT **Study duration:** 2 years **Setting:** outpatient clinic at the Pediatric Department, Hvidovre University Hospital, Denmark	
Participants	**State of disease at beginning of study:** IG: UC in remission (n = 14), mild UC (n = 5); CD in remission (n = 2), mild CD (n = 5), moderate CD (n = 0), severe CD (n = 1) CG: UC in remission (n = 9), mild UC (n = 4); CD in remission (n = 5), mild CD (n = 6), moderate CD (n = 2), severe CD (n = 0) **Disease type:** IG: CD (n = 8), UC (n = 19) CG: CD (n = 13), UC (n = 13) **Inclusion criteria:** IBD diagnosis according to Copenhagen and Porto criteria Age 10–17 years Proficiency in Danish Access to the internet Nonbiological treatment (oral or topical) or no treatment for IBD **Exclusion criteria:** Insufficient Danish language skills Lack of intellectual capacity Growth retardation No access to the internet Biological treatment for IBD **Age at beginning of study:** IG: mean 15.1 years (SD 1.82) CG: mean 14.7 years (SD 2.11) **Sex:** IG: 10 boys, 17 girls CG: 12 boys, 14 girls **Number randomised:** IG: 27 CG: 26 **Number reaching end of study:** IG: 15 CG: 18	
Interventions	**IG**: paediatric/adolescent version of eHealth web‐based monitoring tool. Traffic light system based on self‐reported symptoms and FC. Paediatric QoL, school absence, and weight and height measures were added. A message tool was available for participants to write to the IBD team for non‐urgent matters. **CG:** hospital's IBD care guidelines (standard IBD care in Denmark), with outpatient visits every third month, including blood samples and FC. In addition, participant‐completed MARS and VAS, PUCAI/aPCDAI, days of school absence since last visit, and IMPACT III questionnaires.	
Outcomes	**Duration of follow‐up**: 2 years **Primary outcomes as defined by study authors:** Disease activity (self‐reported symptoms using PUCAI or aPCDAI) Relapse according to PUCAI and aPCDAI Health‐related QoL measured with IMPACT III Absence from school **Secondary outcomes as defined by study authors:** Total number of outpatient visits Medical adherence according to MARS Evaluation and adherence to the eHealth programme (number of entries of symptom scores and FC samples) Socioeconomic perspectives (reduced school absence and fewer outpatient visits)	
Notes	**Funding source:** "European Crohn's and Colitis Organization, Queen Louise's Hospital Foundation, TrygFoundation, CALPRO A/S, Tillotts Pharma, Capital Region Denmark, Alice and Frimodts Foundation, Ulcerative Colitis and Crohn’s Danish Patient Society, and Merck Sharp and Dome" **Conflicts of interest:** "V. Wewer: Advisory Board, MSD Denmark. A. Paerregaard: Advisory Board Nestle; Speaker fee (2015) Abbvie. The remaining authors have no conflict of interest to disclose" **Contact with study authors:** no emails sent	
***Risk of bias***		
**Bias**	**Authors' judgement**	**Support for judgement**
Random sequence generation (selection bias)	Unclear risk	Quote: "Patients were consecutively randomised by closed envelopes representing one of the 2 groups."
Allocation concealment (selection bias)	Low risk	Envelopes handled by a person not involved in the study group and blinded to the person enrolling patients.
Blinding of participants and personnel (performance bias) Study level	High risk	Open‐label study.
Blinding of outcome assessment (detection bias) Study level	High risk	Open‐label study.
Incomplete outcome data (attrition bias) Study level	Low risk	The drop‐out rate reported in the published paper is 20/53 (IG: 12/27 (44%); CG: 8/26 (31%)). There are no major differences and the reasons for drop‐outs are stated and are balanced. In the trial registration, enrolment is stated as 103 (IG: 56; CG: 47), but this seems to include a separate population of people in treatment with biological infusions.
Selective reporting (reporting bias)	High risk	The trial was registered. There is a difference in prioritisation of outcomes between the protocol (medication adherence) and published manuscript (disease activity). Disease activity and QoL not appropriately reported.
Other bias	Low risk	No baseline differences reported by study authors, but differences of PCDAI and PUCAI in remission between groups at baseline.

**Table:** Chauhan 2016

***Study characteristics***		
Methods	**Study design:** prospective RCT **Study duration:** 6 months **Setting:** outpatient clinic at McMaster Medical Centre, Canada	
Participants	**State of disease at beginning of study:** not reported **Disease type:** mixed, no further information provided **Inclusion criteria** People with IBD assigned 3 months after their current appointment **Exclusion criteria:** not reported **Age at beginning of study:** not reported **Sex:** not reported **Number randomised:** 60 in total, not reported per IG/CG **Number reaching end of study:** not reported	
Interventions	**IG**: telephone follow‐up visit by an IBD nurse practitioner 3 months after participant's current appointment **CG:** clinic follow‐up visit by an IBD nurse practitioner	
Outcomes	**Duration of follow‐up**: 6 months **Primary outcomes as defined by study authors:** Disease activity: CRP, HBI (CD) or Partial Mayo Score (UC) Health‐related QoL using SIBDQ **Secondary outcomes as defined by study authors:** Change in disease activity Participant satisfaction using Patient Satisfaction Questionnaire	
Notes	**Funding source:** not reported **Conflicts of interest:** not reported **Contact with study authors:** we send an email on 10 October 2021 and the study authors responded. The trial was under review in the journal, but we adjusted the risk of bias section with the results provided.	
***Risk of bias***		
**Bias**	**Authors' judgement**	**Support for judgement**
Random sequence generation (selection bias)	Low risk	Participants randomised 1:1 using a computer‐generated randomisation list and sealed envelopes.
Allocation concealment (selection bias)	Low risk	Participants randomised 1:1 using a computer‐generated randomisation list and sealed envelopes.
Blinding of participants and personnel (performance bias) Study level	High risk	Quote: "The participants and investigators were blinded using the sealed envelopes numbered chronologically for every participant (i.e. patient 001, patient 002, etc.). These sealed envelopes contained the treatment allocations (telephone follow‐up or clinic follow‐up) and were produced by a colleague researcher who was not involved in this study. This blinding of participants and investigators was maintained up until the participants have consented. Upon consenting, the corresponding sealed envelope was opened, and the participant and investigators became aware of the group allocation."
Blinding of outcome assessment (detection bias) Study level	High risk	Quote: "Upon consenting, the corresponding sealed envelope was opened, and the participant and investigators became aware of the group allocation."
Incomplete outcome data (attrition bias) Study level	Low risk	Reasons for dropouts stated, and dropout rate and reasons evenly distributed between the groups.
Selective reporting (reporting bias)	Unclear risk	Study authors state "We reported all primary and secondary outcomes as per our ethics approved study protocol"; however, the protocol is not available, and the trial was not registered.
Other bias	Low risk	More people with CD than with UC. Remaining baseline information was equal.

**Table:** Cross 2012

***Study characteristics***		
Methods	**Study design:** prospective RCT **Study duration:** November 2007–February 2010 **Setting:** University of Maryland, Baltimore, and the gastroenterology clinic of the Veterans Affairs, Maryland Heath Care System (VAMHCS), MD, USA	
Participants	**State of disease at beginning of study:** IG: active UC 40%, UC in remission 60% CG: active UC 32%, UC in remission 68% **Disease type:** UC **Inclusion criteria:** UC diagnosis confirmed by standard clinical, endoscopic, and histologic criteria Age ≥ 18 years **Exclusion criteria:** Inability to comply with study protocol Previous colectomy with ileostomy or colectomy with ileoanal anastomosis History of colonic dysplasia or colorectal cancer Uncontrolled medical or psychiatric disease Inability or unwillingness to provide consent Age < 18 years Other forms of colitis **Age at beginning of study:** IG: mean 41.7 years (SD13.9) CG: mean 40.3 years (SD 14.4) Overall: mean 41.1 years (SD14.0) **Sex:** IG: 10 men, 15 women CG: 7 men, 15 women **Number randomised:** IG: 25 CG: 22 **Number reaching end of study:** IG: 14 CG: 18	
Interventions	**IG**: home telemanagement in UC (UC HAT, comprising a home unit, a decision support server, and a web‐based clinician portal) **CG:** individualised written action plan at the time of group assignment without reinforcement, based on current evidence‐based guidelines and including scheduled and as‐needed clinic visits or calls, and educational fact sheets about UC	
Outcomes	**Duration of follow‐up**: 12 months **Primary outcomes as defined by study authors:** Clinical disease activity using Seo Index scores QoL using IBDQ **Secondary outcomes as defined by study authors:** Medication adherence using MMAS. To evaluate percentage adherence, the study authors dichotomised the variable (adherent/non‐adherent)	
Notes	**Funding source:** "Broad Medical Research Program (BRMP‐0190), University of Maryland General Clinical Research Center Grant (M01 RR16500), General Clinical Research Centers Program, National Center for Research Resources (NCRR), NIH, and the Baltimore Education and Research Foundation." **Conflicts of interest:** not reported **Contact with study authors**: we sent an email on 17 October 2021 and received additional information.	
***Risk of bias***		
**Bias**	**Authors' judgement**	**Support for judgement**
Random sequence generation (selection bias)	Low risk	Random permuted block design with randomly varied block sizes.
Allocation concealment (selection bias)	Low risk	Quote: "Group assignment was concealed and was not revealed to the patient or the research team members until after all baseline data were collected."; "We did computer randomization stratified by disease activity at enrollment (active or inactive). The group assignments were made using sealed envelopes."
Blinding of participants and personnel (performance bias) Study level	High risk	Participants not masked to their group assignments
Blinding of outcome assessment (detection bias) Study level	Low risk	Research staff at study visits blinded to treatment allocation of participants for subsequent visits.
Incomplete outcome data (attrition bias) Study level	High risk	8/22 (36.3%) children in the IG discontinued the intervention, compared to 1/19 (5.3%) in the CG.
Selective reporting (reporting bias)	Low risk	The trial was registered and the outcomes were appropriately presented.
Other bias	Low risk	IBDQ scores significantly higher at baseline in CG than in IG; however, this is of questionable clinical significance given the nature of the IBDQ system. No other imbalance.

**Table:** Cross 2019

***Study characteristics***		
Methods	**Study design:** prospective, 3‐arm, parallel RCT **Study duration:** September 2021–September 2016 **Setting:** University of Maryland, Baltimore, MD, USA; University of Pittsburgh, PA, USA; and Vanderbilt University, Nashville, TN, USA	
Participants	**State of disease at beginning of study:** IBD in remission (n = 200) and active IBD (n = 148) **Disease type:** IG1: CD (n = 79), UC (n = 36) IG2: CD (n = 78), UC (n = 38) CG: CD (n = 79), UC (n = 38) **Inclusion criteria: ** Age ≥ 18 years Diagnosis of CD, UC, or indeterminate colitis according to Lennard‐Jones classification ≥ 1 IBD flare‐up in 2 years prior to baseline visit (increase in IBD symptoms sufficient to warrant a change in medication dose or addition of a medication) **Exclusion criteria:** Inability to speak/read English Inability to comply with study protocol Presence of an ileostomy, colostomy, ileoanal pouch anastomosis, or ileorectal anastomosis Imminent surgery History of short bowel syndrome Uncontrolled medical/psychiatric disease Pregnancy Remission lasting ≥ 2 years **Age at beginning of study:** IG1: mean 40.1 years (SD 13.2) IG2 36.4 years (SD 11.5) CG 40.1 years (SD 11.7) **Sex: ** IG1: 48 men, 67 women IG2: 50 men, 66 women CG: 53 men, 64 women **Number randomised: ** IG1: 115 IG2: 116 CG: 117 **Number reaching end of study:** IG1 88 IG2 81 CG 90	
Interventions	**IG1**: participants log onto the TELE‐IBD website every other week to answer questions about disease symptoms, adherence, side effects, to check bodyweight and to receive educational content. Participants receive self‐action plans after each self‐testing session. Alerts are generated to the nurse co‐ordinator if certain clinical criteria are met. **IG2:** participants log onto the TELE‐IBD website weekly to answer questions about disease symptoms, adherence, side effects, to check bodyweight and to receive educational content. Participants receive self‐action plans after each self‐testing session. Alerts are generated to the nurse co‐ordinator if certain clinical criteria are met. **CG:** standard of care for participants modelled after the standard of care at all 3 study sites. Comprehensive assessment, a guideline concordant therapy plan, scheduled and as‐needed clinic visits, scheduled and as‐needed telephone calls, and administration of educational fact sheets about disease‐specific topics when appropriate.	
Outcomes	**Duration of follow‐up**: 12 months **Primary outcomes as defined by study authors:** Change in disease activity score and remission rates measures with HBI and SCCAI Change in disease‐specific QoL scores (IBDQ) **Secondary outcomes as defined by study authors:** Change in healthcare utilisation (number of hospitalisations, surgeries, emergency room visits, office visits, endoscopic procedures, non‐endoscopic procedures, IV therapeutics, non‐invasive diagnostic tests, electronic and telephone encounters)	
Notes	**Funding source:** Agency for Healthcare Research and Quality (1R01HS018975‐01A1) and the University of Maryland General Clinical Research Centers Program. **Conflicts of interest:** authors declared no conflict of interest **Contact with study authors:** we sent an email on 17 October 2021 and received additional information.	
***Risk of bias***		
**Bias**	**Authors' judgement**	**Support for judgement**
Random sequence generation (selection bias)	Low risk	Permuted block randomisation with randomly varied block sizes.
Allocation concealment (selection bias)	Low risk	Quote: "[...] the randomization arm assignments for each of the 4 (UC remission, UC active disease, CD remission, and CD active disease) strata were sent to the Cooperative Studies Program (CSP) Coordinating Center at the Veterans Affairs in Perry Point, MD, and entered into their interactive voice response system."
Blinding of participants and personnel (performance bias) Study level	High risk	Quote: "Investigators and staff were blinded to the randomization order, but patients, staff, and providers were not masked to group assignment."
Blinding of outcome assessment (detection bias) Study level	Low risk	Quote: "Investigators and staff were blinded to the randomization order, but patients, staff, and providers were not masked to group assignment." Comment: according to the trial registration (clinicaltrials.gov/ct2/show/NCT01692743) this study is single‐blind (outcome assessors) Response from authors: "The research staff was blind to the study group during the outcomes assessment."
Incomplete outcome data (attrition bias) Study level	Low risk	Balanced attrition and reasons for withdrawals thoroughly explained by the authors in our correspondence (27 October 2021). 48 participants in the intervention group discontinued and were accounted for in the published paper, while 42 participants were lost to follow‐up in the control group.
Selective reporting (reporting bias)	Low risk	In the 2015 published protocol for the study, there are more secondary outcomes than reported in the results. Most were reported in 3 publications referenced in this RCT, including all those relevant to this review. The outcomes match with the trial registration (NCT01692743).
Other bias	Low risk	No baseline imbalances.

**Table:** de Jong 2017

***Study characteristics***		
Methods	**Study design:** prospective RCT **Study duration:** July 2014–July 2016 **Setting:** 4 hospitals in the Netherlands: 2 academic hospitals (Maastricht University Medical Centre and Leiden University Medical Centre), and 2 large, non‐academic, regional hospitals (Zuyderland Medical Centre, Sittard, and St Antonius Hospital, Nieuwegein)	
Participants	**State of disease at beginning of study:** IG: in remission (n = 394), active (n = 71) CG: in remission (n = 380), active (n = 64) **Disease type:** IG: CD (n = 282), UC (n = 183) CG: CD (n = 262), UC (n = 182) **Inclusion criteria:** IBD diagnosis according to Lennard‐Jones criteria Age 18–75 years Access to internet by computer, tablet, or smartphone Dutch proficiency **Exclusion criteria:** Inability to read or understand the informed consent form Lack of internet access by computer, tablet, or smartphone Hospital admission within 2 weeks before inclusion Ileoanal pouch or ileorectal anastomosis **Age at beginning of study:** IG: mean 44.0 years (SD 14.1) CG: mean 44.1 years (SD 14.2) **Sex:** IG: 194 men, 271 women CG: 180 men, 264 women **Number randomised:** IG: 465 CG: 444 **Number reaching end of study:** IG: 438 CG: 443	
Interventions	**IG**: myIBDcoach is a secured webpage with an HTML application for tablet or smartphone and monthly monitoring modules, which contain questions regarding disease activity, medication use, treatment adherence, treatment satisfaction, and side effects, including infections. Also includes questions on factors affecting disease (including nutritional status, smoking, stress, life events, anxiety and depression, social support, physical exercise, and self‐management skills), and patient‐reported outcome measures on QoL and work productivity. **CG:** standard care with routine follow‐up visits according to the local protocol, with an opportunity to schedule an extra visit if symptoms relapsed.	
Outcomes	**Duration of follow‐up**: 12 months **Primary outcomes as defined by study authors:** Number of outpatient visits and telephone consultations with gastroenterologists and nurses Patient‐reported quality of care via VAS scores on patient satisfaction with healthcare, patients' experiences contacting their healthcare providers, and the extent to which healthcare meets patients' expectations **Secondary outcomes as defined by study authors:** Medication adherence measured with the 8‐item MMAS QoL measured with SIBDQ Self‐efficacy, defined as the perception of one's ability to engage in skills required to master a new challenge despite obstacles, measured with the 29‐item inflammatory bowel disease self‐efficacy scale (IBD‐SES) Disease‐related and medication‐related knowledge assessed on a VAS Smoking behaviour with a categorical question (non‐smoker, active‐smoker, or ex‐smoker) Numbers of relapses, defined as flares if symptoms suggestive of disease activity resulted in a dose escalation or initiation of a new drug to induce remission IBD‐related hospital admissions, emergency visits, surgeries, and corticosteroid use	
Notes	**Funding source:** academic incentive fund of the Maastricht University Medical Centre (31962340B) **Conflicts of interest:** Quote. "MJdJ reports non‐financial support from Merck Sharpe & Dohme, outside the submitted work. AEvdM‐dJ reports grants and non‐financial support from Takeda, personal fees from AbbVie, and non‐financial support from Tramedico, all outside the submitted work. AAvB reports personal fees from AbbVie, MSD, Ferring, Tramedico, Takeda, Pfizer, and Janssen, all outside the submitted work. GD reports speaker’s fees from Shire, AbbVie, and Takeda, and a grant for investigator‐initiated research from Takeda, all outside the submitted work. AAM reports grants from Grünenthal, Zon MW GGG (government), Will Pharma, BioActor, Pentax Europe, Falk Pharma, and Almiral Pharma, all outside the submitted work. AB received research grants to her department from AbbVie, Amgen, and Merck, and advisory board honoraria from Janssen and Sandoz, all unrelated to the current work. MJP reports personal fees from AbbVie, Ferring, Janssen, and Takeda, and grants from Falk, all outside the submitted work. All other authors declare no competing interests." **Contact with study authors:** we sent an email on 17 October 2021 and received additional information.	
***Risk of bias***		
**Bias**	**Authors' judgement**	**Support for judgement**
Random sequence generation (selection bias)	Low risk	Randomisation VIA ALEA Screening and Enrolment Application Software using the minimisation method, stratified for medical centre, IBS subtype (CD or UC), and treatment (no medication or Mesalazine; immunosuppressive drugs; or biological therapy).
Allocation concealment (selection bias)	Low risk	Enrolment via the software mentioned above.
Blinding of participants and personnel (performance bias) Study level	High risk	Open‐label study.
Blinding of outcome assessment (detection bias) Study level	High risk	Open‐label study.
Incomplete outcome data (attrition bias) Study level	Low risk	Attrition low in both groups. There were more reasons reported for dropping out of the IG (18/456) than the CG (1/444); however, this is unlikely to have affected the outcomes.
Selective reporting (reporting bias)	Low risk	Outcomes stated match the trial registration.
Other bias	Low risk	No baseline imbalances.

**Table:** Del Hoyo 2018

***Study characteristics***		
Methods	**Study design:** prospective, 3‐arm RCT **Study duration:** May 2014–December 2016 **Setting:** IBD Unit of La Fe University and Polytechnic Hospital (tertiary referral centre), Valencia, Spain	
Participants	**State of disease at beginning of study:** Remission: 30, based on the HBI and the partial Mayo scores. The other 33 apparently were not in remission. CD: IG1 6/ IG2 9/ CG 10 UC: IG1 2 / IG2 1 / CG 2 **Disease type:** IG1: CD (n = 13), UC (n = 8) IG2: CD (n = 13), UC (n = 8) CG: CD (n = 14), UC (n = 7) **Inclusion criteria:** Age ≥ 18 years IBD diagnosis according to internationally accepted criteria Initiation of therapy with corticosteroids, immunosuppressants, and biological agents due to disease activity Provision of written informed consent to participate in the study **Exclusion criteria:** Inability to speak and read Spanish Inability to manage a mobile phone or tablet or the internet, or not having a telephone line Participation in other clinical trials during the inclusion period Uncontrolled medical or psychiatric disease Presence of ileorectal or ileal pouch‐anal anastomosis Receipt of definitive ileostomy Perianal disease Pregnancy **Age at beginning of study:** IG1: median 40.91 years (range 24–60) IG2: median 41.32 years (range 19–66) CG: median 39.31 years (range 22–61) **Sex:** IG1: 12 men, 9 women 9 IG2: 9 men, 12 women CG: 12 men, 9 women **Number randomised:** IG1: 21 IG2: 21 CG: 21 **Number reaching end of study:** IG1 20 IG2 18 CG 19	
Interventions	**IG1**: nursing care by telephone: participants had periodic health status assessments delivered through structured interviews; clinical activity self‐recorded at home. Nurses modified medication or follow‐up schedule with support of medical staff according to results of the interview. **IG2:** Telemonitoring of CD and UC (TECCU): a web‐based telemanagement system with an http app (NOMHADhome) for mobile phones, tablets, and computers. Participants completed questionnaires on the platform related to symptoms and adverse effects. Alerts and action plans were established based on this information and the medical staff adjusted therapy accordingly. Through the platform, participants also received advice, reminders, educational material about their disease, and information on prevention. **CG:** usual care provided in the IBD Outpatient Clinic	
Outcomes	**Duration of follow‐up**: 24 weeks **Primary outcomes as defined by study authors:** Clinical remission of participants using HBI for CD and Mayo Index for UC **Secondary outcomes as defined by study authors:** Health‐related QoL measured by the generic EuroQol (EQ‐5D) questionnaire Health‐related QoL measured by the specific SIBDQ Participant satisfaction measured by a satisfaction questionnaire designed for the study Therapeutic adherence measured by the validated Morisky‐Green questionnaire Urgent and scheduled visits and urgent hospital admissions captured directly through hospital information system Number of surgical interventions related to the pathology Work activity and productivity measured by a validated questionnaire Mortality Directs health costs	
Notes	**Funding source:** "grants from the Instituto de Salud Carlos III‐Fondo de Investigaciones Sanitarias (FIS PI12/00277) and cofunded by FEDER (Fondo Europeo de Desarrollo Regional)" **Conflicts of interest:** "DD is the general manager of Connected Health Services." **Contact with study authors:** no emails sent	
***Risk of bias***		
**Bias**	**Authors' judgement**	**Support for judgement**
Random sequence generation (selection bias)	Low risk	Quote: "[...] block randomization method through a Web‐based tool [...] in order to generate a random‐allocation sequence and ensure allocation concealment."
Allocation concealment (selection bias)	Low risk	Quote: "[...] block randomization method through a Web‐based tool [...] in order to generate a random‐allocation sequence and ensure allocation concealment."
Blinding of participants and personnel (performance bias) Study level	High risk	Quote: "Neither the patients nor the researchers were masked to the intervention"
Blinding of outcome assessment (detection bias) Study level	High risk	Quote: "[...] the results were analyzed by an independent statistician who was blinded to group identification." Comment: However, all clinical outcome measures were analysed by staff who were not masked as per above.
Incomplete outcome data (attrition bias) Study level	Low risk	Low and balanced attrition and reasons for attrition.
Selective reporting (reporting bias)	Low risk	Outcomes stated match the trial registration.
Other bias	Low risk	No baseline imbalance.

**Table:** Elkjaer 2010a

***Study characteristics***		
Methods	**Study design:** prospective RCT **Study duration:** NR **Setting:** Herlev and Amager Hospitals, Copenhagen, Denmark; and Adelaide and Meath Hospital in Dublin, Ireland	
Participants	**State of disease at beginning of study:** SCCAI IG: Denmark: median 1 (range 0–10); Ireland: median 1 (range 0–9) CG: Denmark: median 1 (range 0–11); Ireland: median 2 (range 0–7) **Disease type:** UC **Inclusion criteria:** Age 18–69 years Mild/moderate UC diagnosed based on Copenhagen diagnostic criteria Treatment with 5‐ASA at 1 of the study centres **Exclusion criteria:** Acute phase of comorbid conditions (rheumatoid arthritis, chronic lung disease, coronary heart disease, chronic pancreatitis) Drug (narcotic) dependence or substance abuse Use of immunomodulators (azathioprine, 6‐mercaptopurine, metrothrexate or antitumour necrosis factor (TNF) therapy) Frequent treatment (> 6 months/year or 2 treatments/year) with high dose of systemic corticosteroids to enter remission Likely requirement of IBD surgery during the study period Previous IBD surgery Pregnancy or breastfeeding Inability to read or understand the informed consent form or use a computer **Age at beginning of study:** IG: Denmark: median 41 years (range 21–69); Ireland: median 42 years (range 18–68) CG: Denmark: median 48 years (range 21–69); Ireland: median 48 years (range 19–95) **Sex:** IG: Denmark: 57 men, 60 women; Ireland: 32 men, 20 women CG: Denmark: 35 men, 81 women; Ireland: 20 men, 28 women **Number randomised:** IG: Denmark: 117; Ireland: 52 CG: Denmark: 116; Ireland: 48 **Number reaching end of study:** IG: Denmark: 89; Ireland: 41 CG: Denmark: 97; Ireland: 38	
Interventions	**IG**: web‐based programme (www.constant‐care.dk). Participants who relapsed were requested to log on daily and complete the disease activity score (SCCAI) until they entered the green zone. In any event, they had to log on once a week until 4 weeks after the initiation of relapse. Participants were asked to fill in the SIBDQ at the beginning and the end of each relapse. Once remission was achieved, participants had to use the programme once a month until the next relapse occurred. **CG:** conventional treatment and follow‐up in the IBD outpatient clinic, including routine appointments or as‐needed appointments if participants were experiencing relapse symptoms. The attending physician evaluated the need for blood tests to monitor inflammation, and the need for sigmoideo‐ or colonoscopy. Participants who relapsed filled in the SCCAI and SIBDQ in paper format 7 days after remission and sent it to the investigator.	
Outcomes	**Duration of follow‐up**: 12 months **Primary outcomes as defined by study authors:** Compliance questionnaire with 5 questions: easy access to prescription, ability of relapse recognition, following the physician's advice, ability to self‐initiate acute treatment, and adherence to 5‐ASA treatment (5‐ASA refill compared with results from the e‐prescription pharmacy database) Disease outcome (SCCAI) IBD knowledge and QoL Disease specific QoL (SIBDQ) CCKNOW (multiple choice questionnaire) SF‐36 (Denmark) or SF‐12 (Ireland) HADS Safety (adverse events) Cost (number of outpatients visits, hospitalisation, and phone/online consultation) **Secondary outcomes as defined by study authors:** not reported	
Notes	**Funding source:** "PM is member of the advisory boards in Ferring, Tillots, MSD and Swedish Orphan. ME is member of the advisory board in Swedish Orphan. HS is member of the advisory board in Swedish Orphan. CO'M is on the International Advisory Board of Abbott, MSD, and Shire Pharmaceutical Company. He has unrestricted educational grants from Abbott and MSD" **Conflicts of interest: "**Colitis Crohn Patient Organisation, Moran’s Foundation, Vibeke Binder & Povl Riis’ Foundation, Bayer Health Care Funding, Augustinus Foundation, Munkholms Foundation, Tillotts Funding, Scientific Council at Herlev Hospital, Prof. Fagerhol Research Foundation, Aase & Einar Danielsen Foundation, Ole Trock‐Jansen & Hustrus Foundation, and European Crohn Colitis Organisation." **Contact with study authors:** we sent an email on 17 October 2021 but received no response.	
***Risk of bias***		
**Bias**	**Authors' judgement**	**Support for judgement**
Random sequence generation (selection bias)	Low risk	Quote: "Eligible patients, who had signed the informed consent form, were randomly allocated to the interventional (web) or to the control group by use of randomisation program"
Allocation concealment (selection bias)	Low risk	Quote: "Each randomisation number was placed in a closed, consecutively numbered envelope by two nurses not involved in the study."
Blinding of participants and personnel (performance bias) Study level	High risk	Quote: "This study was a 1‐year open‐label randomized trial (1:1) of adult IBD patients using the constant care platform for self‐monitoring of disease activity."
Blinding of outcome assessment (detection bias) Study level	High risk	Quote: "This study was a 1‐year open‐label randomized trial (1:1) of adult IBD patients using the constant care platform for self‐monitoring of disease activity."
Incomplete outcome data (attrition bias) Study level	Low risk	Balanced drop‐out rates and reasons for drop‐out reported for each group are balanced.
Selective reporting (reporting bias)	Unclear risk	No trial registration available, but all outcomes stated in the methods section are reported.
Other bias	Unclear risk	Significantly higher age and more women in CG (p < 0.05) in both groups with no explanation.

**Table:** Heida 2018

***Study characteristics***		
Methods	**Study design:** prospective RCT **Study duration:** April 2013–July 2016 **Setting:** 11 centres (6 tertiary care hospitals and 5 large regional general hospitals) in the Netherlands	
Participants	**State of disease at beginning of study:** remission **Disease type:** IG: UC (n = 45), CD (n = 39) CG: UC (n = 44), CD (n = 42) **Inclusion criteria:** Age 10–19 years IBD in clinical remission at baseline for ≥ 3 months, diagnosed according to Revised Porto criteria > 6 months before enrolment Access to a telephone, the internet, and an email address Good knowledge of the Dutch language **Exclusion criteria:** Treatment with anti‐TNF monoclonal antibodies Ileostomy or ileoanal pouch Any other comorbidity requiring frequent hospital visits **Age at beginning of study:** IG: median 15 years (range 12–16) CG: median 15 years (range 13–17) **Sex:** IG: 64 boys, 20 girls CG: 45 boys, 41 girls **Number randomised:** IG: 84 CG: 86 **Number reaching end of study:** IG: 48 CG: 72	
Interventions	**IG**: IBD‐live web app. Participants received automated email alerts to fill in the symptom score (PUCAI, PCDAI) and to send in a stool sample to the hospital laboratory; results were uploaded on the IBD‐live website and cumulated in a colour‐coded disease flare risk stratification that was visible to the participant and the local IBD team. **CG:** regular checks in the consultation room as before the trial, regardless of how well the participant was; the interval varied according to the physician's discretion.	
Outcomes	**Duration of follow‐up**: 52 weeks **Primary outcomes as defined by study authors:** Cumulative incidence of disease flares per group, defined as disease activity requiring therapy intensification (including steroid therapy, exclusive enteral nutrition, aminosalicylate dose escalation or introduction of anti‐TNF antibodies) **Secondary outcomes as defined by study authors:** Change in QoL measured with IBD‐specific IMPACT‐III questionnaire Cost‐effectiveness measured by direct and indirect medical and non‐medical costs Compliance to the home telemonitoring programme defined as being compliant to ≥ 80% of the alerts	
Notes	**Funding source:** "ZonMw Health Care Efficiency Research [grant number 837001001], Innovation Fund Dutch Insurance Companies [grant number B12‐204–2509], and NutsOhra Fund [grant number 1301‐002]. RKW is supported by the Netherlands Organization for Scientific Research [NWO] [grant number 016.136.308]. Reagents for the Quantum Blue® calprotectin point‐of‐care tests were an unrestricted donation by Bühlmann Laboratories AG. An unrestricted start‐up grant for the development of the web‐based programme IBD‐live was awarded by Ferring Pharmaceuticals BV. Neither funding company had a role in the design of this study, nor in the execution, analyses, interpretation of the data or decision to submit results." **Conflicts of interest: "**PFvR, AH and AMK received funding for joint research projects from BÜHLMANN Laboratories and CisBio Bioassays. All other authors had no support from any organization."	
***Risk of bias***		
**Bias**	**Authors' judgement**	**Support for judgement**
Random sequence generation (selection bias)	Low risk	Quote: "Computer generated random sequence 1:1 ratio stratified by research site and disease type [...]"
Allocation concealment (selection bias)	Low risk	Allocation concealment was ensured, as the study website did not release the randomisation code until the participant had been recruited into the trial.
Blinding of participants and personnel (performance bias) Study level	High risk	Quote: "The nature of the intervention did not allow blinding of participants, care providers or outcome assessors."
Blinding of outcome assessment (detection bias) Study level	High risk	Quote: "The nature of the intervention did not allow blinding of participants, care providers or outcome assessors."
Incomplete outcome data (attrition bias) Study level	Unclear risk	Imbalance in the participants reaching the end of the study; high number of non‐ or insufficient compliance in IG (36/84). Reason for non‐compliance not stated.
Selective reporting (reporting bias)	Low risk	Appropriate selection of outcomes that matches the trial registration.
Other bias	Low risk	Overrepresentation of males in IG compared with CG, but other characteristics were balanced and no other concerns.

**Table:** Hughes 2017

***Study characteristics***		
Methods	**Study design:** prospective RCT **Study duration:** not reported **Setting:** Hospital clinics (Guy's and St Thomas') and online through the Crohn's and Colitis UK website	
Participants	**State of disease at beginning of study:** not reported **Disease type:** unclear **Inclusion criteria:** IBD diagnosis Age > 18 years Ability to read and understand English fluently Informed consent **Exclusion criteria:** Suicidal ideations **Age at beginning of study:** IG: mean 38 years (SD 11.9) CG: mean 43 years (SD 13.7) **Sex:** IG: 13 men, 19 women CG: 10 men, 21 women **Number randomised:** IG: 32 CG: 31 **Number reaching end of study:** 54 (85%) in total (per group unclear)	
Interventions	**IG**: Quality Of LIfe Tool for IBD (QOLITI): cognitive‐behavioural therapy‐inspired manual providing information, guidance for setting goals for behaviour change, and accompanying tasks to aid implementation. to be completed at home in the participant's own time. Key themes are likely to include symptom management, dealing with social implications of the disease and interacting effectively with healthcare professionals. Participants also receive 30‐minute telephone support sessions with a healthcare professional, at 2, 4 and 6 weeks after randomisation. **CG:** waitlist: after study completion, the control group receive the same manual, but without telephone support sessions.	
Outcomes	**Duration of follow‐up**: 8 weeks **Primary outcomes as defined by study authors:** Acceptability Change in numbers of participants throughout the trial Feasibility Effectiveness Change in depression Change in anxiety Change in generic QoL Change in IBD‐specific QoL **Secondary outcomes as defined by study authors:** Retrospective appraisal of the intervention (i.e. content and layout) through semi‐structured qualitative interviews Change in fatigue Change in illness perception Change in disease activity	
Notes	**Funding source:** not reported **Conflicts of interest:** none **Contact with study authors:** we sent an email on 20 January 2021 but received no response.	
***Risk of bias***		
**Bias**	**Authors' judgement**	**Support for judgement**
Random sequence generation (selection bias)	Unclear risk	Insufficient information to make a decision.
Allocation concealment (selection bias)	Unclear risk	Insufficient information to make a decision.
Blinding of participants and personnel (performance bias) Study level	High risk	Type of study that cannot be blinded.
Blinding of outcome assessment (detection bias) Study level	Unclear risk	Single‐blind design (outcomes assessor) according to trial registration, but this is inconsistent with the methods reported. We wrote to the study authors for clarification but received no response.
Incomplete outcome data (attrition bias) Study level	Unclear risk	Insufficient information on withdrawals offered to judge and no response from study authors.
Selective reporting (reporting bias)	Unclear risk	Not all outcomes reported as per the trial registration.
Other bias	Low risk	No differences in baseline characteristics and no other concerns.

**Table:** Ley 2020

***Study characteristics***		
Methods	**Study design:** prospective RCT **Study duration:** not reported **Setting:** not reported	
Participants	**State of disease at beginning of study:** all in remission **Disease type:** UC **Inclusion criteria:** Age 18–65 years UC diagnosis Clinical remission Stable dose of 5‐ASA monotherapy for ≥ 2 months before study entry **Exclusion criteria:** not reported **Age at beginning of study:** IG: mean 38 years CG: mean 34.3 years **Sex: m/f IG: 14/7 CG: 11/7** IG: 14 men, 7 women CG: 11 men, 7 women **Number randomised:** IG: 21 CG: 18 **Number reaching end of study:** not reported	
Interventions	**IG**: iPhone adherence application that included medication reminders **CG:** sham application that included educational materials and the capability of recording medication intake, without medication reminders	
Outcomes	**Duration of follow‐up**: not reported **Primary outcomes as defined by study authors:** Medication adherence BMQ as a method of adherence prediction **Secondary outcomes as defined by study authors:** not reported	
Notes	**Funding source:** "research support from Takeda Pharmaceuticals" **Conflicts of interest:** not reported **Contact with study authors:** we sent an email on 17 October 2021 but received no response.	
***Risk of bias***		
**Bias**	**Authors' judgement**	**Support for judgement**
Random sequence generation (selection bias)	Unclear risk	Insufficient detail to make a decision.
Allocation concealment (selection bias)	Unclear risk	Insufficient detail to make a decision.
Blinding of participants and personnel (performance bias) Study level	Low risk	Double‐blind RCT.
Blinding of outcome assessment (detection bias) Study level	Unclear risk	Insufficient detail to make a decision.
Incomplete outcome data (attrition bias) Study level	Unclear risk	Insufficient detail to make a decision.
Selective reporting (reporting bias)	Unclear risk	Insufficient detail to make a decision.
Other bias	Low risk	No baseline imbalance apparent and no other concerns noted.

**Table:** Malickova 2020

***Study characteristics***		
Methods	**Study design:** RCT **Study duration:** June 2018–August 2019 **Setting:** Prague hospital, Czechia	
Participants	**State of disease at beginning of study:** all in remission **Disease type:** IG: CD (n = 44), UC (n = 46) CG: CD (n = 19), UC (n = 18) **Inclusion criteria:** Age > 18 years CD or UC diagnosis In remission (controlled by endoscopic examination implemented during the last 12 months before the start of the study) Computer literacy Regular access to PC, tablet, or smartphone Working email address Informed consent **Exclusion criteria:** Not reported **Age at beginning of study:** IG: median 43 years (IQR 28–56) CG: median 42 years (IQR 23–60) **Sex:** IG: 44 men, 46 women CG: 15 men, 22 women **Number randomised:** IG: 94 CG: 37 **Number reaching end of study:** IG: 90 CG: 37	
Interventions	**IG**: participants were telemonitored and connected with their doctors and IBD nurses through the IBD Assistant application, available online. They received email reminders at regular intervals to fill in standard electronic assessments. An emergency questionnaire, for use in case of deterioration, advised participants to contact a doctor. Participants contacted the doctor primarily through the IBD Assistant web application; in‐person visits were scheduled only after a recommendation via the IBD Assistant application. FC was measured at least 4 times/12 months with at‐home CalpoSmart system. **CG:** participants attended usual checkups every 3 months in outpatient clinics with their gastroenterologists (clinical examination and laboratory testing). Participants could have an unscheduled acute consultation in case of any difficulties, or an at‐home doctor's visit in the event of unfavourable examination results.	
Outcomes	**Duration of follow‐up**: 12 months **Primary outcomes as defined by study authors:** Outpatient visits Disease activity Inflammation markers Intercurrent infections Hospitalisations Costs **Secondary outcomes as defined by study authors:** not reported	
Notes	**Funding source:** "The study was supported by the IBD‐Comfort Foundation Fund and the Prevention Fund of the General Health Insurance Company of the Czech Republic." **Conflicts of interest:** Study authors declared no conflict of interest. **Contact with study authors:** we sent an email on 27 October 2021 for further clarification regarding risk of bias, and we received a response on 1 November 2021.	
***Risk of bias***		
**Bias**	**Authors' judgement**	**Support for judgement**
Random sequence generation (selection bias)	Low risk	Quote (personal correspondence): "Assignment to a telemedicine or control group was performed by a simple random allocation using a table of random numbers."
Allocation concealment (selection bias)	Unclear risk	Insufficient detail to make a decision.
Blinding of participants and personnel (performance bias) Study level	High risk	Type of study that cannot be blinded.
Blinding of outcome assessment (detection bias) Study level	Low risk	Quote (personal correspondence): "the evaluation of the objectives pursued was carried out without knowing which of the groups the entity belongs to."
Incomplete outcome data (attrition bias) Study level	Low risk	Low attrition, explained and balanced between groups.
Selective reporting (reporting bias)	Unclear risk	Outcomes reported as per the last paragraph of the introduction, but no trial registration and variances are missing.
Other bias	Unclear risk	Huge difference in numbers randomised (IG 90/CG 37) not explained in paper, but a study author provided clarification. Quote (personal correspondence): "Initially, a 3:1 split was considered, ie 90 subjects in the telemedicine and 30 subjects in the control branch. The final 90/37 ratio was due to a change in the randomization design."

**Table:** McCombie 2020

***Study characteristics***		
Methods	**Study design:** prospective RCT **Study duration:** August 2015–December 2016 **Setting:** Southern, Canterbury, Waitemata, and Hutt Valley District Health Boards across New Zealand	
Participants	**State of disease at beginning of study:** IG: SCCAI mean 1.6 (SD 2.5); HBI mean 2.7 (SD 3.00)* CG: SCCAI mean 1.1 (SD 1.5); HBI mean 2.7 (SD 3.0)* *SCCAI ≤ 2: remission, SCCAI ≤ 3: relapse (for UC); HBI ≤ 4: remission, HBI > 4: relapse (for CD) **Disease type:** IG: CD (n = 37), UC (n = 13) CG: CD (n = 36), UC (n = 14) **Inclusion criteria:** Age ≥ 16 years Confirmed UC or CD ≥ 2 outpatient appointments in the last 12 months < 3 disease flares in the past 12 months **Exclusion criteria:** Indeterminate colitis Severe disease with close monitoring Possible surgical intervention Previous surgery Pregnancy Ileostomy, colostomy, or ileal pouch–anal anastomosis Inability of unwillingness to provide written consent **Age at beginning of study:** IG: mean 35.2 years (SD 12.4) CG: mean 34.3 years (SD 12.9) **Sex:** IG: 26 men, 24 women CG: 23 men, 27 women **Number randomised:** IG: 53 CG: 54 **Number reaching end of study:** IG: 47 CG: 49	
Interventions	**IG**: IBDsmart and IBDoc apps. IBDsmart allowed participants to complete symptom scores and send them to their doctor. Participants could log in and fill out a questionnaire (CDAI or SCCAI), which produced a score indicating disease severity. In this way, the app tracked long‐term trends of symptom scores, and the healthcare team were contacted immediately in case of high disease severity. IBDoc allowed participants to measure their FC levels by testing stool samples with a medical device, and sending the results to their doctor via an app build into IBDoc called CalApp. **CG:** usual outpatient treatment	
Outcomes	**Duration of follow‐up**: 12 months **Primary outcomes as defined by study authors:** Noninferiority of IBDsmart and IBDoc to standard care **Secondary outcomes as defined by study authors:** Health‐related QoL at 3, 6, and 9 months Participant‐reported usability/acceptability Doctor‐reported usability/acceptability Adherence FC	
Notes	**Funding source:** "The Healthcare Otago Charitable Trust (no grant number) and The New Zealand Society of Gastroenterology Janssen Research Fellowship (no grant number) in 2015 and the gut health network, a research theme located at the Department of Medicine, University of Otago." **Conflicts of interest:** none **Contact with study authors:** we sent an email on 17 October 2021 and the study authors provided additional information.	
***Risk of bias***		
**Bias**	**Authors' judgement**	**Support for judgement**
Random sequence generation (selection bias)	Low risk	Quote: "Randomization occurred by a computer program randomly allocating participants to 1 of the 2 groups. Randomization was stratified by disease type (CD vs UC) and location of outpatient appointments (Waitemata, Hutt Valley, Canterbury, and Southern District Health Boards)."
Allocation concealment (selection bias)	Low risk	Quote: "The allocations were put in sequenced envelopes, which were to be opened by the recruiting nurse, gastroenterologist, or researcher."
Blinding of participants and personnel (performance bias) Study level	High risk	Quote: "Participants were not blinded to which group they were in."
Blinding of outcome assessment (detection bias) Study level	High risk	Masking not used.
Incomplete outcome data (attrition bias) Study level	Low risk	Low and balanced attrition: only 4 dropouts and there was no reason recorded except that participants had asked to withdraw.
Selective reporting (reporting bias)	Low risk	No major difference from the trial registration.
Other bias	Low risk	Some baseline data missing for participants who dropped out without completing baseline assessment, but no important differences.

**Table:** Reich 2019

***Study characteristics***		
Methods	**Study design:** prospective RCT **Study duration:** November 2017–March 2018 **Setting:** Boston Medical Center, Boston, MA, USA	
Participants	**State of disease at beginning of study:** IG: HBI (for CD): mean 4.6 (SD 3.8); SCCAI (for UC): mean 4.3 (SD 3.0) CG: HBI (for CD): mean 4.6 (SD 4.1); SCCAI (for UC): mean 4.3 (SD 2.8) **Disease type:** IG: CD (n = 36), UC (n = 28) CG: CD (n = 36), UC (n = 27) **Inclusion criteria:** Age ≥ 18 years Both sexes IBD diagnosis (CD, UC, or indeterminate colitis) by standard criteria Scheduled appointment at outpatient gastroenterology clinic or infusion unit **Exclusion criteria:** Inability to communicate in English Cognitive impairment that would impair participation Lack of access to computer with internet Expected move from study area during the study **Age at beginning of study:** IG: mean 41 years (SD 15.7) CG: mean 42 years (SD 16.4) **Sex:** IG: 35 men, 28 women (1 person missing) CG: 42 men, 21 women **Number randomised:** IG: 64 CG: 63 **Number reaching end of study:** IG: 46 CG: 29	
Interventions	**IG**: MyChart: a patient portal that allowed participants to see various parts of their medical record, and send and receive secure messages with their provider. Participants received educational information about IBD every 2 weeks along with reminders to take their medications and get vaccinated for influenza and pneumococcal pneumonia at 2 weeks and 3 months after enrolment. **CG:** participants were sent generic messages through MyChart that were not related to IBD (e.g. "Did you know that you could send your provider a message through MyChart if you need to refill a medication? Please contact your provider if you need your medications refilled.")	
Outcomes	**Duration of follow‐up**: 6 months **Primary outcomes as defined by study authors:** QoL (SIBDQ) **Secondary outcomes as defined by study authors:** MyChart portal satisfaction Vaccine uptake for influenza and pneumonia	
Notes	**Funding source:** "Supported by a generous gift from Aimee & Kleanthis Dendrinos and Robin & Andrew Davis." **Conflicts of interest:** none **Contact with study authors:** we sent an email on 21 January 2021 but received no response.	
***Risk of bias***		
**Bias**	**Authors' judgement**	**Support for judgement**
Random sequence generation (selection bias)	Unclear risk	Quote: "After baseline data were collected, subjects were randomized in a 1:1 ratio of experimental to control arm stratified by MyChart naïve/active status using a block size of two." Comments: randomisation method not provided; no response to our email sent on 21 January 2021.
Allocation concealment (selection bias)	Unclear risk	Insufficient information to make a decision.
Blinding of participants and personnel (performance bias) Study level	High risk	Due to the nature of a study, it is not possible to blind.
Blinding of outcome assessment (detection bias) Study level	Unclear risk	Insufficient information to make a decision; no response to our email sent on 21 January 2021.
Incomplete outcome data (attrition bias) Study level	Unclear risk	Higher attrition in the control group and imbalance was 28% attrition vs 56% – reason for all dropouts is "lost to follow up" with no further details. No response to our email sent on 21 January 2021.
Selective reporting (reporting bias)	Low risk	Time to referral for behavioural health not reported, but no other missing outcomes as prespecified in trial registration.
Other bias	Low risk	No concerns with baseline characteristics between groups.

**Table:** Siegel 2018

***Study characteristics***		
Methods	**Study design:** cluster‐RCT **Study duration:** 3 years **Setting:** 16 gastroenterology practices across the USA (8 academic, 8 community‐based)	
Participants	**State of disease at beginning of study:** not reported **Disease type:** CD **Inclusion criteria:** Age > 18 CD diagnosis within past 15 years of diagnosis No current or prior disease complications Not on immunomodulators or biologics but considered a candidate for these treatments **Exclusion criteria:** not reported **Age at beginning of study:** IG: median 32 years (range 18–69) CG: median 31 years (range 18–69) **Sex:** IG: 64 men, 69 women CG: 24 men, 45 women **Number randomised:** IG: 133 CG: 69 **Number reaching end of study:** not reported	
Interventions	**IG**: a decision aid including an online programme reviewing benefits and risks of treatment options combined with a personalised risk prediction tool (PROSPECT) for Crohn’s disease **CG:** standard of care	
Outcomes	**Duration of follow‐up**: 3 years **Primary outcomes as defined by study authors:** Choice of combination therapy **Secondary outcomes as defined by study authors:** Decision conflict Understanding of the disease	
Notes	**Funding source:** not reported **Conflicts of interest:** not reported **Contact with study authors:** we sent an email on 21 January 2021 but received no response.	
***Risk of bias***		
**Bias**	**Authors' judgement**	**Support for judgement**
Random sequence generation (selection bias)	Unclear risk	Insufficient information to make a decision.
Allocation concealment (selection bias)	Unclear risk	Insufficient information to make a decision.
Blinding of participants and personnel (performance bias) Study level	Unclear risk	Insufficient information to make a decision.
Blinding of outcome assessment (detection bias) Study level	Unclear risk	Insufficient information to make a decision.
Incomplete outcome data (attrition bias) Study level	Unclear risk	Insufficient information to make a decision.
Selective reporting (reporting bias)	Unclear risk	Insufficient information to make a decision.
Other bias	Low risk	Demographics were similar between groups, with more women in the control group and slightly shorter disease duration in the intervention group.

**Table:** Stunkel 2012

***Study characteristics***		
Methods	**Study design:** prospective RCT **Study duration:** 38 weeks **Setting:** remote, conducted in USA	
Participants	**State of disease at beginning of study:** mild to moderate **Disease type:** not reported **Inclusion criteria:** IBD diagnosis Access to a smart device (iPhone/iPad/iPod touch or Android). **Exclusion criteria:** Blackberry smartphones (app not fully optimised for this device) **Age at beginning of study:** 20–84 years **Sex:** 44 men, 46 women **Number randomised:** 90 **Number reaching end of study:** not reported	
Interventions	**IG**: daily use of app (WellApps, New York, NY) to record symptoms, track pain, stress levels, frequency, and quality of bowel movements **CG:** education about websites such as www.ccfa.org for information on IBD	
Outcomes	**Duration of follow‐up**: 38 weeks **Primary outcomes as defined by study authors:** Background information Change in QoL Time to follow‐up Participant satisfaction **Secondary outcomes as defined by study authors:** not reported	
Notes	**Funding source:** not reported **Conflicts of interest:** not reported **Contact with study authors:** we sent an email on 17 October 2021 but received no response.	
***Risk of bias***		
**Bias**	**Authors' judgement**	**Support for judgement**
Random sequence generation (selection bias)	Unclear risk	Insufficient information to make a decision.
Allocation concealment (selection bias)	Unclear risk	Insufficient information to make a decision.
Blinding of participants and personnel (performance bias) Study level	Unclear risk	Insufficient information to make a decision.
Blinding of outcome assessment (detection bias) Study level	Unclear risk	Insufficient information to make a decision.
Incomplete outcome data (attrition bias) Study level	Unclear risk	Insufficient information to make a decision.
Selective reporting (reporting bias)	Unclear risk	Insufficient information to make a decision.
Other bias	Unclear risk	Insufficient information to make a decision.

**Table:** Wang 2020

***Study characteristics***		
Methods	**Study design:** not reported **Study duration:** May 2016–April 2018 **Setting:** remote (through General Hospital of the Eastern Theater, China)	
Participants	**State of disease at beginning of study:** postoperative CD IG: 33 people with active disease CG: 39 people with active disease **Disease type:** CD **Inclusion criteria: ** Age 18–65 years CD diagnosis based on clinical, imaging, and endoscopy screening, > 6 months prior to study entry than 6 months CD‐related surgical treatment No allergies or contraindications to azathioprine (given to participants for postoperative maintenance treatment) Ability to read and browse information Fluency in use of WeChat app Voluntary participation **Exclusion criteria:** Current use of other drugs as maintenance treatment Cognitive impairment Illiteracy or other language or communication impairment Inability to use WeChat app Current participation in other research or psychological interventions **Age at beginning of study:** IG: mean 32.46 years (SD 10.11) CG: mean 33.85 years (SD 11.2) **Sex:** IG: 57 men, 63 women CG: 59 men, 60 women **Number randomised:** IG: 120 CG: 119 **Number reaching end of study:** IG: 101 CG: 96	
Interventions	**IG**: WeChat platform: a drug self‐management platform based on the 5 key points of self‐management theory (self‐cognition, goal‐setting, self‐monitoring, self‐motivation, and self‐evaluation), implemented using WeChat. **CG:** regular health education and guidance on drugs by designated nurses during inpatient stay. Participants provided with a brochure with drug guidance upon discharge. The content of the brochure included basic knowledge of drugs, drug usage and effects, how to deal with common problems, and how to attend follow‐ups in outpatient clinic. Doctors provided follow‐up guidance every 2 months.	
Outcomes	**Duration of follow‐up:** 6 months **Primary outcomes as defined by study authors:** Drug adherence (MMAS‐8 score) Proportion and duration of relapses **Secondary outcomes as defined by study authors:** Azathioprine metabolites FC levels	
Notes	**Funding source:** Nursing Project of Military Medical Science and Technology Youth Cultivation Plan, No. 19QNP077 **Conflicts of interest:** not reported **Contact with study authors:** we did not send an email owing to the language barrier. We translated this study using an online translator.	
***Risk of bias***		
**Bias**	**Authors' judgement**	**Support for judgement**
Random sequence generation (selection bias)	Low risk	Computer‐generated.
Allocation concealment (selection bias)	Low risk	Sealed envelopes.
Blinding of participants and personnel (performance bias) Study level	High risk	Open‐label study.
Blinding of outcome assessment (detection bias) Study level	Unclear risk	Not mentioned.
Incomplete outcome data (attrition bias) Study level	Low risk	Low and balanced attrition and reasons.
Selective reporting (reporting bias)	Unclear risk	No trial registration. The outcomes specified in the method section are poorly reported, especially relapses.
Other bias	Low risk	No baseline imbalances between groups.

5‐ASA: 5‐aminosalicylic acid; AGA: American Gastroenterological Association; aPCDAI: abbreviated Paediatric Crohn's Disease Activity Index; BMI: body mass index; BMQ: Brief Medication Questionnaire; CCKNOW: Crohn's and Colitis Knowledge; CD: Crohn's disease; CDAI: Crohn's Disease Activity Index; CG: control group; CRP: C‐reactive protein; EQ‐5D: EuroQol five‐dimension questionnaire; FC: faecal calprotectin; HADS: Hospital Anxiety and Depression Scale; HBI: Harvey Bradshaw Index; IBD: inflammatory bowel disease; IBDQ: Inflammatory Bowel Disease Questionnaire; IG: intervention group; IQR: interquartile range; MARS: Medication Adherence Report Scale; MMAS: Morisky Medication Adherence Scale; PCDAI: Pediatric Crohn's Disease Activity Index; PUCAI: Paediatric Ulcerative Colitis Activity Index; QoL: quality of life; RCT: randomised controlled trial; SCCAI: Simple Clinical Colitis Activity Index; SD: standard deviation; SF‐12/36: Medical Outcomes Study 12/36‐item Short‐Form Health Survey; SIBDQ: Short Inflammatory Bowel Disease Questionnaire; TIBS: Total Inflammatory Burden Score; TNF: tumour necrosis factor; UC: ulcerative colitis; VAS: visual analogue scale.

### Characteristics of excluded studies [ordered by study ID]

**Table:**

Study	Reason for exclusion
Ankersen 2017	Wrong intervention.
Camba 2013	Wrong study design.
Carlsen 2017b	Wrong intervention (scheduling infliximab infusions).
Creed 2019	Wrong study design.
Del Hoyo 2021	Wrong study design.
Elkjaer 2010b	Wrong intervention.
Gray 2020	Wrong study design.
Greenley 2015	Wrong study design (participating youth were recruited sequentially from 1 of 2 paediatric IBD centres in the Midwest region of the USA).
Jambaulikar 2015	Wrong intervention.
Krier 2011	Wrong study design (blinded administrative staff randomly scheduled clinic appointments to newly established patients).
Mastronardi 2020	Wrong study design.
Miloh 2017	Wrong study design (participants served as their own controls; information provided by study author).
Moss 2010	Wrong study design.
NCT00310362	Wrong population.
NCT01852097	Wrong intervention.
NCT02265588	Wrong intervention.
NCT02707068	Wrong intervention.
NCT03486158	Wrong intervention.
NCT03695783	Wrong intervention.
NCT04151420	Wrong study design.
NCT04165265	Wrong study design.
Oser 2018	Wrong intervention.
RBR‐79dn4k	Wrong intervention.
Snoei 2009	Wrong study design.
Sutton 2019	Wrong intervention.
Tripp 2017	Wrong intervention.
Zhang 2020	Wrong intervention.

IBD: inflammatory bowel disease.

### Characteristics of studies awaiting classification [ordered by study ID]

**Table:** Bonnaud 2021

Methods	RCT
Participants	54
Interventions	IG: EasyMICI–MaMICI telemedicine platform CG: standard care
Outcomes	Primary outcomes: Efficacy of the software platform, as measured by QoL and quality of care. Secondary outcomes: Changes in the use of healthcare resources Patient satisfaction in the MaMICI group
Notes	We identified this study during our update search, and we will include it in the next update of this review.

**Table:** Hommel 2015

Methods	RCT
Participants	140
Interventions	IG: Telehealth Behavioral Treatment CG: education
Outcomes	Primary outcomes: Medication adherence Secondary outcomes: Health‐related QoL Disease severity Healthcare utilisation
Notes	We contacted the study authors on 21 January 2021 but received no response.

**Table:** NCT02085083

Methods	RCT
Participants	150
Interventions	IG: regular telephone and email access to an IBD nurse CG: minimal Intervention
Outcomes	Primary outcomes: Patient satisfaction Medication adherence Healthcare utilisation Transition readiness Secondary outcomes: QoL Disease activity Disease knowledge
Notes	We contacted the study authors on 21 January 2021 but received no response.

**Table:** NCT02694042

Methods	RCT
Participants	39
Interventions	IG: Mission is Remission Group CG: no intervention
Outcomes	Primary outcomes: Self‐efficacy Health‐related QoL Secondary outcomes: Medication taking behaviour Disease activity Disease knowledge Physical and social activity participation Transition readiness
Notes	We contacted the study authors on 21 January 2021 but received no response.

**Table:** NCT03059186

Methods	RCT
Participants	129
Interventions	IG: online daily gratitude journal CG: no intervention
Outcomes	Primary outcomes: Depression and anxiety Disease activity Secondary outcomes: Self‐efficacy Gratitude Emotion regulation
Notes	We contacted the study authors on 21 January 2021; they told us the study was not yet published.

**Table:** NCT03186872

Methods	RCT
Participants	90
Interventions	IG: digital behavioural programme app CG: no intervention
Outcomes	Primary outcomes: Anxiety Secondary outcomes: Depression
Notes	We contacted the study authors on 21 January 2021 but received no response.

**Table:** NCT04754620

Methods	RCT
Participants	139
Interventions	IG: online visit by a smartphone application CG: standard face‐to‐face visit
Outcomes	Primary outcomes: Satisfaction score with the video visits Secondary outcomes: not reported
Notes	We will include this study in an update of this review.

**Table:** NTR2892

Methods	RCT
Participants	211
Interventions	IG: nurse‐based intervention CG: patient‐centred (eHealth) intervention
Outcomes	Primary outcomes: Information recall Medication adherence Secondary outcomes: Nurse‐patient communication Current levels of generalised anxiety Psychological distress
Notes	We contacted the study authors on 21 January 2021 but received no response.

**Table:** NTR4648

Methods	RCT
Participants	220
Interventions	1: Once daily versus twice daily use of 5‐ASA medication (Mezavant) 2: Interactive apps in UC patients on 5‐ASA medication (Mezavant) on adherence
Outcomes	Primary outcome: Compliance with 5‐ASA medication (Mezavant) objectively measured by presence of 5‐ASA metabolites in urine at 6 months Secondary outcomes: Adherence at 12 and 18 months Adherence by questionnaire Clinical as well as endoscopic and histological remission Safety QoL Costs and cost‐effectiveness
Notes	We contacted the study authors on 21 January 2021 but received no response.

5‐ASA: 5‐aminosalicylic acid; CG: control group; IBD: inflammatory bowel disease; IG: intervention group; QoL: quality of life; RCT: randomised controlled trial.

### Characteristics of ongoing studies [ordered by study ID]

**Table:** ACTRN12617000389303

Study name	Establishing the role of teleconsulting in the care of chronic conditions in rural areas of the Southern District Health Board (SDHB): a randomised controlled trial (RCT) in patients with inflammatory bowel disease
Methods	RCT
Participants	Target 75
Interventions	IG: teleconsulting + IBDsmart CG: standard medical care
Outcomes	Primary outcomes: Disease control measured by clinical disease activity indices Secondary outcomes: Cost effectiveness Acceptability
Starting date	1 April 2017
Contact information	Christine.Ho@otago.ac.nz Michael.Schultz@otago.ac.nz
Notes	We contacted the study authors on 20 January 2020. They told us the trial was ongoing with last patient out in April 2021 and that analysis and results could be expected afterwards.

**Table:** IRCT2020061304775

Study name	Evaluation of the effectiveness of mobile‐based inflammatory bowel disease management system by using gamification techniques on disease activity index, mental health and quality of life
Methods	RCT
Participants	210
Interventions	IG: education and disease management via mobile phone CG: standard care and routine outpatient clinics based on guidelines
Outcomes	Primary outcomes: QoL index Disease activity index Hospital Anxiety and Depression Secondary Self‐efficacy scale Non‐adherence
Starting date	22 November 2022
Contact information	narges.norouzkhani@yahoo.com
Notes	We identified this study during the update search

**Table:** NCT03985800

Study name	Specialty medical homes to improve outcomes for patients with IBD and behavioral health conditions
Methods	RCT
Participants	Estimated 990
Interventions	IG: TEAM‐care as usual approach CG: TECH‐telehealth approach
Outcomes	Primary outcomes: Disease severity Symptom severity Secondary outcomes: Functional impairment Healthcare utilization Self‐efficacy QoL
Starting date	1 July 2019
Contact information	meyersj5@upmc.edu
Notes

**Table:** NCT04207008

Study name	Trial of a decision support intervention for adolescents and young adults with ulcerative colitis (iBDecide)
Methods	RCT
Participants	42
Interventions	IG: iBDecide Decision Support Application CG: no intervention
Outcomes	Primary outcomes: Feasibility Acceptability Secondary outcomes: Decisional conflict Perceived shared decision making Decision preference congruence
Starting date	7 February 2020
Contact information	Ellen Lipstein, MD, MPH
Notes

**Table:** NCT04388865

Study name	Patient Automated Text Hovering for IBD (PATH‐IBD)
Methods	RCT
Participants	Estimated 150
Interventions	IG: clinical hovering CG: no intervention
Outcomes	Primary outcomes: SIBDQ response Secondary outcomes: Patient satisfaction Medication adherence
Starting date	23 February 2021
Contact information	Caitlin McDonald, MPH215-615-1571cmcdona@pennmedicine.upenn.edu Cathy Reitz, MPH215‐614‐0282catherine.reitz@pennmedicine.upenn.edu
Notes

**Table:** NCT04653259

Study name	Digital nutrition therapy for patients with IBD (LYFEMD)
Methods	RCT
Participants	44
Interventions	IG: LYFE MD app CG: conventional management
Outcomes	Primary outcomes: QoL Stress level Sleep quality Weekly physical activity minutes from both moderate and vigorous leisure‐time activity Well‐being and positive aspect Anxiety severity Depression Behaviour compliance Secondary outcomes: Diet quality FC Disease activity (HBI and partial Mayo score)
Starting date	15 May 2021
Contact information	mkothand@ucalgary.ca; lorian.taylor@ucalgary.ca
Notes	We identified this study during the update search.

**Table:** NCT04861597

Study name	Digital behavioral interventions in inflammatory bowel disease
Methods	RCT
Participants	50
Interventions	IG: internet‐based cognitive behavioral therapy (iCBT) CG: digital mood tracking
Outcomes	Primary outcomes: Psychological distress Health‐related QoL Secondary outcomes: individual process level barriers and facilitators to iCBT implementation (measured via surveys and semi‐structured interviews)
Starting date	27 April 2021
Contact information	rgreywoode@montefiore.org; rebecca.almonte@einsteinmed.org
Notes	We identified this study during the update search.

**Table:** Norton 2021

Study name	A supported online self‐management for symptoms of fatigue, pain and urgency/incontinence in people with inflammatory bowel disease: the IBD‐BOOST trial
Methods	RCT
Participants	680
Interventions	IG: facilitator supported online intervention for people who have expressed a desire for intervention for fatigue, pain and/or urgency/incontinence CG: standard care
Outcomes	Primary outcomes: UK Inflammatory Bowel Disease Questionnaire (UK‐IBDQ) and global rating of symptom relief at 6 months after randomisation Secondary outcomes: UK‐IBDQ at 12 months Rating of satisfaction with results of BOOST programme at 6 and 12 months Global rating of symptom relief at 12 months Numerical pain rating scale at baseline, 6 and 12 months after randomisation Vaizey (faecal) incontinence score, reflecting participants’ perceptions of severity at baseline, 6 and 12 months after randomisation IBD‐Fatigue score at baseline, 6 and 12 months after randomisation IBD‐Control score at baseline, 6 and 12 months after randomisation EQ‐5D‐5L general health‐related quality of life at baseline and 6 and 12 months after randomisation
Starting date	1 November 2017
Contact information	l.miller@qmul.ac.uk
Notes	We identified this study during the update search.

**Table:** RBR‐7t8fv7

Study name	Clinical trial of the effectiveness of telephone nursing care to individuals with inflammatory bowel disease
Methods	RCT
Participants	113
Interventions	IG: telenursing and nursing care CG: nursing care
Outcomes	Primary outcomes: Relapse Secondary outcomes: Hospitalisation
Starting date	
Contact information	Rachel Santos enfarachael@hotmail.com
Notes	

EQ‐5D‐5L: EuroQol five‐dimension, five‐level questionnaire; FC: faecal calprotectin; HBI: Harvey‐Bradshaw Index; QoL: quality of life; RCT: randomised controlled trial; SIBDQ: Short Inflammatory Bowel Disease Questionnaire.

## Differences between protocol and review

See Gordon 2021b (review protocol).

We updated our inclusion/exclusion criteria to clarify that we excluded studies where remote monitoring of blood or faecal tests was the only form of monitoring.

We added three outcomes: 'participant engagement', 'rate of attendance of interventions with healthcare professionals' and 'costs or cost/time‐effectiveness'. 'Participant engagement' focused on adherence to or compliance with the intervention specifically, and with 'attendance of interventions' we aimed to differentiate between planned and attended sessions. We added the costs outcome to collect any available quantitative data on costs or cost/time‐effectiveness.

We removed the outcomes 'change in disease activity' and 'change in quality of life' prespecified in the protocol, because we considered 'disease activity' and 'quality of life' to be sufficient.

We planned to remove cluster‐RCTs in a sensitivity analysis to assess their impact on the results. However, the only included cluster‐RCT provided no outcome data and was not included in any analysis (Siegel 2018).

In the protocol, we planned to conduct subgroup analysis by sex, but this was not possible due to a lack of data. Similarly, we were unable to conduct our planned sensitivity analyses for risk of bias and estimated standard deviations.

We had also planned to perform subgroup analyses based on age (adult/paediatric). However, we decided to present separate main analyses for adults and for children because of the significant differences of remote telehealth approaches for the two populations.

Based on peer review comments, we made some clarifications regarding how we intended to present our findings in the summary of findings tables.
